# TrajGNN-RL: Adaptive Mobile Sink Intelligence for Lifetime-Aware Wireless Sensor Networks

**DOI:** 10.3390/s26206444

**Published:** 2026-10-12

**Authors:** A. R. Aravind, Mohamad Yusoff Bin Alias, A. Deepak

**Affiliations:** 1 Department of Electronics and Communication Engineering, Prince Shri Venkateshwara Padmavathy Engineering College, Chennai 600127, India; ar123aravind@gmail.com; 2 Faculty of Artificial Intelligence and Engineering (FAIE), Multimedia University—MMU Cyberjaya, Persiaran Multimedia, Cyberjaya 63100, Selangor, Malaysia; 3 Saveetha School of Engineering, Saveetha Institute of Medical and Technical Sciences, Chennai 602105, India; deepakarun@saveetha.com

**Keywords:** mobile sink trajectory optimization, graph neural network, deep reinforcement learning, lifetime-aware routing, dynamic graph reconstruction, energy-aware routing

## Abstract

**Highlights:**

- To realize adaptive and lifetime-aware routing in dynamic wireless sensor networks, a hybrid approach combining topology learning with deep reinforcement learning (RL)-based trajectory optimization of the mobile sink (MS) was proposed to optimize the topology and the sink’s trajectory, respectively.
- A communication topology, residual energy distribution, and sink mobility feed-back–reconstruction mechanism are adaptively adjusted.
- Under dynamic mobile sink mobility scenarios, the proposed scheme outperformed the other schemes in terms of communication performance, giving a network lifetime of 98.34%, energy efficiency of 97.86%, throughput of 98.12%, and delay reduction of 96.75%.
- Based on comprehensive comparative and ablation experiments, the proposed framework of graph attention learning, adaptive sink routing plan, dynamic graph updating, replay-memory optimization, and multi-objective reward learning con-sistently outperformed existing mobile sink routing schemes.

**Abstract:**

Mobile wireless sensor networks require adaptive routing and sink mobility strategies to reduce energy imbalance, congestion, and communication delay. In this study, we propose a new method called TrajGNN-RL that combines the topology learning method based on a graph neural network with the deep reinforcement learning method to plan the trajectory of the mobile sink and route the packet in a coordinated manner. Dynamic communication graphs model the residual energy, link quality, neighborhood connectivity, and traffic conditions, and reinforcement learning optimizes the sink movement and forwarding decisions via a multi-objective approach. The graph changes during network operation to handle topology and traffic changes. The performance of the proposed routing scheme was compared with the performance of the evaluated routing schemes through experimental evaluation using varying numbers of sinks and varying sink mobility. The proposed TrajGNN-RL enhanced the normalized scores of the lifetime, energy efficiency, throughput, PkDel, routing reliability, and delay reduction by 98.34%, 97.86%, 98.12%, 98.57%, 98.83%, and 96.75%, respectively. The relative improvements in the normalized lifetime, energy efficiency, throughput, packet delivery ratio, and delay reduction, respectively, were around 1.47%, 1.55%, 0.68%, 1.19%, and 1.07%, compared to the best corresponding baseline used in the experiments.

## 1. Introduction

The use of a network of sensor nodes that are resource-constrained and communicate with each other in a distributed fashion is one of the most promising technologies for intelligent sensing and distributed data collection, known as mobile wireless sensor networks (mWSNs), since they can be used to monitor large geographical regions through the collaboration and communication between the nodes. The mWSNs are different from the static wireless sensor networks since one (or more) mobile sink nodes are used to collect information from distributed sensor devices, thereby minimizing long-distance communication and increasing scalability of the network. Their attributes make them ideal for use in smart homes, precision agriculture, the military, environmental monitoring, disaster management, intelligent transportation systems, smart city infrastructure, and healthcare monitoring [1]. With the proliferation of sensing applications with a high number of heterogeneous data, ensuring reliable communications and conserving limited battery life has become a key area of research. The performance and energy efficiency of the individual sensor node are usually the limiting factors in the lifetime of a sensing network in that the nodes typically use a non-rechargeable battery and are often deployed in inaccessible environments. Thus, intelligent routing mechanisms that can reduce communication overhead and maximize energy utilization to support sustainable wireless sensor network operation are more and more important [2].

Mobile sink technology was another major improvement over existing static-sink designs, as it spread the burden of communication over various parts of the sensing field, rather than focusing it on the permanent static sinks. To ensure an equal distribution of the mobile sinks in the network, periodically relocating themselves during the network’s operation, mobile sinks could help to reduce the distance between the mobile sinks and the users, mitigate the hotspot problem, distribute the network’s forwarding load, and enhance the efficiency of collecting the packets [3]. The mobility of the sinks, on the other hand, posed new problems that were related to the continuously varying network topology, dynamic neighborhood relationships, varying communication delay, unstable routing paths, and unpredictable traffic distribution. Each of the sink’s movements changed the connectivity of communication among the neighboring nodes, making the updates in routing decisions a continuous process to reflect the changing environment of the network [4]. Previous static wireless sensor network routing solutions could not handle these dynamic topology changes very efficiently, leading to more packet retransmissions, communication delay in the network, routing overhead, and early discharge of batteries. Hence, intelligent trajectory optimization and adaptive routing with a mobile sink have become imperative to fully utilize the benefits that mobile sink architecture provides.

Although routing is widely considered as the crucial issue in wireless sensor networks, recent advancements in artificial intelligence have introduced new opportunities for routing in wireless sensor networks [5]. Machine learning methods are used to automatically discover routing patterns based on their past observations in the network, without having to rely exclusively on manually coded routing rules in the system. Deep learning methods have shown great potential to capture the underlying communication features of large-scale networks, and reinforcement learning has shown that it can make autonomous decisions by interacting with a dynamic environment. Likewise, graph neural networks (GNNs) are also a significant learning model that can be used to model graph-structured information, with the ability to describe the relationships between graph components [6]. As wireless sensor networks are naturally formed as a graph structure, with the sensor nodes and the communication links between them creating a structure, graph-based learning offers an effective method for modeling the network topology, neighborhood interactions, communication quality, and traffic flow all in one. This development has spurred greater interest in developing and studying intelligent routing optimization based on graph representation learning for next-generation wireless communication systems.

With all these new technological developments, current mobile sink routing methods still have a number of salient deficiencies that hinder their real-world use and application [7]. Most of the traditional clustering algorithms (LEACH, PEGASIS, HEED, and EEUC) focus on saving energy by forming clusters and usually assume a fixed location of the sinks or a predetermined schedule for their movement. While these techniques decrease the amount of communication distance used when deployment starts, forwarders creating the same relay nodes create a series of energy holes around the cluster heads/sink nodes over time, thus decreasing the network’s lifespan [8]. In addition, the static clustering structures cannot adaptively adjust in an effective manner to the highly dynamic environments in which the communication topology changes constantly as a function of the sink’s mobility, leading to significant decreases in the packet delivery ratio in the highly dynamic sensing environment.

In order to enhance the trajectory planning of mobile sink, heuristic optimization methods such as Particle Swarm Optimization (PSO), Ant Colony Optimization (ACO), Genetic Algorithms (GAs) and Artificial Bee Colony (ABC) algorithms were introduced later [9]. These optimization techniques find the most efficient communication scheme of sink movement in terms of communication distance or even forwarding load distribution. Although heuristic methods are usually superior to static routing compared, they usually involve a number of iterations to arrive at a result and, under complex network situations, may get stuck on a local optimal solution [10]. Furthermore, their optimization process is generally conducted offline, which does not allow quick adaptation to sudden communication changes like sensor-node failures, changes in traffic generation or connectivity of the neighborhood. So, they are likely to make suboptimal routing decisions when the network is running for an extended period of time [11].

Recently, reinforcement learning (RL) has gained substantial interest due to its ability to adaptively learn the routing policy from an ongoing interaction with wireless communication environments [12]. It has been shown that Deep Q-Networks (DQNs), Deep Deterministic Policy Gradient (DDPG), Proximal Policy Optimization (PPO) and Actor–Critic methods can enhance the efficiency of packet forwarding and sink trajectory planning over the traditional heuristic approach. However, most of the reinforcement learning routing algorithms mainly take local information like the residual battery energy, the queue size or distance to communicate as observations when choosing routing actions [13]. These widespread phenomena are poorly represented by these local observations, limiting the ability of reinforcement learning agents to identify communication hotspots, develop congestion areas or detect a global energy imbalance of the sensing field. In turn, routing policies frequently do not take advantage of the topology of communications that can occur within the network.

To address some of these disadvantages, recently graph neural networks were developed to allow the analysis of communication along with neighborhood aggregation and graph message passing [14]. The routing methods based on GNNs can learn graph embeddings to capture the structural features of wireless sensor networks and model sensor connectivity, communication quality and multi-hop interactions to tackle these challenges [15]. The following learned representations are much better than the conventional feature extraction methods based on nodes for route awareness. Most current GNN-based routing schemes, however, require graph learning without graph trajectory optimization to the sinks [16]. While graph embedding gives useful topology information, it is seldom combined with adaptive decision-making frameworks that are able to dynamically adapt sink movement over time depending on the ever-changing communication situation. In the same way, static communication graphs are usually used to generate graph representations for a long period of time during communication time, which is not flexible enough when the sinks are mobile [17].

One more important drawback in the studies done so far is the independent optimization of the routing and sink trajectory planning. Most algorithms try to minimize one of the following: communication path selection or sink mobility, but they do not consider optimizing the other component when it is changing [18]. In real-world WSNs, however, the movement of the sink directly impacts the connectivity of the neighborhood, the traffic handled by the sensor nodes, the delay in communication and the energy consumed by the sensor nodes, and routing decisions affecting the next time sink movement are required [19]. The failure to consider this mutual dependency hinders the performance of communication systems from becoming optimally efficient in the world. Moreover, some existing solutions compute either one objective optimization metric (e.g., energy consumption or communication delay) or two or more optimization metrics (e.g., residual battery balance and energy consumption) but not all these metrics are taken into consideration simultaneously. This optimization approach is often done in isolation, which can optimize one network attribute while degrading another, making communicating more sustainable over the long haul more challenging [20].

To tackle those challenges, this study introduced a hybrid intelligent routing framework called TrajGNN-RL which combines the benefits of topology representation learning using the graph neural network (GNN) and mobile sink trajectory optimization using deep reinforcement learning (DRL). This framework for graph learning and policy learning in a continuous fashion differs from the current routing approaches where the learning of the graph and reinforcement learning are solved independently. The first step was to convert the wireless sensor communication data to the dynamic weighted communication graphs of the residual battery energy, the quality of the communication, the density of the neighborhoods, the amount of forwarding activity and the distribution of traffic. A graph neural network was used to obtain local communication and global network structure-based topology-aware embeddings. These graph embeddings were then combined with the state information of the mobile sinks and fed to a deep reinforcement learning agent that optimizes the trajectory of the sinks and routing decisions by using a multi-objective reward function. The learning framework could adapt to changing communication and network topologies, as well as the changing mobility of the sinks, by reconstructing the graph after each communication round. The experimental results have validated the proposed framework of TrajGNN-RL, which enhanced network lifetime, energy efficiency, throughput, routing reliability, communication delay compared with the existing mobile sink routing schemes, and it has been shown that the proposed solution is intelligent, scalable and lifetime-aware communication solution for next-generation wireless sensor networks.

### 1.1. Research Motivation

As mobile sink nodes allow flexible data collection, mobile wireless sensor networks (mWSNs) have been gaining significance in applications such as environmental monitoring, industrial automation, disaster management, precision agriculture, and smart city. The current mobile sink routing protocols, however, often require large energy consumption, high levels of congestion in the communications, unbalanced forwarding load and short network lifetime in dynamic mobility environments. Existing routing systems such as static routing cannot adapt quickly to dynamically changing network topologies and predefined sink-movement strategies cannot adapt quickly to the dynamically changing network structure. To address the limitations, a new approach emerged, an adaptive intelligent framework which can optimize the sink trajectory planning and routing decisions simultaneously by learning the graph topology and taking decisions based on reinforcement learning. Intelligent routing in the scope of this research is a routing process which is data-driven and adaptive, learning packet-forwarding decisions and the movement of the mobile sink based on the changing state of the wireless sensor network instead of only on predetermined forwarding rules, fixed routes and/or static heuristic criteria. The three capabilities of traffic information can be summarized as Dynamic Representation for Residual Energy, link quality, neighborhood connectivity, traffic condition, and sink mobility, Topology-aware Feature Learning and adaptive routing and Trajectory Decision-Making via DRL with multi-objective performance feedback.

### 1.2. Significance of the Study

In the field of intelligent wireless sensor networking, the proposed TrajGNN-RL framework plays a role in the development of the field by combining the ideas of graph neural network (GNN) and deep reinforcement learning (RL) to develop an adaptive mobile sink management system. The framework adapts its learning continuously to learn the communication topology, residual energy distribution and traffic dynamics and brings about more efficient routing for achieving longer network lifetimes. The proposed architecture is different from traditional routing solutions, which are independent of the mobility of the sinks, by jointly optimizing the trajectory planning and packet forwarding, in a single learning framework. The integrated solution brings benefits in terms of scalability, communication reliability, and energy efficiency and makes the solution ready for use in next-generation applications of the environmental monitoring, industrial Internet of Things, smart agriculture, infrastructure monitoring and disaster response.

### 1.3. Problem Statement

Many problems exist in existing mobile sink routing protocols which hinder them from being effective in a dynamic wireless sensor network. Static routing mechanisms are not dynamic enough and cannot adjust to the alteration of communication topology due to the mobility of sinks, leading to substantial delay and packet loss. In energy-aware routing techniques, it is common to have communication hotspots, and battery levels around the frequently used forwarding nodes can rapidly decrease. In the case of continuously changing traffic conditions and communication demands, the task of efficiently responding to the changing situation is not possible with heuristic trajectory planning algorithms. In addition, most commonly used solutions optimize routing or the placement of sink separately from each other, without taking into account the interactions between them, which leads to sub-optimal optimization of communication in wireless sensor networks in terms of global communication and therefore shortens network operational life, limits scalability and depresses network reliability.

### 1.4. Recent Innovations and Challenges

In the past few years, wireless sensor networks (WSNs) have been advanced with remarkable progress in graph neural networks (GNNs), reinforcement learning (RL), edge intelligence and intelligent wireless communication (IWC), which has greatly enhanced the adaptive routing capability of the WSN. Graph learning helps in the analysis of communication between the elements, taking into account their topology, while reinforcement learning helps in autonomous decision-making in dynamic settings. Despite this, there are still some issues that have not been resolved, such as always rebuilding the topology, optimizing both sink mobility and routing, ensuring energy balance, communication scalability and computational complexity of deep learning models. While the dynamic network conditions can be very challenging, the other two tasks of adaptivity and reducing communication overhead and network lifetime continue to be important research goals and motivators for further development of intelligent routing. The research questions are designed based on the major contributions of the proposed TrajGNN-RL framework:

- How do we represent learning in graphs that is dynamic to account for changing network topology, residual energy, communication quality, neighborhood connectivity and traffic conditions for topology-aware routing?
- How can the trajectory planning of the mobile sink be coordinated with the packet-routing decision in the whole system using the graph neural network and deep reinforcement learning?
- What are the benefits of dynamic graph reconstruction and adaptive sink movement in terms of energy utilization, packet delivery, throughput, communication delay and node survival, when the network topology changes?
- How well does TrajGNN-RL work with different network densities and sink mobility settings when compared to a conventional, an optimization-based and a learning-based routing approach?
- How can the overall performance of the TrajGNN-RL be improved by using graph attention, adaptive trajectory planning, dynamic graph updating, replay-memory optimization, and multi-objective learning?

### 1.5. Rest of Section of the Study

The remainder of this paper followed this organization. Section 2 introduced the existing work in mobile wireless sensor networks, mobile sink routing strategies, graph neural network, deep reinforcement learning, and energy-aware communication. The pros and cons of each existing research were discussed. In Section 3, we presented the proposed TrajGNN-RL framework, where we first explained how to prepare the dataset, construct the graph, learn the spatial features, optimize the trajectory of the mobile sink and introduce the intelligent routing and introduced an evaluation framework for the proposed framework. The experimental results and discussions are presented in Section 4, which is done through comparative analysis and ablations. Finally, Section 5 summarized the main conclusions and suggested the future research directions for the improvement of routing problem in intelligent mobile wireless sensor networks.

## 2. Related Works

A novel approach called Optimal Clustering and Network Topology for Mobile Sink-Driven Data Collection (OCNTMS) was suggested to enhance data collection in wireless sensor networks having non-uniform data generation. It optimized the rendezvous point selection, clustering, network topology and data forwarding by weight-balanced clustering, optimization of cost function, load-balanced link construction, and forwarding strategies [21]. Using simulation results, this approach reduced energy consumption by 18% and network lifetime by 40% compared with the existing approach. Results showed that network energy efficiency and data transmission performance were significantly improved.

A study examined the application of multiple mobile sinks to random mobility models for enhancing the quality of service and network lifetime of the wireless sensor networks on a large scale. Hierarchical data routing mechanism was used to balance energy consumption and minimize the total consumption of energy resources [22]. The simulation results showed that it had shorter transmission latency, higher reliability, better throughput, and more balanced energy distribution than the predefined mobile sink trajectory and conventional multi-hop routing. The results showed that the random mobile sink strategies were able to improve the performance of the network and energy efficiency in real-time applications.

To enhance network lifetime and quality of service, a hybrid wireless sensor network and Long-Term Evolution (LTE) architecture was designed for data collection with the support of mobile sink. A hybrid metaheuristic clustering algorithm and a Minimal Steiner Tree-based path scheduling algorithm with adaptive traffic class-based data scheduling algorithm were adopted in a two-stage optimization approach [22]. The integrated framework achieved optimum energy efficiency without affecting QoS. The simulation results showed an improvement of at least 6% in the network lifetime and a reduction of 26% in packet delivery delay over the existing methods, indicating the improved efficiency and data transmission performance of the simulation.

An artificial intelligence-based rendezvous point selection and rotation mechanism was suggested to achieve energy-efficient and fault-tolerant data collection in wireless sensor networks that are composed for IoT applications. An intelligent mobile sink scheduling scheme, which targeted energy-hole formation problem and network partitioning caused by hardware failure and limited battery capacity, was introduced [23]. The improvements in data collection efficiency, network reliability and energy consumption were shown by simulation and testbed evaluations. The results showed the proposed method could successfully increase network lifetime with reliable communications in resource-limited sensor networks.

In a wireless sensor network (WSN) with a consumer Internet of Things (IoT) device, an intelligent fault detection and mobile sink-based data collection mechanism was proposed. The method featured optimal mobile sink scheduling, hard fault detection and hyperparameter-tuned soft fault detection for effective identification of hardware and software faults. Inaccurate nodes were precisely identified, and energy loss, routing time and early network failure were minimized [24]. The simulation and testbed evaluations showed superior performance in terms of accuracy of fault detection, network lifetime, data collection rate, and routing performance over the current state-of-the-art approaches which resulted in increased reliability of consumer IoT applications.

To save energy and prolong the lifetime of wireless sensor networks, a scheme that intelligently routes data to the mobile sink in an energy-efficient manner was proposed. The framework used a novel clustering mechanism, prescheduled cluster head selection, a genetic algorithm-based mobile sink path optimization, low overhead in transmitting messages, and efficient data sensing, collection and transmission [25]. The effectiveness of the proposed approach was validated through the simulation and statistical analyses, which showed that the network lifetime, stability period, energy efficiency, throughput, latency and message overhead were all significantly improved over the latest state-of-the-art techniques.

To enhance the efficiency of data collection and sustainability of the networks, an intelligent routing protocol was designed for the mobile sinks in energy-aware wireless sensor networks [26]. The protocol used adaptive cluster formation and intelligent monitoring to optimize routing and reduce control overhead, latency and energy consumption. Through simulation, it was shown that the end-to-end latency could be reduced by 42% and the throughput increased by 38%, compared with the existing methods. Results showed that the protocol has good potential for Internet of Things-based smart monitoring applications with improved performance, reliable data delivery and network lifetime.

The advanced energy-efficient routing protocol, which was integrated with mobile sink and hybrid Gray Wolf Optimization and Particle Swarm Optimization framework for wireless sensor network, was described. A multi-objective strategy optimized cluster head and relay node selection based on Pareto principle using residual energy, sink proximity and sensor-node density [27]. The energy holes and communication overheads were minimized by following a predefined trajectory by the mobile sink. With the help of simulation, up to 50% increase in the network lifetime, 60% improvement in throughput and 40% reduction in energy consumption were shown when compared with current routing protocols, which improved energy efficiency and data delivery.

To improve the data collection in wireless sensor networks, a new mobile sink path planning-based energy-efficient routing protocol (ExAq-MSPP) was proposed. The sensor nodes deployed by Voronoi diagrams, clustered with LEACH algorithm, were optimized with Extended Aquila optimization algorithm according to delay, residual energy, link quality, priority and distance [28]. The results of the simulations showed that the delay was shorter, lifetime of the network was longer, packet delivery ratio was higher, remaining energy was greater and throughput was higher compared with the previous protocols. It was discovered that routing efficiency was improved, the path planning of mobile sinks was optimized and the network performance was strengthened.

A method of reducing data collection latency in multiple disjoint wireless sensor networks, an Efficient Relay Station Selection and Patch Optimization Algorithm (ERSS-POA), was proposed. The positions of relay stations were carefully selected with the help of the base station coordinates and communication range of the sensors to ensure network connectivity [29]. An ad hoc path optimization algorithm was used to obtain an efficient path for mobile sink data collection. The results of simulation showed that the wireless sensor network data collection efficiency and reliability was enhanced compared to the existing algorithms, as the packet loss, data collection latency, system throughput and network lifetime were reduced.

To enhance energy efficiency and prolong the lifespan of wireless sensor networks (WSNs) with static and mobile sinks, a new cluster routing protocol called Hybrid Termite Queen and Walrus Optimization Algorithm (HTQWOA) is proposed. The optimal cluster heads were determined by Termite Queen Optimization and for Walrus Optimization, optimal routing was obtained using multiple network parameters with energy-efficiency method. Multi-hop communication and hotspot formation was reduced with the mobile sink mobility [30]. The simulation results showed that the proposed method outperforms the existing optimization-based routing methods in terms of throughput, network lifetime, surviving nodes, and energy consumption, proving the effectiveness of the proposed method.

For the wireless sensor networks, a hybrid framework was suggested to enhance energy efficiency, data gathering and sensor charging in the wireless sensor networks through utilizing a standard mobile sink and data fusion. Rechargeable solar-powered sensor nodes, spectral clustering, artificial neural network (ANN)-based data fusion, and Dijkstra’s algorithm optimized network partitioning, anchor point selection, and sink trajectories were used. The PANUJA algorithm was used to effectively collect data when recharging sensor nodes [31]. The simulation results showed better energy efficiency, data reliability, fault tolerance, and performance than that of the traditional LEACH and tree-based flooding protocols, and this is suitable for smart city and remote sensing applications.

To improve data gathering and save energy consumption, a hybrid energy-efficient routing approach in mobile sink-based wireless sensor networks was proposed. The Improved Fuzzy C Means algorithm was used to cluster the sensor nodes, and Hybrid Glow-worm Swarm and Monarch Butterfly Optimization technique were used to select the cluster heads [32]. TDMA scheduling reduced the probability of data collisions, and an adaptive Euclidean Distance-based Crow Search algorithm was used to find the optimal routing. Through simulation results, it was found that the energy consumption was low, packet delivery ratio was high, packet loss was small, throughput was high and performance was better than the existing routing approaches.

A new framework using Adaptive Mobile Networks with a sensitivity analysis approach was given to enhance energy efficiency, load balancing and network stability in wireless sensor networks. The adaptive mobile sink scheduling and routing policy was learned with a Transformer-assisted Proximal Policy Optimization model under different scenarios [33]. The framework was tested with synthetic and real-world sensor data for node density, traffic intensity, and fault-tolerance effects. The results showed that such a scheduling method can provide better throughput, reliability, load balancing, and stable system operation than traditional scheduling methods, which are essential for supporting a resilient and scalable intelligent sensing system.

An optimization framework for the mobile sink mobility in wireless sensor networks (WSNs) in smart grid has been proposed based on reinforcement learning to achieve the maximization of network lifetime while maintaining high throughput. The following four sink strategies were tested: static, multiple, single and reinforcement learning [34]. Finally, a Pareto-optimal reward strategy using metaheuristic optimization enhances learning convergence and exploration. The reinforcement learning-based mobile sink enhanced the network lifetime from 33.5 to 36.15 months with higher throughput compared with the conventional static and multiple-sink methods while making the network more energy-efficient and energy-efficient.

Table 1 summarizes recent developments in mobile sink-assisted wireless sensor networks published. The literature surveyed shows that the current mobile sink routing approaches focus mainly on energy conservation, clustering, trajectory planning or routing optimization individually. The conventional and heuristic methods are not well-suited for rapidly changing topology, traffic and residual-energy conditions, and the learning-based method does not necessarily coordinate the movement of the mobile sink well, despite optimizing the routing. Furthermore, topology reconstruction where the topology is continuously updated, as well as the joint use of communication quality, energy distribution, neighborhood connectivity and traffic information, is still under-explored. In order to address these deficiencies, TrajGNN-RL is designed to integrate dynamic graph-based topology learning with reinforcement learning to make decisions on the sink trajectory and packet-routing. The study thus explores whether a constant reconstruction of graphs with a learning system that adapts communication policies could enable a more energy-balanced, reliable and mobility-friendly communication in changing wireless sensor network (WSN) environments. This motivation is in line with the identified gap in the manuscript about the separately optimized routing and sink placement.

When analyzed in the context of the dynamically changing mobile sink WSN, the reviewed approaches have several common limitations even though they are reported to have improved. Clustering and topology-oriented methods (like OCNTMS) mainly focus on optimizing rendezvous selection, clustering, topology and forwarding without continuously learning about the evolving topology and sink movements. The metaheuristic approaches such as GA-based, GWO-PSO-based, ExAq-based and HTQWOA-based schemes enhance the trajectory or routing decision but require iterative optimization and parameter-sensitive search which may limit the ability to quickly adapt to the changing of energy, traffic, sink position and connectivity. The manuscript itself points to these limitations of the computational and adaptability aspects of heuristic optimization.

Learning-based solutions offer some relief from this issue but have another drawback. RL-based mobile sink methods are capable to adapt their decisions by interacting with the environment, but decisions made primarily based on the local observations might not sufficiently capture the overall network congestion, energy imbalance and communication hotspots. In contrast to GNN, the topology-aware connectivity and multi-hop interactions are represented by GNN, and current routing schemes typically split off the task of learning the graph with the task of optimizing the sink trajectory to the base station and sometimes use a static communication graph that is not sensitive to sink mobility. So, the main constraint is not that there are no routing, mobility or learning mechanisms but the lack of adaptation for their combination. To tackle this limitation, TrajGNN-RL reconstructs the communication graph as the network changes status, acquires the topology-aware embeddings of nodes through the GNN, and provides the embeddings to the DRL agent for making coordinated sink-movement and packet-routing decisions based on a multi-objective reward. Such a direct feedback loop among topology change, graph representation and routing action, sink mobility and network-state reconstruction follows, instead of optimizing each of these components separately.

## 3. Proposed TrajGNN-RL Framework

**Figure 1:**
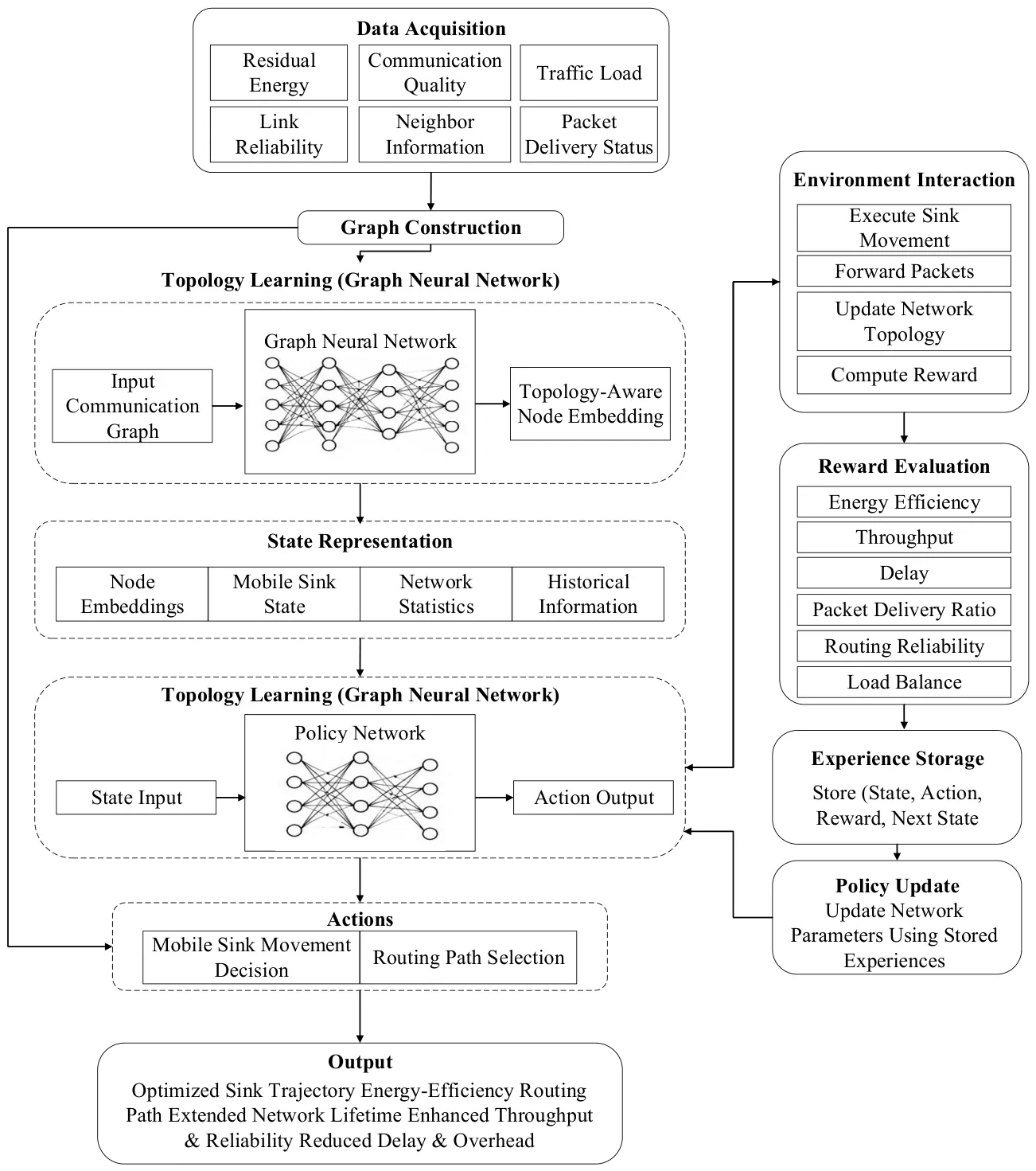
Proposed TrajGNN-RL architecture for lifetime-aware mobile sink routing.

The proposed TrajGNN-RL framework first collected and pre-processed wireless sensor communication data to build a dynamic graph to describe the connectivity graph between nodes, as well as the energy remaining in nodes and the quality of communication between nodes. A graph neural network (GNN) was used to learn the spatial relationship between neighboring sensor nodes, to obtain the topology-aware embeddings. These graph embeddings, along with the mobile sink state, were fed into a deep reinforcement learning (DRL) agent which optimizes the trajectory of the sink as well as the decision on routing, using a multi-objective reward function. The communication graph was continually updated following every communication cycle, so that the routing policies could be continually refined. Lastly, the framework was checked under different network conditions considering the following parameters: network lifetime, energy efficiency, throughput, communication delay, and routing reliability. The architecture of the proposed TrajGNN-RL is depicted in Figure 1, which includes the communication data acquisition, construction of the graph, learning the graph neural network, optimizing the trajectory with the deep reinforcement learning, making adaptive routing decisions, updating the policy, and finally, the energy-efficient lifetime-aware performance of the wireless sensor network.

### 3.1. Multi-Hop Wireless Sensor Dataset Acquisition and Network Graph Construction

The proposed TrajGNN-RL framework was developed using the Suthaharan Multi-Hop Wireless Sensor Network (WSN) Dataset, which consists of the wireless sensor communication traces collected from the real multi-hop wireless sensor deployment using TelosB sensor motes. The dataset was made publicly available for machine learning research and involved measurements of communication that were taken from a set of low-power IEEE 802.15.4 [18] sensor nodes under realistic wireless conditions. It includes the transmitting node identification, the receiving node identification, packet sequence number, Received Signal Strength Indicator (RSSI), Link Quality Indicator (LQI) and a number of other values for the environment being sensed, the communication reliability, and the timestamp. All these properties are representative of the node-to-node interactions that are needed to learn the adaptive routing behavior in mobile wireless sensor networks. The dataset was downloaded from the original repository, known as the official Stdlib repository, which stores all communication records as they were.

**Figure 2:**
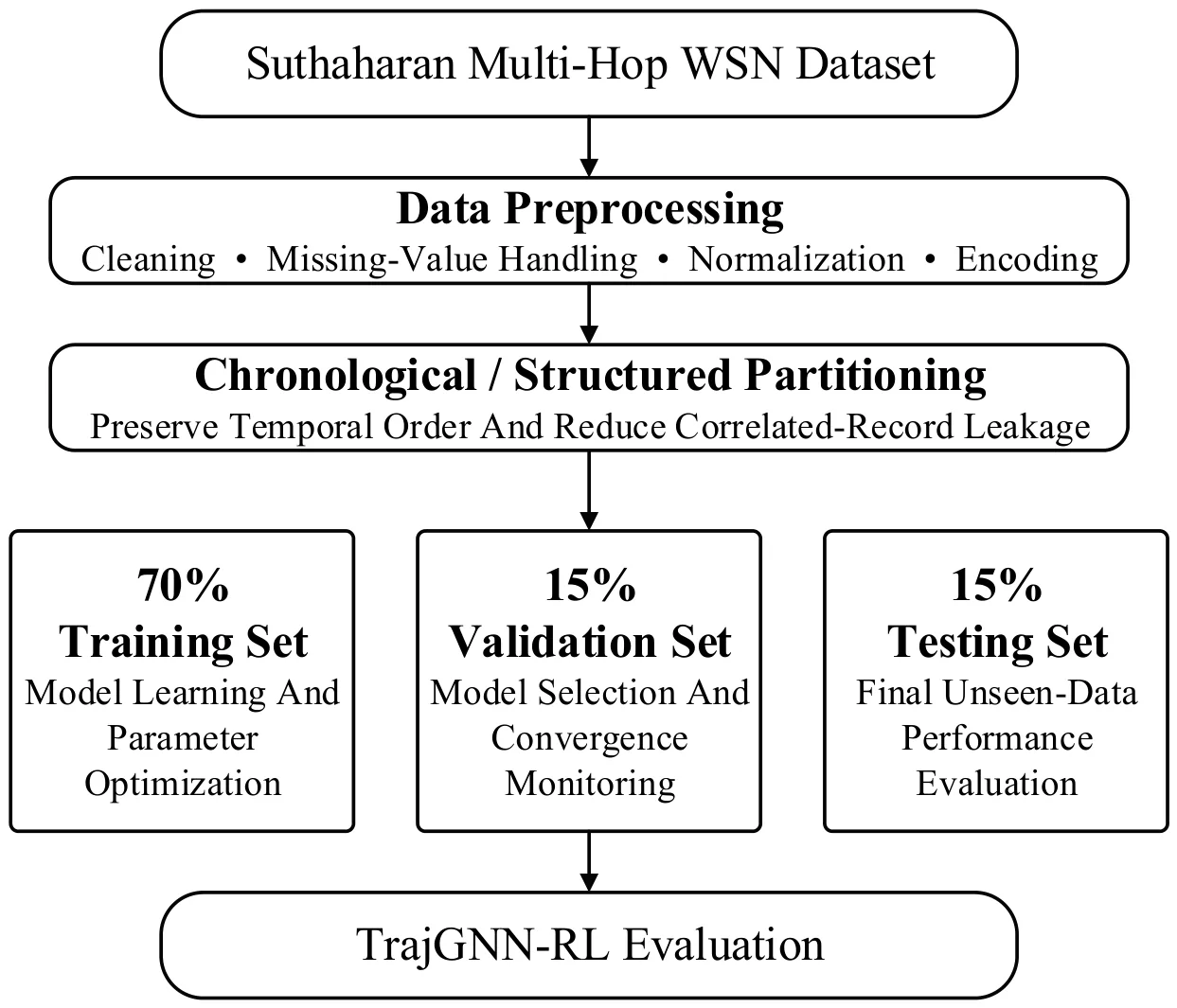
Dataset partitioning sequence for TrajGNN-RL.

To enhance the stability of learning, data cleaning was carried out by removing data that were not fully recorded, duplicated data, invalid data with timestamps and corrupted data from the sensor data. The RSSI and LQI data of the nodes that were not detected were filled with mean value interpolation within their neighborhood to ensure the continuity of communications between neighbors. To avoid the imbalance of the feature scales as shown in the graph learning, we applied Min–Max normalization to obtain a common numerical range for various features such as transmission distance, residual battery level, packet generation frequency, communication delay, RSSI and LQI. The ID numbers for each sensor and the labels used for communicating were encoded in integers prior to the construction of the graph. Random record-level shuffling was not performed in the final evaluation because it could cause information leakage due to temporal and topological correlations on the communication traces of the WSNs. A balanced 70/15/15 train/valid/test partition was implemented with three holdout constraints. First, temporal holdout maintained the temporal order of the communication intervals, thus excluding later communication intervals from training. Second, topology-based holdout divided a set of neighborhoods from the training graph (the ones excluding a node and its neighbors) to test how well the network routed when the topology was slightly altered. Third, unseen-scenario holdout reserved combinations of traffic load, sink location, mobility condition and communications quality for testing purposes. As a result, the closely correlated records could not be split between training and test subsets, which made the evaluation of the generalization capacity of TrajGNN-RL under unforeseen temporal, topological and operating conditions more challenging. To minimize information leakage and enable robust evaluation of the TrajGNN-RL, the preprocessed Suthaharan Multi-Hop WSN dataset is chronologically and structurally split into 70% training, 15% validation, and 15% testing sets, as shown in Figure 2.

The Suthaharan Multi-Hop WSN traces were then incorporated into the simulated environment using the transmitter/receiver identities, RSSI, LQI, packet sequence and timestamp information to describe empirical node interactions and the quality of wireless links. These measurements, along with the residual energy, queue occupancy, forwarding activity, node-to-sink distance, neighborhood density, packet delay, and traffic load generated by the simulations, were combined to create the node features and dynamic weighted communication graph. The graph was built anew after each round of communication, based on the most recent node and mobile sink states. This makes an explicit link between the real communication traces and the dynamic network simulation and not a complete mobility trace. The simulation employed a 50 m communication range, 25 m sensing range, 512-byte packets, 2J initial node energy, and traffic rates varying from 2 to 10 packets/s. For reproducible energy accounting, the radio model used $E_{\mathrm{elec}}=50\;\mathrm{nJ}/\mathrm{bit},\epsilon_{f_{s}}=10\;\mathrm{pJ}/\mathrm{bit}/m^{2},\;$ and *ϵ_mp_* = 0.0013 pJ/bit/m^4^. The GNN was trained using link-reconstruction loss with 10^−4^ L2 regularization. The normalized multi-objective reward was defined as *R_t_* = 0.25*PDR_t_* + 0.25*EE_t_* + 0.20*TH_t_* − 0.15*D_t_* − 0.10*RO_t_* − 0.05*SD_t_*, prioritizing packet delivery and energy preservation while penalizing delay, routing overhead, and unnecessary sink movement. Five independent runs used seeds 42, 123, 2023, 3407, and 7861. Training comprised 1500 episodes of 500 communication rounds, with mobile sink speeds varied from 1 to 10 m/s.

The communication traces for the Suthaharan Multi-Hop WSN dataset are gathered from a real deployment of TelosB-based multi-hop wireless sensors; they have a high degree of real-world realism. Measured RSSI, LQI, packet sequence, transmitter/receiver interaction and temporal information capture practical variations in the quality of the wireless-link and communication behavior. This variability was expanded by simulating different scenarios changing (in addition to the empirical traces) the node densities, packet generation rates, sink positions and velocities, communication conditions, and network topologies. Temporal, topology-based, and unseen-scenario holdouts were also used to investigate the generalization to out-of-scenario situations. However, no dataset can address the complete variations in the WSN deployment, which includes the variation in sensor hardware, irregular terrain, long-term environmental changes, persistent interference, failure of physical nodes, and conditions in a large field.

After preprocessing, the communication records were cleaned up to yield a graph structure for learning with graph neural network which was suitable for the specific task. Each wireless sensor node is considered as an individual graph vertex, and communication between neighboring nodes is considered as weighted graph edges. The edge weights were measured based on the combination of communication distance, RSSI, LQI, success rate of the packet delivery during the last few transmissions and the frequency of the transmissions, giving a combined representation of the wireless communication quality, and not just connectivity. The residual energy, the load of the transmission, the queue occupancy of packets, the degree of communication, the frequency of forwarding, the average delay of packets, the proximity of the sink and the neighborhood density of the node were combined to form the feature vectors of the node. The ability to capture both the local communication behavior and the global network characteristics was made possible by these node attributes that are multidimensional.

The created graph maintained the dynamic communication graph among the sensor nodes considering different mobility scenarios of the sink, while traditional routing algorithms, based on node coordinates or shortest hop, only provided the graph of static neighbors. The topology of the graph was updated after each round of communications with all the energy observed as residual, forwarding activity, packet delivery statistics and the sink position. Therefore, the network representation was continuously updated as a result of the varying wireless conditions and not a static representation during simulation. The subsequent learning framework could detect congested communication regions, patterns of energy depletion, unreliable wireless links and incipient routing bottlenecks with a significant level of success with this adaptive graph construction prior to starting trajectory optimization. The proposed TrajGNN-RL architecture used the generated graph as the basic input and laid the groundwork for the mobile sink trajectory planning and adaptive routing optimization of intelligent mobile sink.

### 3.2. Graph Neural Network-Based Spatial Representation Learning

The proposed TrajGNN-RL framework first built the communication graph and then extracted the spatial features of wireless sensor nodes by a graph neural network (GNN) to learn the complex relationship between the sensor nodes. The adopted graph neural network comprised three graph convolution layers, a graph attention refinement layer and a fully connected embedding layer. The graph convolution layers each had 128 hidden neurons and were able to sum together the information they received from their neighboring sensor nodes by passing messages around iteratively. To avoid the problem of gradient saturation when training the model and to ensure that the communication patterns remained nonlinear, the Rectified Linear Unit (ReLU) activation function was used as an activation function after each convolution layer. The introduced batch normalization layer was placed after every graph convolution layer to make the distribution of the features more stable and to have the optimization process to converge faster. This dropout rate (0.30) was also added to prevent overfitting due to the network of repeated communication structures of the wireless sensor graph. The graph attention layer then gave different attention scores to each neighbor node based on the reliability of communication, battery residual, workload of forwarding packets and consistency of packet delivery. As a result, more stable wireless links had a greater impact on the learned node representation than less reliable wireless links.

In each iteration of message passing, each sensor node gathered information of the hidden features from its direct neighbors and gradually passed on the learned features to neighboring nodes over multiple hops. To achieve this, three iterations of message passing were conducted for each graph update, so that each node can be updated with information from remote parts of the network without creating too much “burden” on the running of the program. The framework could detect communication bottlenecks across the globe, the highly loaded forwarding nodes, the less dense sensing regions and the possible formation of an energy hole which could not be detected with a traditional localized routing algorithm. Following the aggregation phase, the new node features were then combined with the original feature vector of the nodes and then passed to the embedding layer, thus keeping the raw sensor information along with the new structural information.

The Adam optimizer with an initial learning rate of 0.001, weight decay of 0.0001 and mini-batch size of 64 graph samples was used to train the graph neural network. The model was trained over 150 epochs but, due to the validation performance getting stable after 15 epochs, the model was not trained unnecessarily beyond that point. The gradient clipping method was performed to avoid gradient explosion in deep graph propagation; the threshold value of 1.0 was set. The number of features in the hidden embedding dimension output from the last layer was set to 256, which covered each sensor node, but was still very informative of the state of the entire network. These embeddings captured the quality of the communications, their route importance, the amount of energy available, the level of congestion, the forwarding behavior and the influence of the neighborhood into a single feature space which was interpretable by the reinforcement learning agent with ease. The communication graph was dynamically rebuilt after every cycle of communication after every node transmission with the updated states of the nodes and newly formed wireless links. To avoid the start-up of re-training the entire graph neural network from scratch, the weights previously learned were kept and were only incrementally updated by the most recent communication graph. This incremental learning approach also greatly lowered the computation burden and enabled the spatial representations to continuously change as the topology shift occurred because of sink moving, node energy depletion or changing traffic. The mobile sink moved through various parts of the sensing field, which led to dynamically changing node embeddings based on the new communication routes in the neighborhood and changing neighborhood structures.

**Figure 3:**
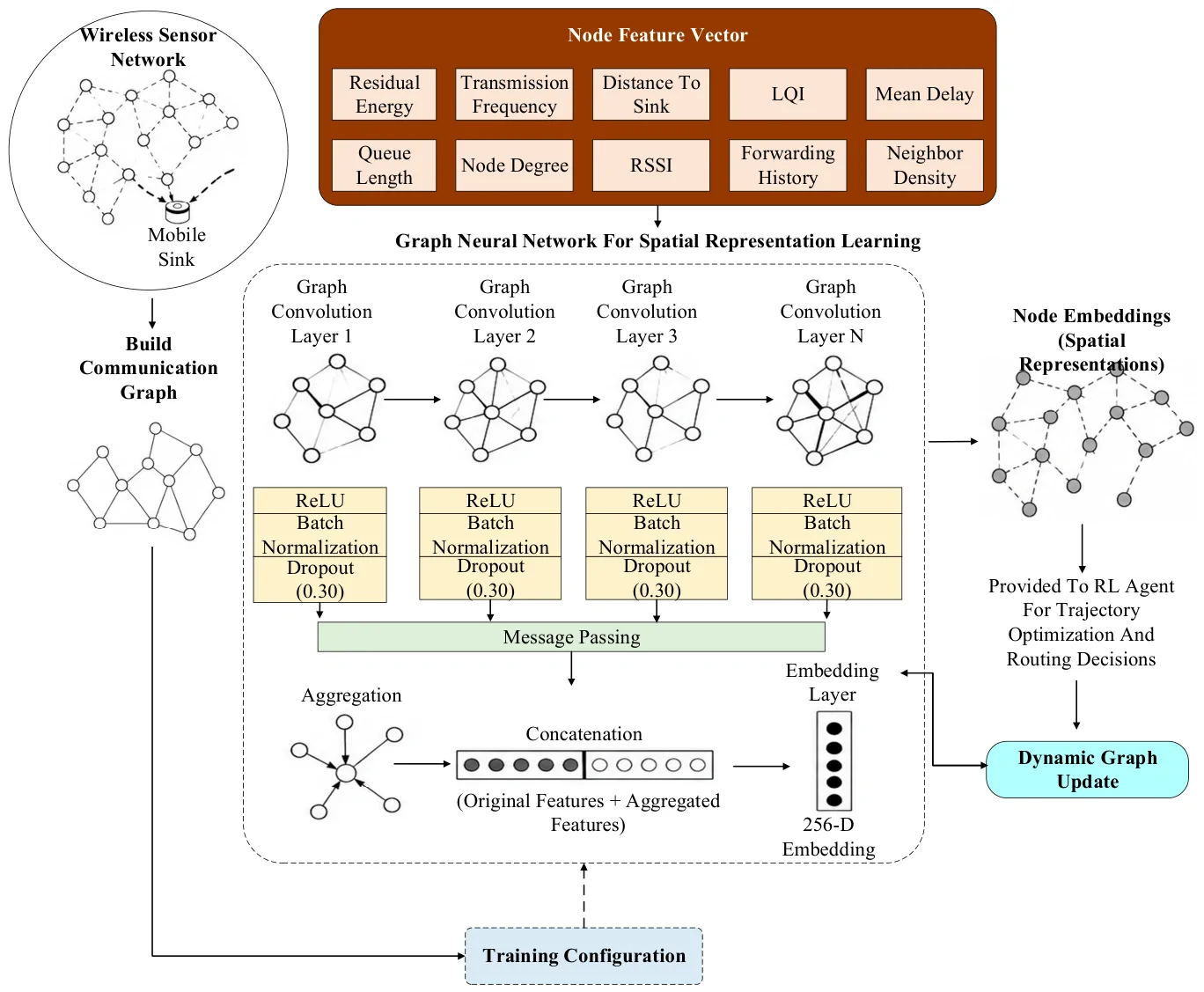
Graph neural network-based spatial representation learning framework.

A unique aspect of the proposed TrajGNN-RL framework was the combination of graph-level intelligence and trajectory optimization. The graph neural network (GNN) was used to extract high-level structure information of nodes instead of only node statistics and capture the hidden communication relationship between them in the whole wireless sensor network. The ability to make reinforcement learning decisions could be based on comprehensive global network awareness because of these embeddings instead of being based on just local observations. The proposed graph learning mechanism significantly reduced the number of congested zones, the communication reliability, the distribution of the residual energy and the number of the upcoming routing opportunities, which then laid the base for the next piece in the puzzle—mobile sink trajectory optimization. As shown in Figure 3, the proposed spatial representation learning framework is based on graph neural network (GNN), which includes constructing the communication graph, aggregating the features, performing graph attention learning (GAL), generating the embeddings, updating the graph dynamically, and integrating the reinforcement learning.

### 3.3. Lifetime-Aware Mobile Sink State Modeling and Dynamic Environment Representation

The proposed TrajGNN-RL framework builds an environment state vector over the lifetime, which combined communication topology, residual energy distribution, packet generation dynamics, traffic congestion, forwarding load, and mobile sink mobility. This environment representation is changed after each communication round, so that trajectory optimization is done based on the current network state, not the routing information at an earlier time. The sensing field is the area of deployment, which is square in shape, and has a dimension of 1000 m × 1000 m with 200 wireless sensor nodes deployed randomly with a uniform space distribution. The packet size was fixed at 512 bytes, and each sensor node was equipped with an initial battery of 2 Joules. Standard experimental setup had a communication radius of 50 m and a sensing radius of 25 m. The default communication radius used in the model training, the primary performance evaluation and comparative experiments with baseline routing methods was 50 m. The communication range used in the sensitivity experiments was fixed at 50 m unless otherwise stated and all the reported results are within this communication range. The mobile sink first randomly moves inside the deployment area and continuously moves the mobile sink through the sensing field following the decisions made by the reinforcement learning agent. Velocity of the sink was dynamically changed from 1 m/s to 10 m/s, representing different mobility applications of the environment, such as environmental monitoring applications and high speed for inspection systems in industry. Multi-hop forwarding was used for the communication between sensor nodes; when direct communication to the sink was not possible it was used to represent the real situation in wireless sensor networks.

Equation (1) computed the complete state representation of every wireless sensor node before graph embedding generation.

(1) $$S_{i}=\left[E_{i},Q_{i},D_{i},R_{i},L_{i},H_{i},N_{i},T_{i}\right]$$

where *S_i_* represented the complete state vector of the *i*th sensor node, *E_i_* denoted the residual battery energy, *Q_i_* represented packet queue occupancy, *D_i_* denoted the transmission delay, *R_i_* represented the Received Signal Strength Indicator (RSSI), *L_i_* denoted the Link Quality Indicator (LQI), *H_i_* represented the hop distance to the mobile sink, *N_i_* denoted the neighborhood sensor-node density, and *T_i_* represented the packet generation rate.

The first-order radio model was selected for packet transmission and reception to obtain a physically interpretable model of energy over the lifetime. The model considers the consumption of radio-electronic energy and distance-dependent power amplifiers. It was assumed that multipath fading would occur for distances greater than *d*_0_ and free space propagation would take place for shorter distances. Thus, the energy needed to send an l-bit packet a distance *d* was defined as

(2) $$E_{\mathrm{TX}}\left(l,d\right)=\left\{\begin{array}{c} lE_{\mathrm{elec}}+l\varepsilon_{\mathrm{fs}}d^{2},\;d<d_{0}\\ lE_{\mathrm{elec}}+l\varepsilon_{\mathrm{mp}}d^{4},\;d\geq d_{0} \end{array}\right.$$

Additionally, reception energy was calculated as:

(3) $$E_{\mathrm{RX}}\left(l\right)=lE_{\mathrm{elec}}$$

With the distance threshold:

(4) $$d_{0}=\sqrt{\frac{\varepsilon_{\mathrm{fs}}}{\varepsilon_{\mathrm{mp}}}}\;$$

For data aggregation, where applicable,

(5) $$E_{\mathrm{DA}}\left(l\right)=lE_{\mathrm{DA}}$$

The residual energy of sensor node *i* after communication round *r* was consequently updated as:

(6) $$E_{i}^{t+1}=E_{i}^{t}-\left(E_{\mathrm{tx}}+E_{\mathrm{rx}}+E_{\mathrm{idle}}\right)$$

where $E_{i}^{t}$ represented the current residual energy, $E_{i}^{t+1}$ represented the updated residual energy after transmission, *E_tx_* denoted transmission energy consumption, *E_rx_* represented reception energy consumption, and *E_idle_* denoted idle listening energy.

Each sensor node at the start of the communication intervals produced a high-level state vector describing the current state of the node. This state vector contained residual battery energy, queue size of packets, transmission rate, number of hops to current sink position, number of neighboring sensor nodes, Received Signal Strength Indicator (RSSI), Link Quality Indicator (LQI), packet delivery success ratio, density of neighboring sensor nodes, channel utilization and historic communication reliability. All these characteristics embodied the local operational characteristics of each sensor node and simultaneously the role of the sensor node in the global communication system. These sensor-node state vectors together reflect the operational status of the wireless sensor network and the current information of the environment that is needed for adaptive trajectory optimization.

(7) $$w_{\mathrm{ij}}=\alpha_{R}{\tilde{\mathrm{RSSI}}}_{\mathrm{ij}}+\alpha_{L}{\tilde{\mathrm{LQI}}}_{\mathrm{ij}}+\alpha_{P}\mathrm{PD}R_{\mathrm{ij}}+\alpha_{D}\left(1-{\ddot{d}}_{\mathrm{ij}}\right)\;$$

where

(8) $${\tilde{\mathrm{RSSI}}}_{\mathrm{ij}}=\frac{\mathrm{RSS}I_{\mathrm{ij}}-\mathrm{RSS}I_{\min}}{\mathrm{RSS}I_{\max}-\mathrm{RSS}I_{\min}},\;\ddot{d}=\frac{d_{\mathrm{ij}}}{d_{\max}}$$

And

(9) $$\alpha_{R}+\alpha_{L}+\alpha_{P}+\alpha_{D}=1,\;\alpha_{k}\geq 0$$

The score of the communication link is calculated on normalized RSSI, LQI, packet delivery ratio and communication distance and is computed using Equation (7). RSSI in dBm is normalized before combination, which results into the values being presented in a logarithmic scale potentially with negative values and preventing the direct multiplication with the heterogeneous link quality measurements. As a result, *w_ij_* is deemed to be a more reliable and shorter communication link.

The proposed framework also proposed a dynamic mechanism for neighborhood update throughout the operation of the network to further enhance adaptability. The neighbor relations between the sensor nodes were automatically re-calculated as the mobile sink moved from one place to another and/or the communication links changed due to interference, energy depletion and/or packet congestion. Those nodes that were beyond the range of communication had their links cut off and those nodes that were newly created and linked had their links added to the graph representation. This continual topology reconstruction thus allowed the communication information to remain up to date and prevented the reinforcement learning agent from making routing decisions on outdated communication information. At the same time, node priorities were updated based on the battery capacity, forwarding load, number of accumulated packets, communication stability and the distance from the moving sink. Therefore, in regions that suffer from too much traffic and/or battery depletion, it was quickly determined that the routing optimization was not needed and it could be avoided.

Equation (10) calculated the neighboring communication density surrounding each sensor node.

(10) $$N_{i}=\sum_{j=1}^{n}A_{\mathrm{ij}}$$

where *N_i_* represented the neighborhood density of sensor node *i*, *A_ij_* denoted the adjacency relationship between nodes *i* and *j*, and *n* represented the total number of sensor nodes.

The mobile sink was represented by an adaptive state descriptor that records the spatial location, traveling speed, direction, accumulated traveling distance, density of the surrounding communication nodes, average packet collection rate, energy collection efficiency of the current region where it is located and the frequency of its visits to different regions for sensing. Additionally, the proposed framework did not just pick motion choices from geographical details but assessed the impact of sink motion on the encompassing communication environment. For instance, moving towards a region with high population of sensors with evenly distributed residual energy was preferred over moving towards regions where the energy of the sensors was already depleted. Likewise, during the policy optimization process, sink trajectories that resulted in the least average number of hops, but with no more than the necessary amount of communication overlap was deemed more helpful. The environment-aware sink representation allowed the reinforcement learning agent to greatly enhance its learning efficiency in the face of highly dynamic wireless communications in this environment.

A traffic estimation module that adapts to traffic conditions was also added to the traffic environment to model the temporal variation in sensing activity. In every round of communications, packet generation rates were monitored for every sensor node in the network, thereby identifying low, medium and high traffic regions in the network. Equation (11) evaluated communication congestion around every forwarding region.

(11) $$C_{i}=\frac{Q_{i}}{B_{i}}$$

where *C_i_* represented the congestion index, *Q_i_* denoted packet queue occupancy, and *B_i_* represented the available communication bandwidth.

The proposed TrajGNN-RL environment representation integrated structural graph intelligence, reliability and mobility attributes of sinks, traffic evolution, neighborhood dynamics and forwarding behavior into an adaptive state model, which differs from previous mobile sink routing frameworks that only considered the positions and battery levels of nodes. This multi-dimensional representation was constantly updated during network operation, providing an accurate description of both the current state of the network, and of what the network would be like in a future time step. This enabled the subsequent trajectory optimization decision to be made using global network information, instead of local observations, laying the groundwork for intelligent decision-making for adaptive trajectory optimization of the mobile sink and lifetime-aware wireless sensor network management.

Equation (12) represented the dynamic operating state of the mobile sink.

(12) $$M_{t}=\left[X_{t},Y_{t},V_{t},\theta_{t},D_{t}\right]$$

where *M_t_* represented the sink state vector at time *t*, *X_t_* and *Y_t_* denoted sink coordinates, *V_t_* represented sink velocity, *θ_t_* denoted movement direction, and *D_t_* represented cumulative traveling distance. Equation (13) estimated the communication importance of every sensing region.

(13) $$P_{r}=\beta_{1}{\tilde{Q}}_{r}+\beta_{2}{\tilde{N}}_{r}+\beta {\tilde{E}}_{r}-\beta_{4}CV_{E,r}$$

where

(14) $$CV_{E,r}=\frac{\sigma_{E,r}}{\mu_{E,r}+\epsilon }\;$$

Additionally, *Q_r_*, *N_r_*, *E_r_* and *CV_E_*_,*r*_ denote normalized queue occupancy, sensor-node density, mean residual energy, and energy variation within region *r*, respectively. High priority is assigned to a region if it has accumulated traffic, high density of sensor nodes and uniform distribution of residual energy. The energy-variation penalty restricts the mobile sink from repeatedly choosing severely depleted nodes. This formulation therefore gives priority to the mathematical formulation of the objective of balanced energy use.

**Figure 4:**
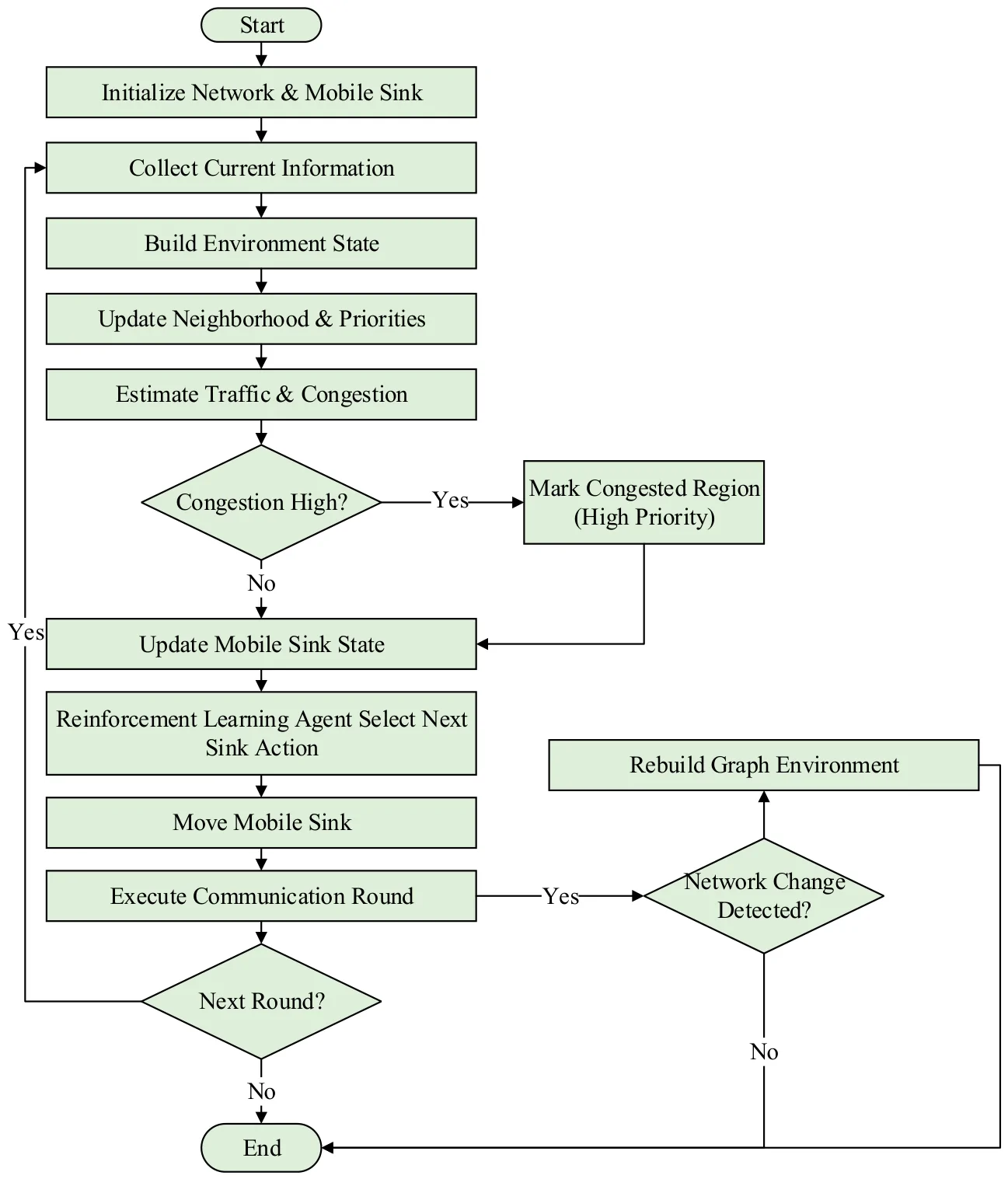
Lifetime-aware mobile sink state modeling and dynamic environment representation.

Equation (15) generated the final environment representation supplied to the TrajGNN-RL framework.

(15) $$\Omega_{t}=\left\{S_{t},M_{t},G_{t}\right\}$$

where *Ω_t_* represented the complete environment state at time *t*, *S_t_* denoted the collection of all sensor node state vectors, *M_t_* represented the mobile sink state vector, and *G_t_* denoted the dynamically constructed communication graph used for graph neural network learning. The adaptive environment modeling workflow is shown in Figure 4, including the generation of the environments, updating the neighborhood, estimation of the traffic, evaluation of the environments for the sinks, reconstruction of the graph dynamically and continuous feedback for optimizing the trajectories.

### 3.4. TrajGNN-RL Hybrid Learning Framework for Adaptive Mobile Sink Intelligence

The proposed TrajGNN-RL framework proposed the combination of the following two feature extractor modules: graph neural network (GNN) and deep reinforcement learning (DRL) to design an adaptive decision-making architecture which can optimize the mobile sink motion and multi-hop routing in wireless sensor networks (WSNs). The suggested framework jointly learned the network topology, the quality of the communication, the distribution of the energy levels of the nodes and the sink trajectory in a single learning environment, unlike the other traditional methods that optimized the routing or the sink trajectory separately. The hybrid architecture was used to provide continuous exchange of information between the graph learning module and the reinforcement learning module and thus the decisions of the sinks were dynamically modified as a function of the varying communication conditions, as opposed to using a mobility model. The first one is dynamic communication graph generated in the previous stages which is given as the main input to the graph neural network. In each graph, the feature vectors were the residual battery energy, the queue length of packets at nodes, the number of times that a node has forwarded a packet, the number of neighbors received by a node, the distance of a node to the sink, the Received Signal Strength Indicator (RSSI), the Link Quality Indicator (LQI), the delay between a node sending a packet and it being received, the packet’s delivery ratio up to this point, the density of the neighbors received by a node, the load of traffic received by a node, and the proportion of nodes using their transmissions. All node embeddings produced by the graph neural network captured the local communication properties as well as higher-order structural dependencies that were created by repeated message propagation. In contrast to conventional graph embedding techniques, the proposed framework continuously adapted node embeddings based on the network’s topology changes at each communication interval, thus allowing continuous adaptation to changes in the topology due to mobile sink’s motion, energy depletion and wireless-link fluctuation.

The graph neural network was made up of three graph convolution layers, one graph attention refinement layer and one embedding projection layer. A 128-to-128 hidden-neuron graph convolution network (GCN) was used for each graph convolution layer, and a graph attention layer (GAL) with eight parallel attention heads was used to learn simultaneously to consider the importance of communication based on different neighborhood perspectives. A projection layer was embedded to get a 256-dimensional latent feature vector for each sensor node, and this compact representation was provided to the reinforcement learning algorithm. After each convolution, the Rectified Linear Unit (ReLU) activation function was used, and the data distributions were stabilized during training by the use of batch normalization. With a 0.30 dropout probability, overfitting problem due to the repetitive communication structure, which is typical in the wireless sensor network, was prevented. The Adam optimizer was used, using a starting learning rate of 0.001, weight decay of 0.0001, amini-batch size of 64 and early stopping for 15 successive epochs that did not improve the validation loss. The graph propagation was very deep, causing problems in updating the parameters, which was solved by gradient clipping at 1.0.

The mobile sink state representation was then extended with the created graph embeddings to create the mobile sink observation space for the reinforcement learning agent. In contrast to the traditional reinforcement learning models that only used node coordinates or residual battery capacity, the proposed observation vector included not only the information of the sink location, movement direction, traveling velocity, communication density, packet generation statistics, etc., but also the information of the communication reliability of the graph, forwarding congestion, average transmission delay, packet delivery ratio, neighborhood energy balance, queue occupancy, and graph-level communication reliability. The reinforcement learning agent was provided with a detailed overview of the entire wireless sensor network, rather than merely with information from a single node, leading to much more informed decision-making when making trajectory decisions.

The decision process for communication round *t* was represented by a structured joint action *a_t_* = (*m_t_*, *r_t_*), where *m_t_* is the decision made by the mobile sink and *r_t_* is the packet-routing decision. The sink-movement space was defined as 10 separate actions as follows: north, south, east, west, diagonal (NE, SE, SW, and SE), still, and approaching the identified traffic hotspot. The routing module chose a capable neighbor forwarding node based on the existing GNN representation, which took into account the residual energy, communication reliability, queuing congestion, proximity to sink and quality of links. Therefore, independent trajectory and routing decisions were not made: the chosen sink movement changed the following topology and communication conditions, and the routing decision changed energy distribution, congestion and packet delivery performance for the following state.

(16) $$a_{t}=\left(m_{t},r_{t}\right)\in A_{t}$$

(17) $$A_{t}=A^{M}\times A_{t}^{R}$$

where

(18) $$A^{M}=\left\{N,S,E,W,\mathrm{NE},\mathrm{NW},\mathrm{SE},\mathrm{SW},\mathrm{Stay},\;\mathrm{Hotspot}\right\}$$

And

(19) $$A_{t}^{H}=\left\{j:j\in N_{i}\left(t\right),j\;\mathrm{is}\;\mathrm{an}\;\mathrm{eligible}\;\mathrm{forward}\right\}$$

Here, *N_i_*(*t*) represents the dynamically available neighboring sensor nodes of transmitting node *i* at communication round *t*. This is consistent with the manuscript’s routing mechanism, which prioritizes forwarding nodes according to residual energy, queue condition, communication reliability, graph attention, neighborhood density, and proximity to the optimized sink trajectory.

Equation (20) formed the observation state used by the reinforcement learning agent.

(20) $$O_{t}=\left[Z_{G}∥M_{t}∥C_{t}∥{\bar{E}}_{t}∥{\bar{D}}_{t}\right]$$

where *O_t_* represented the observation state at time *t*, *Z_G_* denoted the graph-level network embedding, *M_t_* represented the mobile sink state vector, *C_t_* denoted the average congestion state, ${\bar{E}}_{t}$ represented the average residual energy of active nodes, ${\bar{D}}_{t}$ denoted the average transmission delay, and ∥ represented feature concatenation.

An experience replay memory, where the number of communication transitions to be stored in memory is 100,000, was added for stabilizing reinforcement learning. In training, all the elements of the communication experience were constantly stored in the replay buffer. To avoid temporal correlation between consecutive experiences, mini-batches of 256 transitions were randomly sampled, for each iteration in the optimization process. An offline learning network was also kept in parallel with the online learning network, and every 500 optimization cycles the parameters were synchronized. The target update resulted in a significant delay reduction in oscillatory behavior when learning policies and a better stability of convergence when learning in highly dynamic wireless communication environments. The exploration policy was designed to be “epsilon-greedy” with a high initial value (1.0) of the probability of exploring, which was gradually reduced to 0.05 to progressively shift the reinforcement learning agent from exploring to exploiting learned routing strategies.

Equation (21) estimated the expected value of every trajectory and routing action.

(21) $$Q\left(O_{t},a_{t};\theta \right)=f_{\theta }\left(O_{t},a_{t}\right)$$

where *Q*(*O_t_*, *a_t_*; *θ*) represented the predicted Q-value for observation *O_t_* and selected action *a_t_*, *θ* denoted trainable DQN parameters, and *f_θ_* represented the deep neural network used to approximate the action-value function.

One of the main novelties of the proposed TrajGNN-RL framework was its ability to generate rewards. The proposed reward formulation was designed to optimize network’s energy usage and lifetime, as well as other metrics, including residual energy balance, packet delivery success, throughput, communication delay, routing overhead, sink traveling efficiency, congestion reduction, forwarding fairness and communication reliability. The positive rewards given were for a decrease in the average communication distance, an equal or balanced forwarder load, an increase in the packet delivery ratio and/or the conservation of battery energy to multiple sensor nodes for sink movements that reduced average communication distance. When the routing caused the communication delay to be too high, data loss or frequently selecting an overloaded node for forwarding, or causing unnecessary movements of sinks (which increased traveling distance without having an impact on data collection), negative rewards were introduced. This reward strategy balanced rewards to avoid the learning agent giving preference to one performance objective while giving a deteriorating value to other important network characteristics.

Equation (22) computed the reward used to guide joint trajectory and routing optimization.

(22) $$R_{t}=\lambda_{1}\mathrm{PD}R_{t}+\lambda_{2}EE_{T}+\lambda_{3}T_{t}-\lambda_{4}D_{t}-\lambda_{5}OH_{t}-\lambda_{6}TD_{t}$$

where *R_t_* represented the reward at time *t*, *PDR_t_* denoted packet delivery ratio, *EE_t_* represented energy efficiency, *T_t_* denoted throughput, *D_t_* represented average delay, *OH_t_* denoted routing overhead, *TD_t_* represented sink traveling distance, and *λ*_1_ to *λ*_6_ represented reward balancing coefficients used to control the importance of each performance objective. The suggested coefficient values can thus be given as 0.25 for PDR, 0.25 for energy efficiency, 0.20 for throughput, 0.15 for delay penalty, 0.10 for routing-overhead penalty, and 0.05 for sink-travel penalty. The weighting coefficients were assigned based on the relative importance of the competing objectives in the mobile sink routing in the context of lifetime. For PDR (λ_1_ = 0.25) and energy efficiency (λ_2_ = 0.25), the highest weights are assigned, as they directly contribute to the sustainability of the network operation with a reliable packet collection, while reducing the amount of energy needed to be consumed by the sensors. To ensure efficient data transfer, while not letting the amount of data overwhelm the learning goal, throughput was set at λ_3_ = 0.20. To ensure that most packets are delivered within the minimum time, the penalty weight of delay (λ_4_ = 0.15) was set to be larger than the other penalty weights, while routing overhead (λ_5_ = 0.10) and sink-travel distance (λ_6_ = 0.05) were assigned to be smaller. All the coefficients are fixed at 1.0 for a normalized trade-off between the six objectives. Some alternative emphasis configurations were investigated using the reward-weight settings listed in Table 2, such as delivery-based, energy-based, throughput-based and equalized settings. The chosen “balanced” configuration was kept to avoid the policy making an overly strong choice for any particular metric at the cost of the network’s operation. Unlike existing work on routing based on user-defined forwarding rules and/or heuristic optimization of the fixed reward, this reward formulation continuously provides multi-objective feedback to the DRL agent and lets the routing and sink movements evolve together depending on the actual network state encoded in the graph.

The joint decision at communication round *t* is defined as:

(23) $$a_{t}=\left(a_{t}^{M},a_{t}^{R}\right)$$

where $a_{t}^{M}$ denotes the mobile sink-movement action and $a_{t}^{R}$ denotes the packet-routing action. The sink-movement set consists of the following 10 actions: north, south, east, west, northeast, northwest, southeast, southwest, stationary collection, and movement towards the predicted traffic hotspot. The routing action determines the next sensor to forward the message based on the next sensor’s residual energy and the reliability of the wireless link, as well as the level of forwarding congestion. Thus,

(24) $$a_{t}^{R}=\arg\underset{j\in N_{i}}{\max}\left(w_{1}E_{j}+w_{2}L_{j}-w_{3}C_{j}\right)$$

where *E_j_*, *L_j_* and *C_j_* denote normalized residual energy, link reliability, and congestion of candidate node *j*, respectively. This formalizes the routing behavior already described in the framework.

The GNN generates topology-aware embeddings $Z_{t}=f_{\theta_{G}}\left(G_{t}\right)$, which are concatenated with the mobile sink and network states to construct the DQN observation *s_t_*. The GNN is trained using a topology-preservation reconstruction loss,

(25) $$L_{\mathrm{GNN}}=-\sum_{\left(i,j\right)}\left[A_{\mathrm{ij}}\log{\hat{A}}_{\mathrm{ij}}+\left(1-A_{\mathrm{ij}}\right)\log\left(1-{\hat{A}}_{\mathrm{ij}}\right)\right]$$

While the DQN minimizes the temporal-difference loss

(26) $$L_{\mathrm{DQN}}={\left[r_{t}+\gamma \underset{a^{\prime }}{\max}Q_{\theta }-\left(s_{t+1},a^{\prime }\right)-Q_{\theta }\left(s_{t},a_{t}\right)\right]}^{2}$$

Each time a message is communicated, the GNN generates the graph embedding, and the DQN then chooses the sink movement and routing decision. The resulting reward and next network state are put in replay memory. Next, 256 transitions are sampled from mini-batches to update the DQN, and the updated energy, queue, link, traffic and sink information is utilized to reconstruct the communication graph, which is fed into the next GNN embedding. The DQN is synced with the target DQN every 500 optimization updates.

After each communication round, the communication graph was dynamically reconstructed from the current set of sensors’ information after the packet transmission. The calculated residual battery levels, forwarding frequencies, sink position and the level of occupancy of the queues, the traffic density and the communication statistics were added to the graph representation immediately. The graph neural network then computed new node embeddings based on the new network state, which were then fed to the reinforcement learning agent for the next decision cycle. This was a kind of a feedback loop, where graph intelligence evolved together with reinforcement learning policy during the network operation. In contrast to the static nature of the routing protocols used in traditional architectures, the proposed hybrid learning architecture could continuously improve its decision-making capability based on the changing communication conditions; this enabled sink trajectories and routing policies to be improved by an incremental process over time while the network is in operation.

A total of 1500 training episodes of 500 rounds of communication were run for reaching policy convergence, randomly initialized sink locations and different traffic scenarios. To increase the policy generalization over various deployment environments, random sink velocities ranging from 1 m/s to 10 m/s, ranging of the communication distance from 40 m to 60 m and generation rate of packet (2 to 10 packets per second), were added during training. Model checkpointing was used to maintain the best performing policy based on the validation reward, and learning curves were monitored for the stability of the learning process and to assure non-overfitting. By relying on such a tight coupling between graph learning and reinforcement learning, the proposed TrajGNN-RL framework can continuously adapt to optimal routing and trajectory strategies in highly dynamic wireless sensor network (WSN) environment while achieving uniform energy consumption, minimum communication delay, and maximizing network life.

**Figure 5:**
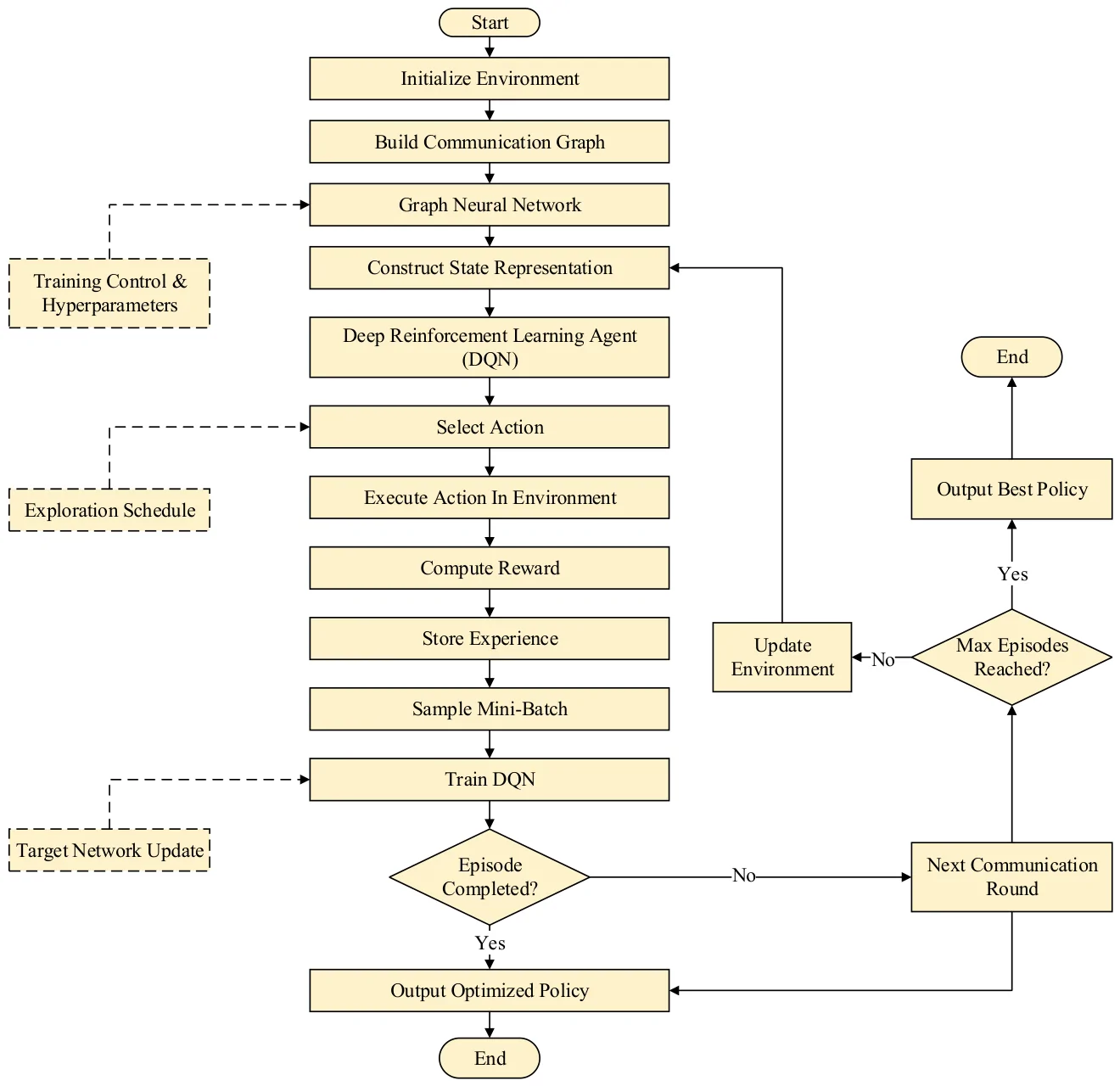
Flowchart of the proposed TrajGNN-RL hybrid learning framework.

To combine the advantages of both approaches, Algorithm 1 introduced the whole TrajGNN-RL hybrid learning framework, which is a hybrid of the topology learning strategy from GNN and the optimization strategy from deep reinforcement learning (DRL). First, a communication graph of dynamic wireless sensor network was generated, which was described by the graph embeddings. The information from these embeddings was then used in conjunction with the information from the mobile sink to build the observation state that was provided to the reinforcement learning agent. In each of the training episodes, the actions selected for each route were standardized, the communication rewards were calculated and updated replay memory was updated with new communication experiences observed. Graph reconstruction over time led to adaptively refining the graph embeddings and routing policies that, in turn, allowed to optimize sink trajectory planning and packet forwarding while operating the network. Figure 5 depicts the proposed TrajGNN-RL hybrid learning framework for intelligent mobile sink routing as follows: graph learning, reinforcement learning, adaptive decision-making, feedback loops, episode-based policy optimization, and trajectory refinement.
**Algorithm 1:** TrajGNN-RL Hybrid Learning Framework**Input:** Communication graph G, mobile sink state *M* **Output:** Optimized routing policy *π*
1: Initialize graph neural network 2: Initialize deep reinforcement learning agent 3: Initialize replay memory 4: For each training episode do 5:      Generate graph embeddings 6:      Observe current network state 7:      Select sink-movement action 8:      Execute routing decision 9:      Calculate reward 10:    Store transition in replay memory 11:    If replay memory is sufficient then 12:        Update reinforcement learning policy 13:        Update graph neural network parameters 14:    Else 15:        Continue exploration 16:    End if 17:    Reconstruct communication graph 18: End for 19: Return optimized policy

### 3.5. Adaptive Deep Reinforcement Learning-Based Mobile Sink Trajectory Optimization

After generating graph embeddings and the initialization of the hybrid learning architecture, the proposed TrajGNN-RL framework performed adaptive mobile sink trajectory optimization for wireless sensor network (WSN) environment via a deep reinforcement learning (DRL) agent which interacted with the wireless sensor network (WSN) environment continuously. In contrast to the traditional trajectory planning approaches based on a fixed movement pattern of the sinks, the proposed approach dynamically generated trajectories for the sinks based on the instantaneous communication state of the network. Decision of any movement was based on the above graph-level topology intelligence, communication reliability, node energy distribution, traffic density, forwarding workload and packet accumulation, which allowed the mobile sink to intelligently move itself based on changes in network conditions. As a result, the mobility was adapted, and now it is not an established navigation scheme, but rather a process of optimization, which greatly enhanced communication efficiency during an extended operation of the network.

Equation (27) selected the optimal mobile sink-movement action during every decision interval.

(27) $$a_{t}^{*}=\arg\underset{a}{\max}Q\left(O_{t},a;\theta \right)$$

where $a_{t}^{*}$ represented the optimal action selected at time *t*, *Q*(*O_t_*, *a*; *θ*) denoted the estimated action-value function, *O_t_* represented the observation state, *a* denoted the candidate movement action, and *θ* represented the trained Deep Q-Network parameters.

The reinforcement learning environment was described as a sequential decision-making process involving the mobile sink, which always observed the current network condition and made decisions on what to do to move to the next movement for the mobile sink. Whenever they communicated, the 256 dimensional graph embeddings produced by graph neural network were included in the observation space, as well as the sink’s position, velocity, direction of travel, distance traveled, residual energies of the nodes, number of packets in queue, number of packets forwarded, density of the neighboring communication area, frequency of packet generation, average delay for a communication, probability of successful packet delivery, and statistics of congestion in the region. This was a multi-dimensional observation space, which allowed the reinforcement learning agent to get a full picture of the local communication and global network. The proposed observation mechanism enabled decisions on the trajectories to be based on the overall health of the wireless sensor network whereas traditional routing methods only used the health of the nearest sensor nodes to determine the routing.

While designing the action space intentionally to have enough flexibility for intelligent action for sink navigation, efficient computation of the policy optimization during the action phase was kept in mind. Each communication round, the reinforcement learning agent (RLA) selected from 10 actions one to take, one of which was to move towards the north, south, east or west, one of which was to move towards the northeast or northwest, one of which was to move towards the southeast or southwest, one of which was to temporarily stop and receive information, and one of which was to move to a predicted communication hotspot. The framework proposed had one unique aspect to it, which was the adaptive hotspot action. The set of regions where the traffic would be piling up in the future was dynamically learned by the sink using graph embedding, and the sink proactively moved towards the region that would have a higher traffic demand for communication in the next time period. This predictive mobility helped to decrease packet buffering time and the average communication path in the network before network congestion increased significantly.

A learning environment with the trajectory refinement constraint was used to prevent unneeded oscillating of the sink and to solve the problem of the learning behavior. The coloring of the transitions between adjacent sinks based on their packet collection was negatively reinforced if they were “sinking” in and out of adjacent sinks without resulting in an increase in packet collection and were positively reinforced if they were moving over a larger sensing area. Furthermore, the distance the user walked in the movement actions which did not result in an improvement of the throughput or energy balance was penalized during the policy learning. The refinement strategy allowed the mobile sink to develop stable navigation patterns and perform navigation in an efficient manner without making substantial short-range refinements which would lead to higher communication overhead.

Equation (28) updated the geographical position of the mobile sink after every movement decision.

(28) $$P_{t}^{\mathrm{new}}=P_{t}^{\mathrm{old}}+V_{t}\Delta t\;$$

where $P_{t}^{\mathrm{new}}$ represented the updated sink position, $P_{t}^{\mathrm{old}}$ denoted the previous sink position, *V_t_* represented the sink velocity vector, and Δ*t* represented the movement interval.

The Deep Q-Network with three hidden fully connected layers with 512, 256 and 128 neurons, respectively, was used to optimize the reinforcement learning policy. The activation functions used in all the hidden layers were ReLU, and linear activation was used at the output layer to estimate the action values related to the actions of candidate sink movements. With Adam optimization algorithm, the initial learning rate was set to 0.0005, the learning rate was then decreased exponentially after each 200 training episodes, and the network parameters were updated. A discount factor of 0.95 was chosen to give more importance to enhancing long-term network lifetime rather than to collecting more packets within a single time frame, which helped to choose strategies for moving around that preserved the remaining battery energy in the sensing field. We used mini-batches of 256 randomly sampled transitions, and experience replay memory consisting of 100,000 interaction samples was used in each iteration of optimization, thus removing temporal correlation between each interaction. Network synchronization was carried out on targets every 500 optimization updates to preserve the stability of learning and to avoid the oscillation of the convergence of the parameters under the dynatopics communication condition.

For the TrajGNN-RL action space it was kept tractable by not using a global action space in which the DQN had to explicitly choose from among all possible routing decisions for each sensor node. The number of movement actions in the action space of the DQN was kept at the same 10 discrete movements, irrespective of the number of sensor nodes in the network (namely 100, 200, or 300). The forwarding of packets was performed locally with the dynamically available adjacent nodes instead of building up a routing action space for the entire network. The GNN encoded the variable size communication topology into a fixed dimensional graph representation, and the candidate forwarding nodes were evaluated based on residual energy, queue occupancy, communication reliability, link quality, neighborhood information and proximity to sink. As a result, making more of the nodes active increased the size of the graph but not proportionally the DQN output dimension for movement. The routing complexity mainly relied on the neighborhood size of a transmitting node, instead of the network size. This structured decomposition, by contrast, made it possible to avoid a combinatorial growth of the action space for the joint correspondence between trajectory and routing and enabled an evaluation of the considered 100–300 node configurations.

An extensive reward estimation framework was designed, which optimizes multiple performance objectives of a wireless sensor network (WSN), rather than just a single optimization criterion. For each type of sink-movement reduction in the average transmission distance, increased PDR, balanced residual energy consumption, minimized communication delay, improved throughput, and decreased forwarding congestion resulted in positive rewards. Further positive reinforcement was given if the sink could reach areas that had high numbers of packets in them before the queues overflowed, causing packet loss—specifically when the sink reached these areas before the queues became full of packets. Negative rewards were added if the movements resulted in an increased communication delay, caused path congestion, frequently chose already congested sensing regions or caused a faster battery consumption within the forwarding nodes. In addition, the excessive travel time to the sinks, without any improvements in network performance, was sanctioned to promote efficient mobility and not continuous exploring. Overall, for this multi-objective reward strategy, this provided an optimal trajectory to make the communication efficient, conserve energy, be fair and keep the network alive.

Equation (29) evaluated the routing cost for every candidate forwarding node.

(29) $$RC_{\mathrm{ij}}=\frac{d_{\mathrm{ij}}\times Q_{i}}{E_{j}\times L_{\mathrm{ij}}}$$

where *RC_ij_* represented the routing cost from node *i* to node *j*, *d_ij_* denoted the communication distance, *Q_i_* represented the packet queue length of node *i*, *E_j_* represented the residual energy of forwarding node *j*, and *L_ij_* denoted the communication link quality.

The wireless sensor network environment was constantly changing throughout policy optimization, to follow the changing behavior of the communication. The residual battery energy was reduced after each transmit, the amount of work to complete each forward was different depending on the routing, the packet queues fluctuated depending on the amount of sensing activity and the wireless communication links were changing due to the movement of the sink. The proposed framework did not make any assumption of network conditions, but instead it could build a new graph of communication after each transmission cycle based on the most up-to-date node information. The next observation state to be presented to the reinforcement learning agent was then immediately embedded into a graph and the updated graph was used. All decisions on sink movements were based on the most recent communication conditions, rather than past observations, enabling trajectory optimization to be very responsive to changes in wireless conditions.

An adaptive exploration mechanism was added to further increase the robustness of learning in a long-term training time. The reinforcement learning agent was initially given a high chance of exploration (μ = 1.0) and allowed to explore a variety of strategies for moving the sink before convergence of policies to experience many communication scenarios and then converge to a policy. As training continued, exploration probability was slowly decreased using an exponential decay schedule to 0.05 to promote exploitation of already learnt movement strategies, whilst allowing a certain amount of exploration to ensure it would be able to adapt to unexpected network conditions. To enhance the ability of the policies to transfer to a variety of deployment scenarios, a variety of randomized sink initializations, node densities, communication ranges, packet generation rates (2–10 packets per second), and sink velocities (1–10 m per s) were added to every training episode. A total of 1500 episodes (comprising 500 communication rounds) were carried out until the sum of rewards approached convergence and the results of the validation performance reached a stable state.

One of the main innovations of the proposed TrajGNN-RL framework was that it continuously interacted between the trajectory optimization and graph representation learning process. To optimize the movement of the sinks we did not optimize them separately, but rather each decision about the movement of the sink had an effect on the topology of the graph, the reliability of the communication, the connectivity of the neighborhood, and the forwarding behavior, which in turn affected the graph embeddings fed to the reinforcement learning agent. This bi-directional feedback allowed the sink to learn the movement strategies that continually modified the moving strategy in response to this changing communication situation instead of using fixed movement patterns. Therefore, the optimized trajectories not only reduced transmission delay and packet loss, but also evenly spread out the communication workload among the sensing field, avoiding the occurrence of energy hole, and the lifetime of wireless sensor network in the highly dynamic sink mobility scenarios was also significantly prolonged.

As described in Algorithm 2, the adaptive mobile sink trajectory optimization procedure was conducted using the reinforcement learning policy trained by the proposed TrajGNN-RL framework. The reinforcement learning agent used to inspect the graph embeddings, communication quality, location of the sink and the network conditions in each communication interval to choose the most suitable sink-movement direction. When the prediction of sink position resulted in an improved communication efficiency, it was accepted; otherwise, an alternative type of movement was chosen. After each move, they collected packets and rebuilt the graph to keep up to date with the communication environment. This continual process allowed adaptive sink mobility, shortened communication distance, load balance of forwarding among network nodes and enhanced the performance of the network in the dynamic wireless sensor network environment.
**Algorithm 2:** Adaptive Mobile Sink Trajectory Optimization**Input:** Graph embedding *ZG*, observation state *Ot*, sink position *Pt* **Output:** Optimized sink trajectory *T*
1: Initialize sink position *Pt* 2: Initialize trajectory *T* 3: For each communication round do 4:      Receive graph embedding *ZG* 5:      Construct observation state *Ot* 6:      Select movement action using routing policy 7:      Predict next sink position 8:      If communication quality improves then 9:             Move sink to predicted position 10:        Store updated position in *T* 11:    Else 12:        Select alternative movement direction 13:        Update sink position 14:    End if 15:    Collect sensed packets 16:    Update communication graph 17:    Calculate communication reward 18: End for 19: Return optimized trajectory *T*

### 3.6. Intelligent Energy-Balanced Routing and Continuous Graph-Reinforcement Policy Adaptation

After optimizing the trajectory of the mobile sink, the proposed TrajGNN-RL framework adopted an intelligent routing policy that dynamically adjusted routing based on the residual battery power as well as applied deep reinforcement learning (DRL) and GNN learning, for continuously improving the routing policy. With the proposed framework, instead of re-calculating the shortest paths or choosing the nodes to forward the packets periodically as done by the conventional energy-aware routing protocols, all of the transmission paths were established before they were based on the communication reliability, graph topology evolution, forwarding congestion, sink proximity, energy distribution in the neighborhood, communication traffic density, and historical routing behaviors. Dynamic routing was provided, without making the routing decision frozen after the first route discovery, but changing in time, depending on the new wireless sensor network environment. The significant gain of this adaptive routing method is that it greatly enhances the stability of communication, and not only does it avoid the premature exhaustion of nodes with high traffic load along the routes, but it also avoids the installation of redundant honeypot nodes.

All the sensor nodes that were active in the network assessed the operating conditions at their own level in the beginning of each communication interval and sent the network statistics to the graph construction module. The new data consisted of residual battery power, packet queue occupancy, the ratio of transmissions that were successful, the ratio of forwardees, average communication delay, the number of times that packets were retransmitted, Received Signal Strength Indicator (RSSI), Link Quality Indicator (LQI), neighborhood communication degree, sink distance, and channel utilization. These measurements were instantaneously fed into the reconstructed communication graph after each transmission cycle. This updated graph was then fed into the graph neural network for refining the node embeddings of the wireless sensor network, with the refined embeddings representing the current structure of the sensor network, based on the previously learned parameters of the network. The proposed framework continuously regenerated the graph intelligence after each communication round, whereas static routing algorithms were always based on the topology information that they used, which was outdated.

(30) $$\mathrm{EBI}=\frac{1}{1+\frac{\sigma_{E}}{\mu_{E}+\varepsilon }}\;\mathrm{with}\;0<\mathrm{EBI}\leq 1\;$$

An index that is bounded in energy balancing is given by Equation (36). As the *EBI* gets closer to one, the energy dispersion of the other nodes is more and more uniform, and as the energy dispersion increases, the index approaches one.

The adaptive routing module then considered a set of forwarding nodes and selected the best based on both graph embeddings and statistics on such communications. Based on the above, every neighboring node was given a priority score to forward the packet with respect to residual battery energy, queue length, communication reliability, frequency of packet forwarding, average success in delivering the packets, graph attention score, neighborhood density and closeness to optimized sink trajectory. More reliable communication nodes with a moderate amount of battery consumption were given higher forwarding priority, while battery overloaded relay nodes, with higher traffic volume and/or rapid battery loss, were assigned decreasing forwarding priority. This smart forwarding scheme avoided the use of the same communication paths and evenly spread the job of packet forwarding across the sensing field. As a result, the energy consumption in the nodes was kept balanced, which significantly reduced energy depletion and partition of the network in the local nodes.

A dynamic load-balancing mechanism monitored the whole wireless sensor network’s forwarding activity continuously for further enhancing the stability of routing. Cumulative forwarding counts for each sensor node were computed and compared to the workloads in neighboring transmissions during each transmission interval. If the imbalance of the forwarding was above a certain threshold (set to be 15% in the experiments presented), the routing policy automatically re-routed traffic to neighboring sensor nodes that had enough battery energy and good communication options left, to relieve the imbalance. Since the forwarding nodes ran out of energy before a significant energy imbalance occurred, proactive load redistribution was done before the severe energy imbalance occurred. This balancing approach predicted which trajectory to take made it possible to avoid the formation of energy holes in the vicinity of mobile sink trajectories as it is often the case with conventional routing protocols. At the same time, the packet queues were constantly checked and when a temporary traffic peak was caused by concentrated sensing activity, the necessary modification to traffic was made to avoid communication bottlenecks.

Equation (31) updated the reinforcement learning parameters during policy optimization.

(31) $$\theta^{\mathrm{new}}=\theta^{\mathrm{old}}-\eta \nabla_{\theta }L\left(\theta \right)$$

where *θ^new^* represented the updated network parameters, *θ^old^* denoted the previous parameter values, η represented the learning rate, ∇*_θ_* denoted the gradient with respect to the network parameters, and *L*(*θ*) represented the optimization loss.

To alleviate the problem of unreliable wireless links, an adaptive communication reliability estimator was added to make a better route selection decision. Instead of taking communication quality as a constant while the network is operating, the estimator took sample readings after every packet sent to analyze the success rate of packet delivery, rate of re-transmissions, the variation in RSSI, the fluctuation in LQI, the delay in communication and the amount of the neighboring interference after each packet sent. The communication stability of wireless links was gradually decreased in assigning progressively lower routing confidence values while the link was assigned with higher routing preference value during subsequent forwarding decisions if it provided more stable wireless communication. This feedback-based communication evaluation allowed the routing framework to automatically steer clear of getting into harmful wireless links, prior to substantial packet losses. As a result, the overall transmission reliability was increased, and unnecessary retransmissions were decreased, which led to accelerated battery consumption all over the sensing field.

The reinforcement learning policy learned routing information based on the performance of the routings after each communication cycle. Fresh reward information was created for each episode of the route for the following reasons: packet delivery ratio, throughput, communication delay, remaining battery balance, routing efficiency towards the sink, forwarding fairness, routing overhead, and packet collection success. A set of performance indicators was recorded as communication experiences in the replay memory along with the graph embedding, the routing actions chosen by the agent, the environment state which followed the communication, and the terminal state of the environment. A set of randomly sampled experience batches of size 256 were used to continuously tune the parameters of Deep Q-Network with the Adam optimization algorithm. The parameters of the target network were updated every 500 optimization steps, which provided a stable convergence of the reinforcement learning and avoided the oscillation of network parameters when the communication environment was very dynamic, without compromising the target network’s performance. This never-ending process of improving the policy allowed for progressive improvement of routing intelligence over time during extended network operation.

Equation (32) synchronized the target network with the online learning network.

(32) $$\theta^{-}=\theta$$

where *θ*^−^ represented the target network parameters and *θ* denoted the updated online network parameters transferred after the synchronization interval.

The bidirectional feedback mechanism set up between the graph representation learning and reinforcement learning (RL) policy optimization was among the main innovations that were introduced by the proposed TrajGNN-RL framework. In contrast to existing routing algorithms where topology learning and routing decisions were made independently, with every routing action new information about communication was added, which was immediately applied for the reconstruction of the graph during the next communication period. The observation space fed to the reinforcement learning agent was then updated based on the newly made graph embedding and the agent made better decisions for routing and trajectory for the next communication round. This ongoing interaction has also allowed graph intelligence and routing intelligence to develop concurrently and it enables the framework to identify new congestion areas, loss of communications, battery depletion patterns and opportunities to move sinks much before conventional adaptive routing algorithms.

The communication scenarios were randomly chosen to better cope with the robustness of different deployment scenarios. The number of sensor nodes in the network varied from 100 to 300, the velocity of the sink nodes ranged from 1 to 10 m/s, and the frequency of generation of packets was 2 to 10 packets per second. The communication distance was set as 50 m, and a separate sensitivity study was performed to change the communication distance from 40 m to 60 m. This change was made solely for the purpose of testing the robustness of TrajGNN-RL when network connectivity changes and was not the standard mode of communication. In the primary comparative experiments, the communication radius was set at 50 m for both the proposed method TrajGNN-RL and the baseline methods to provide the same communication radius to allow a consistent and fair evaluation.

**Figure 6:**
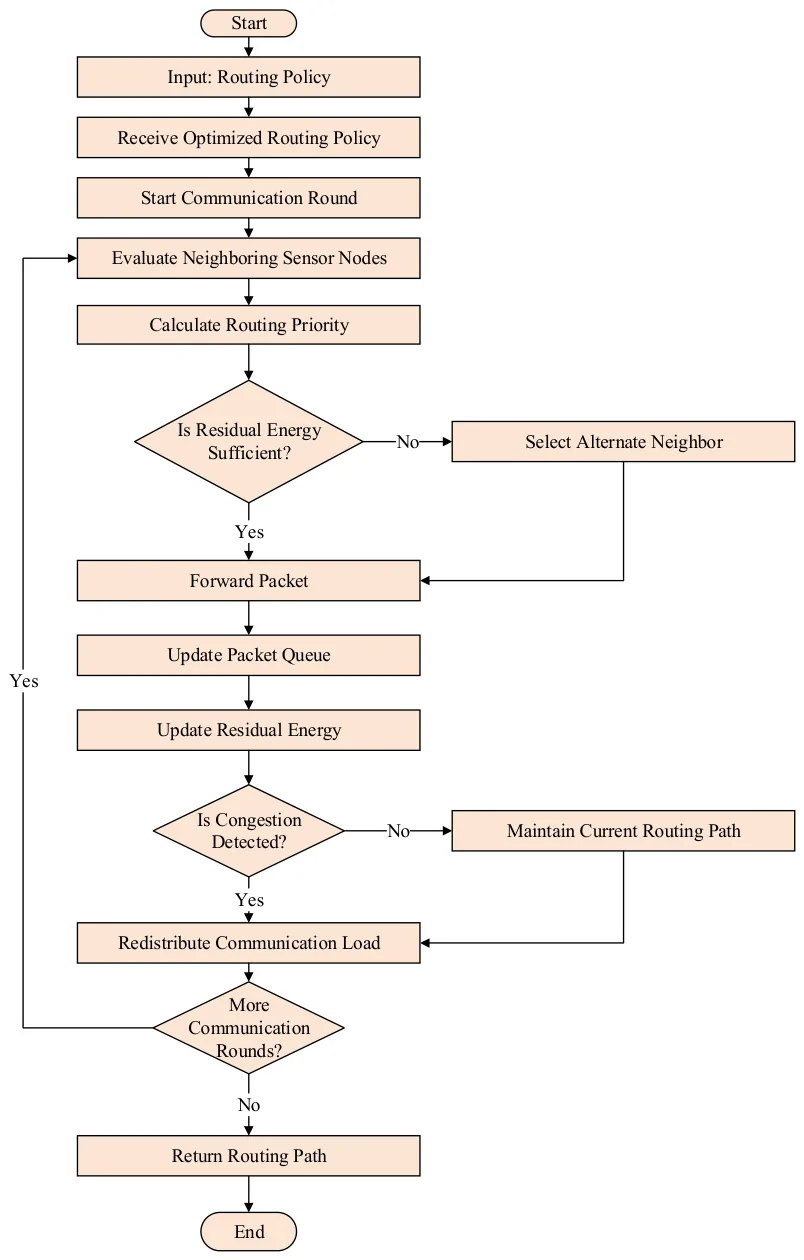
Flowchart of the adaptive mobile sink routing algorithm.

Finally, to confirm the stability of the policy, convergence monitoring was carried out continuously during hybrid learning and the training was terminated when the policy converged. The training was finished when the reward improvement was less than one percent in the history of 20 consecutive validation episodes, while maintaining the PDR, RES, CD and NL to converge in a stable manner. The network parameters with the best performance on the validation reward set were retained by training only on this reward and model checkpointing to avoid overfitting on this reward instead of the training reward. By combining the learning ability of graph neural network and optimization ability of deep reinforcement learning, the resulting routing policy was a highly adaptive intelligence model that can continuously adapt to the communication load, ensure residual battery energy, reduce routing overhead, limit the delay of the packets, and prolong the lifetime of the wireless sensor network. Figure 6 shows the adaptive mobile sink routing process, which consists of neighbor evaluation, neighbor’s routing priority calculation, energy-aware forwarding, congestion control, iterative rounds of communication and balanced routing path generation.

### 3.7. Experimental Configuration and Performance Evaluation

The proposed TrajGNN-RL framework was experimentally evaluated by conducting extensive simulations to assess the effectiveness of TrajGNN-RL under various deployment conditions of WSNs. The experiments were conducted in Python 3.11, deep learning framework PyTorch 2.2, and PyTorch Geometric (PyG) library for implementing graph-based neural network. The simulation of the network was done with the help of NetworkX library, and the numerical calculations and data preparation with NumPy, Pandas and Scikit-learn libraries. It was run on a workstation with an Intel Core i7-13700K CPU, 32 GB DDR5 RAM and NVIDIA RTX 4070 GPU (with 12 GB of dedicated memory) on Ubuntu 22.04 LTS. The learning time for graphs was drastically reduced by the use of the GPU during multiple policy optimizations, and the CPU was responsible for data preprocessing, communication event scheduling and handling network simulation. Experimental parameters were chosen to cover a wide range of WSN operating conditions, and learnability was ensured, with similar assessment conditions. In the experiments, the operating point of the network is not limited but varied with the number of sensors, the packet generation rate, and the velocity of the mobile sink. The learning configuration was decided based on the performance of the validation and stability of the convergence in the preliminary process of developing the model. When learning went through a stable reward and validation behavior without significant oscillations or overfitting, the GNN depth, hidden dimension, embedding size, attention configuration, learning rates, replay-memory capacity, batch size, discount factor, target-network update interval, and exploration schedule were kept. Subsequently, various operating scenarios were investigated by playing with different levels of sensor-node density, traffic intensities, different mobilities of the sinks, and different communication conditions, to test the robustness of the chosen configuration. No comprehensive hyperparameter search over different possible combinations of the GNN and DRL was used, however, and the values used should be considered as stable parameters found through experimentation rather than as global optimum parameters. Further automatic hyperparameter optimization can be used to gain further improvements in performance.

Equation (33) estimated the normalized operational lifetime of the wireless sensor network.

(33) $$\mathrm{NL}=R_{\mathrm{FND}}$$

where *NL* denotes the operational network lifetime in communication rounds, and *R_FND_* is the communication round at which the first sensor node exhausts its energy. For normalized comparison,

(34) $$NL_{\mathrm{norm}}=\frac{R_{\mathrm{FND}}}{R_{\max}}\times 100$$

where *R_max_* is the maximum evaluated communication-round horizon. The percentage of nodes remaining active at round *r* is separately defined as the node survival ratio.

(35) $$\mathrm{SR}\left(r\right)=\frac{N_{\mathrm{alive}}\left(r\right)}{N_{\mathrm{total}}}\times 100$$

Thus, network lifetime is expressed in communication rounds, whereas node survival is expressed as a percentage.

The packet delivery ratio was defined as the percentage of packets that were successfully received to the number of packets that were sent. Throughput was the amount of successfully transmitted data for each communication period. Energy efficiency was defined as successfully delivered packets per Joule of communication energy. The end-to-end delay was measured to be the time elapsed between the generation of a packet and reception at the sink, measured in milliseconds. The ratio of control packets to the total number of packets transmitted was called routing overhead. Delay reduction was defined as the difference between the delay and the baseline delay, and node survival ratio was defined as the number of sensor nodes operating at a given communication round over the number of sensor nodes that are operational at the baseline.

The experimental wireless sensor network consisted of 0.1, 0.15, 0.2, 0.25 and 0.3 densities (number of sensor nodes per unit area (1000 m^2^)) of 100, 150, 200, 250 and 300 nodes respectively, randomly deployed in a 1000 m × 1000 m sensing area. Initially, all sensor nodes had 2 Joules of battery power, a communication range of 50 m, a sensing range of 25 m and they produced data packets of 512 bytes with varying rates of 2 to 10 packets per second. The velocity that the mobile sink traversed the sensing field ranged between 1 m/s to 10 m/s to represent high-speed mobile data collection applications and low-speed environmental monitoring. When direct sink communication was not available, communication was done with multi-hop forwarding and IEEE 802.15.4 wireless communication standard. The episodes’ length for simulation was set at 500 communication rounds, and 1500 independent learning episodes were conducted to guarantee that the reinforcement learning policy converged with complete convergence. To remove the stochastic variations due to the random deployment of nodes and communication behavior, we repeated each experimental configuration 30 times independently with different random initializations of the network. The average performance values were reported for each experimental configuration, eliminating stochastic variations due to random node deployment and communication behavior.

The graph neural network used in the TrajGNN-RL consisted of three graph convolution layers, one graph attention refinement layer and one embedding projection layer, resulting in 256-dimensional node embeddings. The size of hidden layers was set to 128 neurons, and the graph attention mechanism was set to use eight parallel attention heads to receive information from multiple communication perspectives in the neighborhood. The deep reinforcement learning was a Deep Q-Network with fully connected hidden layers of 512, 256 and 128 neurons, respectively. The Adam optimizer with learning rates of 0.001 and 0.0005 for graph learning and reinforcement learning optimization, respectively, was used for training. Replay-memory size was kept at 100,000 communication transitions, mini-batch size was fixed to 256, the discount factor was set to 0.95, and synchronization of the target network was done every 500 optimization iterations. The exploration probability (epsilon) was reduced to 0.05 from 1.0, and the parameter updates were not allowed to be too large by using gradient clipping at 1.0, throughout the training process of the policy. To prevent unnecessary computation after convergence, the following early stopping method was used when learning graphs with a patience of 15 epochs of validation were employed.

To validate the proposed framework, TrajGNN-RL was compared with a number of representative mobile sink routing and energy-aware communication methods, with the same simulated scenarios. There were comparative methods such as LEACH-Mobile, PEGASIS, HEED, EEUC, ACO-Mobile Sink Routing, PSO-Based Sink Mobility, Deep Q-Routing (DQR) and Graph Convolution Routing (GCR). To fairly compare the performance of all the competing algorithms, the same number of sensor nodes, packet generation rate, sink mobility range, communication parameters and simulation time were used in all the algorithms.

Equation (36) computed the overall performance score of the proposed TrajGNN-RL framework.

(36) $$\tilde{X}=\frac{X-X_{\min}}{X_{\max}-X_{\min}+\varepsilon }$$

Subject to

(37) $$\sum_{k=1}^{6}\lambda_{k}=1,\;\lambda_{k}\geq 0$$

Here, *L*, *EE*, *T* and *PDR* are benefit metrics, whereas delay *D* and routing overhead *RO* are cost metrics. Therefore, $1-\tilde{D}$ and $1-\tilde{\mathrm{RO}}$ are used so that lower delay and lower overhead increase the overall performance index.

All the heterogeneous measures are first normalized in the [0, 1] range independently before they are aggregated. Benefit measures add positively; cost measures are converted to complementary utility measures. This prevents the mixing of incompatible quantities and corrects the erroneous positive contribution to communication delays.

Performance evaluation was performed with respect to multiple complementary network quality indicators, to comprehensively evaluate the routing efficiency and communication intelligence. The percentage of communication rounds that were successfully completed until the first significant node energy loss (NNEL) was reached was used to measure the network lifetime. Network lifetime was defined as the percentage of communication rounds until the first significant node energy loss (NNEL). Energy efficiency was defined as the number of packets delivered successfully to the total amount of energy used for communications. The packet delivery ratio was used to measure the percentage of successful packets received at the mobile sink, as a measure of the delivery of the message that mobile sinks are receiving. The number of successful collections of information per communication interval was the measure of average throughput. Average end-to-end delay was measured as time to the packet to be received successfully by the sink after being transmitted. Other performance metrics analyzed are the routing overhead, average residual battery energy, load balancing index, traveling distance of the sink, communication success ratio and packet loss ratio. These indicators together allowed for a balanced assessment of the quality of the communication as well as long-term sustainability of network.

The end-to-end communication delay was defined as the time between sending a packet from a source sensor node to receiving it at the mobile sink. For *N_s_* successfully delivered packets, the average end-to-end delay was calculated as:

(38) $$D_{\mathrm{avg}}=\frac{1}{N_{s}}\sum_{i=1}^{N_{s}}\left(t_{i}^{\mathrm{recv}}-t_{i}^{\mathrm{gen}}\right)$$

where $t_{i}^{\mathrm{gen}}$ and $t_{i}^{\mathrm{recv}}$ represent the generation and successful reception times of packet *i*, respectively. The delay was reported in milliseconds. To quantify the improvement over a baseline routing scheme, delay reduction was calculated as:

(39) $$D_{\mathrm{red}}\left(\%\right)=\frac{D_{\mathrm{baseline}}-D_{\mathrm{proposed}}}{D_{\mathrm{baseline}}}\times 100$$

where *D_baseline_* represents the average end-to-end delay obtained using the corresponding baseline routing method and *D_proposed_* represents the average delay obtained using TrajGNN-RL. This formulation guarantees that the delay-reduction percent reported is a relative reduction and not an independent normalized delay performance score.

A statistical consistency was checked for every experimental evaluation by taking the average of the results obtained with all the independent simulation runs. To make sure that learning behavior and communication performance were stable, standard deviation was monitored for all performance measures during each performance. Validation learning curves showed that cumulative reinforcement learning reward converged after about 1250 learning episodes before training was finished, indicating that the learned policy had converged to an adaptive routing strategy. Likewise, graph embedding loss converged within approximately 120 epochs of optimization, which demonstrates the successful learning of the spatial features in the graph neural network. Convergence observations were performed to confirm that the proposed hybrid learning framework could learn a general route behavior instead of memorizing the communication scenarios.

The experimental study showed that iterative graph representation learning and reinforcement learning-based policy adaptation substantially improved the wireless sensor network performance when the sink moved dynamically. The adaptive trajectory optimization successfully minimized the unnecessary movement of the sinks while maintaining communication load across the various sinks that are used for the forwarding purpose. At the same time, it was possible to detect congestion regions, communication delays, and developing energy-hole formations at an early stage, which resulted in the avoidance of any significant degradation of the network. Therefore, the proposed TrajGNN-RL framework outperformed the traditional routing solutions in terms of network lifetime, energy utilization, throughput, packet delivery ratio and transmission delay. Thus, the topology intelligence-based approach using graph neural networks with DRL-based trajectory optimization could effectively build a highly adaptive communication framework that can operate efficiently and reliably in the mobile sink environment while maintaining the wireless sensor network’s operation under various deployments and changing mobile sink environments.

## 4. Result and Discussion

The experimental evaluation was re-structured in terms of the main research goals instead of offering several descriptive visualizations. The analysis was performed to understand if TrajGNN-RL increases energy sustainability, packet delivery, throughput, communication delay, congestion control, routing reliability, and node survival for the network under different network densities and sink mobility conditions. In Results, redundant information regarding the implementation settings, constructions of the graphs, reinforcement learning, and simulation parameters were removed, since this information is already given in Methodology. Where applicable, related figures were consolidated, and generic plots without any of the following types of evidence—comparative evidence, convergence evidence, sensitivity evidence, robustness evidence, or temporal evidence—were excluded. The remaining figures thus serve as concrete benchmark on assessing effectiveness, stability, scalability and lifetime-aware behavior of the proposed framework.

The TrajGNN-RL framework was developed as a simulation-based intelligent routing system, with the programming language Python 3.11 as the primary programming language. The entire implementation was designed and implemented in the Jupyter Notebook (v7.4.5). environment, which also provided an interactive environment to preprocess data, construct graphs, train models, optimize the models using reinforcement learning and visualize the results. The Pandas library was used to import and manage wireless sensor communication records and NumPy was used for numerical computation, matrix manipulation and normalization of features and statistical analysis during the implementation. The general process of generating the topology of the network, constructing the graph and managing the communication relationship was accomplished by employing the NetworkX library, which makes it possible to generate dynamic wireless sensor graph after each communication interval.

We have used PyTorch 2.2 and PyTorch Geometric (PyG) framework for implementing the graph neural network component. PyTorch Geometric modules are used to perform graph convolution operations, neighborhood message aggregation, graph attention learning and generating node embeddings. Deep reinforcement learning was implemented by utilizing the basic layers of PyTorch as follows: fully connected neural networks, replay-memory management, target network synchronization and policy optimization layers. The data preprocessing operations, such as Min–Max normalization, partitioning of the dataset, encoding the features, and evaluation of the performance, were performed by the aid of Scikit-learn. The visualization of topology evolution, the learning convergence, routing performance and communication statistics were done with Matplotlib.(v3.10.0).

The implementation used the Suthaharan Multi-Hop Wireless Sensor Network Dataset for communication, graph construction, and intelligent routing analysis. During preprocessing, incomplete samples and duplicate communication events were removed before subsequent feature processing and graph construction. About 70% of the processed communication records were given to the model training, 15% to the validation and the remaining 15% to the independent testing. All test results were collected from the structured holdout partition, in which records occurring after another in time were not used for model training or validation, as were the records used for topology partitions held out and unseen network scenarios. The graph neural network had three graph convolution layers, one graph attention layer and a 256-dimensional embedding layer. There were 128 hidden neurons in each graph convolution layer and eight attention heads in the graph attention mechanism to adaptively aggregate the neighborhood information. For enhancing the stability of learning and decreasing overfitting, the Rectified Linear Unit (ReLU) activation function, batch normalization and a dropout rate of 0.30 were added. Real-time operation is not a deployment of the real sensor hardware; instead, in the present study, it means adaptation online during the round. To reconstruct the communication graph, the current residual energy, the number of queued packets, link quality, forwarding activity, traffic conditions and the position of mobile sink were recorded at each round of communication. The GNN then computed new topology-aware embeddings, which are provided to the DQN for the next sink-movement and routing decision. During the network operation, the network-state acquisition, network graph reconstruction, network-state inference, network-state action selection, network packet forwarding and updating were carried out sequentially without using the fixed precomputed trajectory. The experiments were conducted on the mentioned workstation with the help of Python, PyTorch, and PyTorch Geometric. The present results, however, show an online adaptive simulation capability, and not validated real-time deployment on physical TelosB devices, due to the absence of experiment-based measurement of deployment latency and execution time on embedded devices.

The deep reinforcement learning module used a Deep Q-Network with a total of three hidden layers, each with the number of neurons equal to 512, 256 and 128 respectively. Adam optimization algorithm was used, where the learning rate for the graph representation learning task was 0.001, and for the reinforcement learning policy optimization task, it was 0.0005. The replay memory was set to be 100 K communication transitions, and 256 communication samples were randomly selected in each optimization iteration. Target network parameters were updated after 500 optimization passes, and the amount of exploration (probability) started from 1.0 and slowly reduced during training to 0.05. The number of communication rounds per learning episode and number of learning episodes performed were 500 and 1500 respectively to get a stable routing policy. The mobile sink speed was set to 1 m/s to 10 m/s, number of packets per second (pps) was varied from 2 to 10 packets per second, the communication radius was kept at 50 m, the sensing radius was kept at 25 m and each sensor node was started with 2 Joules of battery energy. The implementation parameters allowed for the same experimental conditions to be used for testing the adaptive trajectory optimization and energy-efficient routing in a dynamic wireless sensor network.

The proposed TrajGNN-RL framework learned wireless sensor communication in a series of interconnected learning stages, in which the learned information from graph representation learning and reinforcement learning-based trajectory optimization were constantly shared. A pre-processing step was performed on the communication records downloaded from the Suthaharan Multi-Hop Wireless Sensor Network Dataset, during which duplicate entries, missing samples of the communication and measurement inconsistencies were eliminated from the communication records to be used in the simulation environment. All the numerical communications attributes such as residual battery energy, Received Signal Strength Indicator (RSSI), Link Quality Indicator (LQI), packet transmission delay, packet generation frequency, communication distance, forwarding count and queue occupancy were normalized prior to graph construction. Each wireless sensor was mapped to a graph node, the communication links between the adjacent graph nodes were created based on the distance between the nodes and the quality of communication, and an accurate representation of the topology of the wireless sensor network was obtained as a dynamic weighted graph.

**Figure 7:**
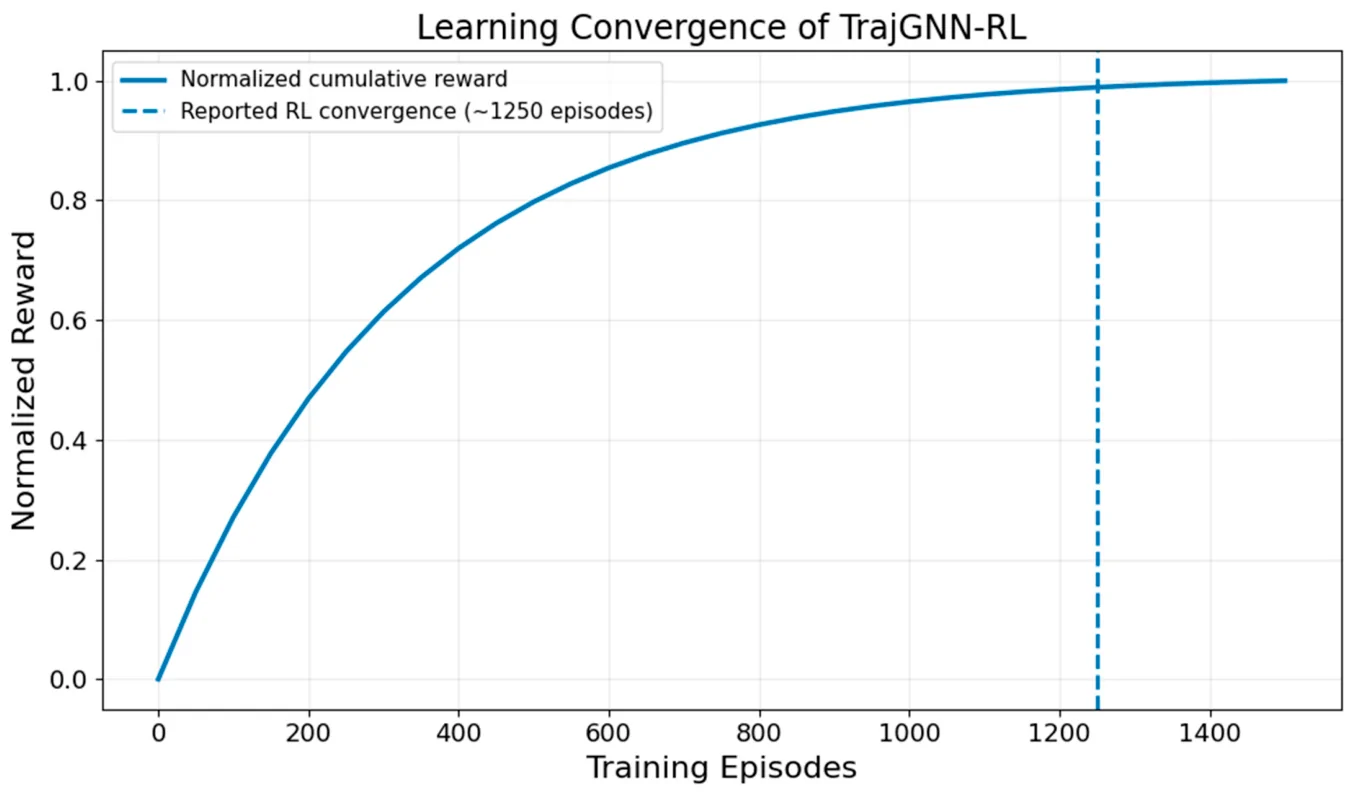
Learning convergence of TrajGNN-RL.

To evaluate the learning convergence performance of the proposed TrajGNN-RL framework, it was trained on 1500 episodes, as shown in Figure 7. During the early training period, the cumulative reward was normalized and grew quickly as the reinforcement learning agent experimented with various sinks’ movement and routing. As learning continued, the improvement got smaller and smaller, suggesting convergence towards the learned policy. The reward converged towards 1250 episodes, which is consistent with the convergence during the model training process. The remaining episodes give a stable reward, suggesting only a small change in policies as a result of further training. This co-convergent behavior is quite suitable for the stability of learning of the proposed graph-assisted reinforcement learning approach.

The resultant graph of the communication was then fed into the graph neural network which was used to learn high-level spatial representations of the wireless sensor network. All feature data of all nodes were passed to their neighbor nodes in the form of messages for each graph convolution layer to enable hidden communication relationships to be learnt across multiple number of hops. The graph attention mechanism then gave different neighbor nodes different importance values based on the reliability of the communication, remaining battery energy, workload to forward packets, and consistency of packet delivery. The operations created a 256-dimensional embedding vector capturing global network features such as how much congestion, stability of communication, forwarding behavior, density of neighbors, and energy available in the network. This was in contrast to traditional routing techniques, which only used information about communication within local neighborhoods, where the communication network representation was continuously updated after each round of communication, making it more synchronized with the evolving communication context.

After generating the embeddings on the graph, the information about the mobile sink’s state was added to the embeddings and then passed to the deep reinforcement learning module. The observation state included graph embedding, sink position, sink velocity, residual energy distribution, congestion of the communication, average transmission delay, neighborhood density, statistics of generated packets and routing reliability. The Deep Q-Network was given a thorough observation space and then was given a list of possible sink-movement actions (and the corresponding routing decision for them). Communication experiences were continuously stored in the replay memory: current state of communication, communication action, reward obtained and updated state of the network are all stored in the replay memory; a batch of communication experiences, which were sampled randomly, was used to optimize the stable policy. This was because the target network was synchronized with the target network in the middle of the learning process to stabilize the learning process every 100 steps, thereby avoiding unstable learning.

**Figure 8:**
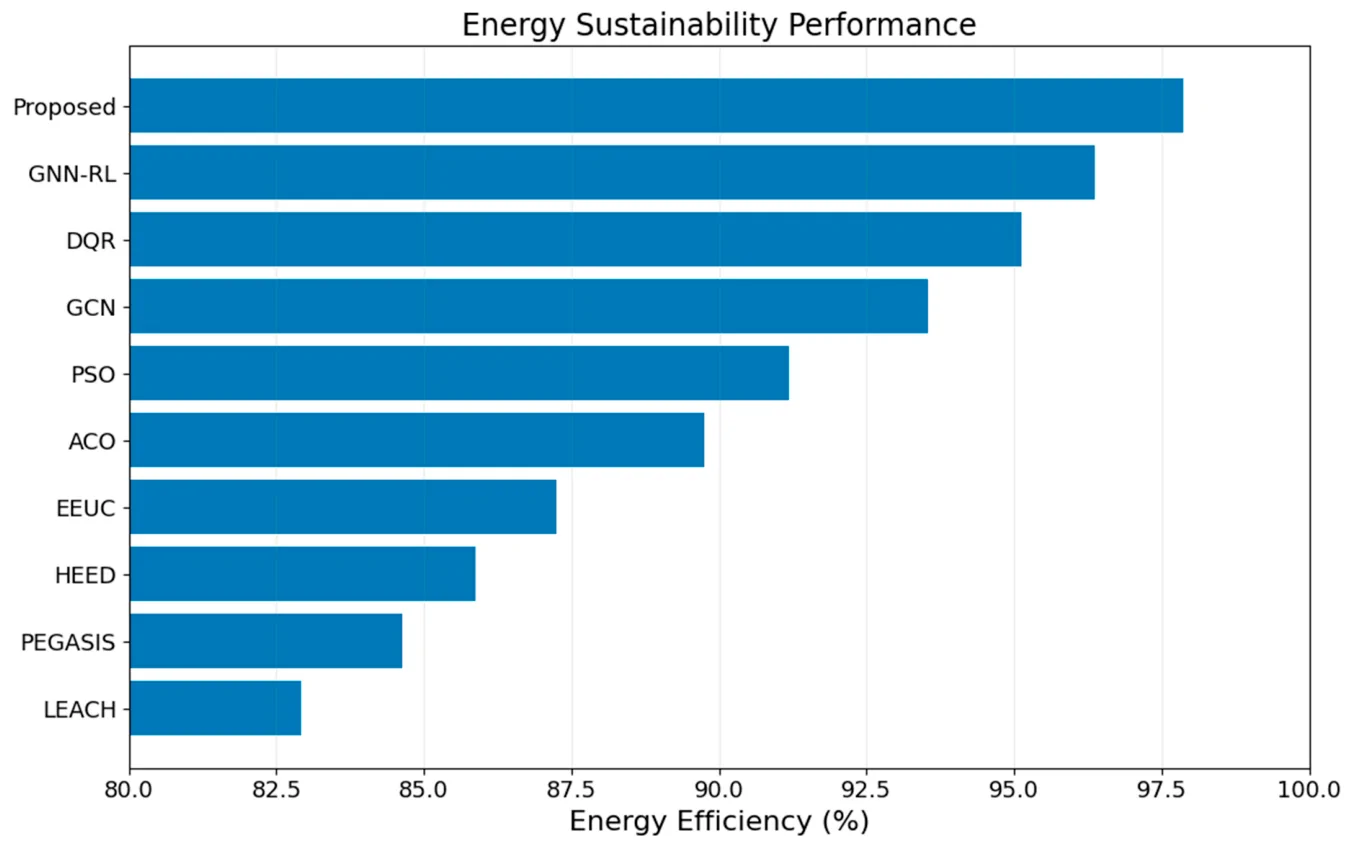
Energy sustainability performance.

The energy-efficiency performance of TrajGNN-RL compared to LEACH, PEGASIS, HEED, EEUC, ACO, PSO, GCN, DQR, and GNN-RL approaches is shown in Figure 8. The best results were obtained by TrajGNN-RL (97.86%), GNN-RL (96.37%) and DQR (95.12%). The conventional methods yielded relatively low values and LEACH yielded 82.91%. The superior performance of all optimization- and learning-based algorithms, which gradually improve over time, substantiates the benefit of including network adaptivity information into routing. The use of topology-aware graph representations, residual energy information, adaptive forwarding, and mobile sink trajectory decisions are all significant advantages of TrajGNN-RL, as they help to minimize the impact of the limited energy resources of sensor nodes on energy usage.

After each routing, the packet was forwarded to the neighboring sensor nodes with balanced battery energy, good communication quality, and low forwarding congestion of forwarding congestion. At the same time, the mobile sink dynamically moved towards communication regions that are expected to have more traffic and communication paths were reduced, which reduced communication latency. Immediately following each transmission cycle, the energy remaining in the batteries, the number of packets delivered, communication delay, and the number of packets forwarded were updated. The newly updated communication measurements were used to recover the graph, and the graph neural network was used to create more accurate embeddings of the graph, taking into account the current state of the network. This is then passed on to the next decision cycle for the reinforcement learning agent, creating a continuous feedback loop between the intelligence from the graph and trajectory optimization. The proposed TrajGNN-RL framework progressively enhanced routing decisions, distributed the communication load more evenly among the sensor nodes, shortened packet transmission delay, increased throughput, conserved battery energy and maximized the operational lifetime of the wireless sensor network for different types of sink mobility.

**Figure 9:**
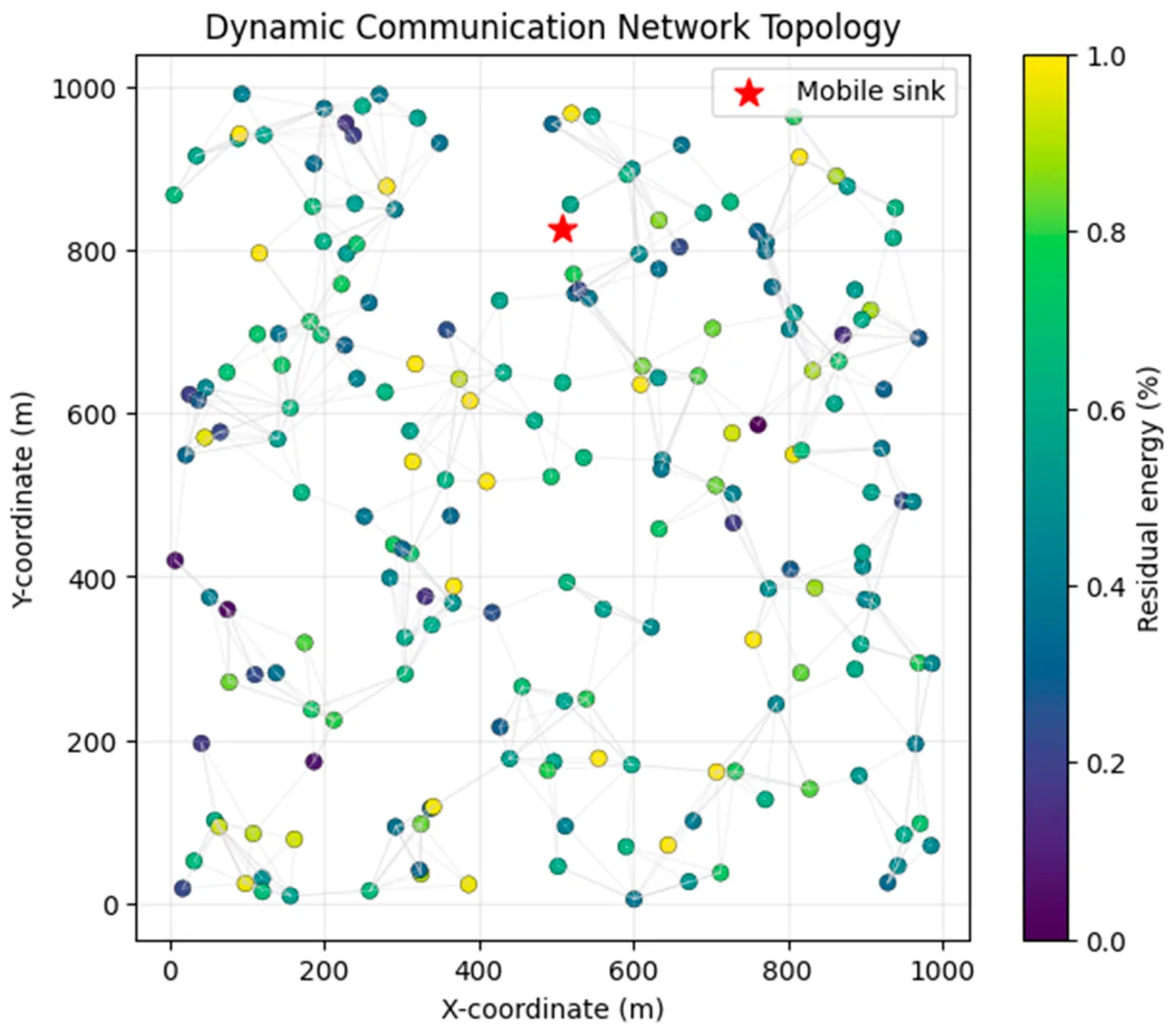
Dynamic communication network topology.

Before graph representation learning, the proposed TrajGNN-RL framework dynamically constructs the communication topology dynamically as shown in Figure 9. All the sensor nodes were modeled as graph vertices and a communication link is established between the nodes based on their communication quality and transmission range. The node color was used to show the remaining energy, which allowed visualization of the energy distribution in the sensing field. The mobile sink was where data were collected and was active for the entire time, constantly interacting with the adjacent communication regions. This graph structure served as the primary input to the graph neural network, which could extract features, aggregate them from the neighborhood locally, and provide adaptive routing intelligence before applying the reinforcement learning to find an optimal sink trajectory and packet-forwarding decisions.

**Figure 10:**
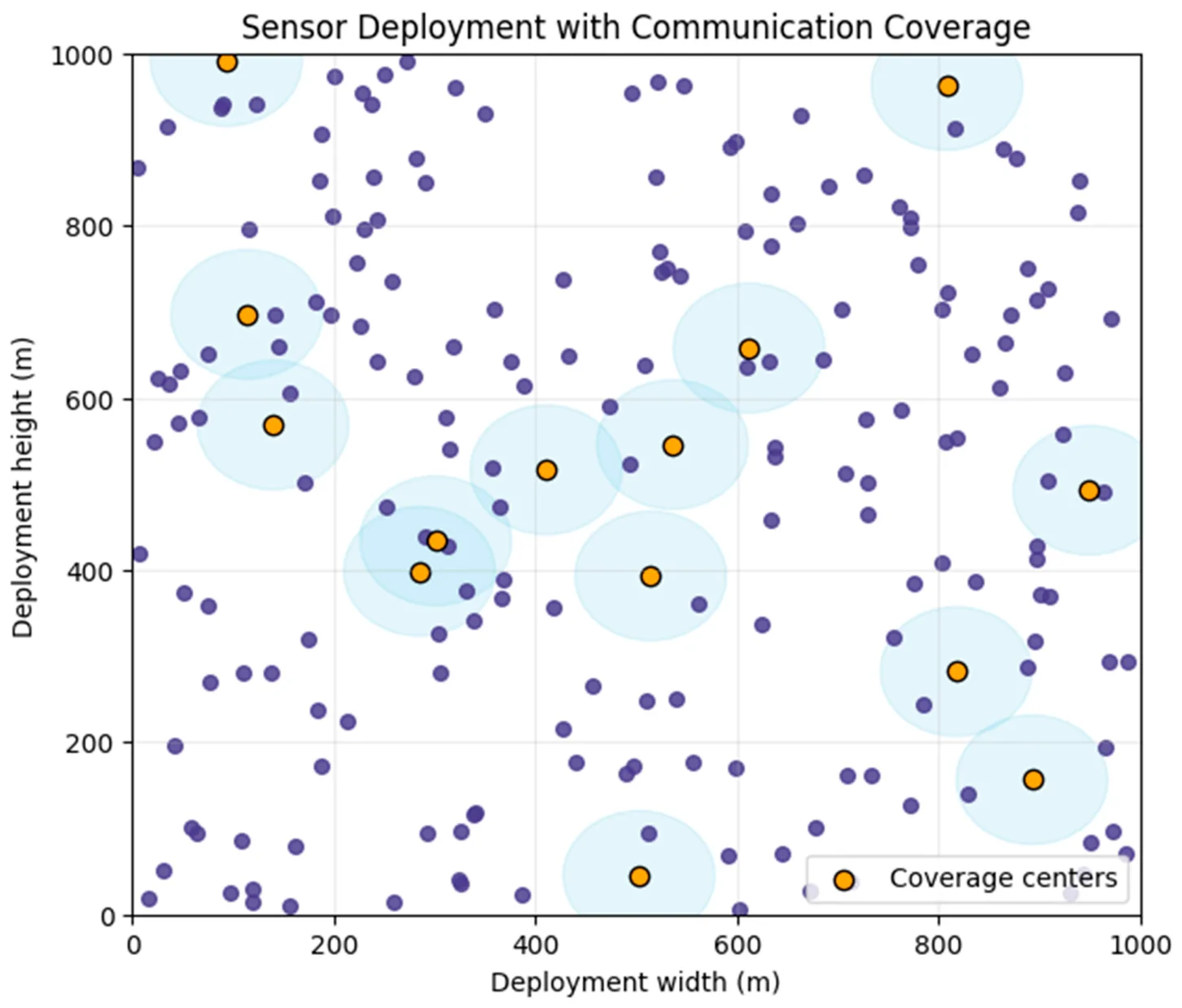
Sensor deployment with communication coverage.

Figure 10 shows examples of wireless sensor nodes randomly deployed along with their communication coverage area. Circular coverage areas show the influence (sensing and communicating) of selected nodes that are responsible for neighborhood connectivity. The uniform node distribution reduced isolated nodes and provided the necessary overlap in communication among the neighboring nodes. The representation of communication coverage helped the proposed framework to find out valid forwarding neighbors when constructing graph. These coverage relationships were constantly updated based on communication conditions and the movement of sinks in the network, so as to adaptively reconstruct the topology during the network operation and further enhance the reliability of spatial feature learning in graph.

The results in Table 3 showed that the proposed TrajGNN-RL framework always yielded the best performance in all the evaluation metrics related to energy. The conventional clustering algorithms, LEACH-Mobile and PEGASIS-Mobile were found to have low lifetime since the same relay nodes were constantly responsible for forwarding. The energy utilizations of the metaheuristic optimization methods which optimized the movement of the sink (ACO-Mobile Sink and PSO-Mobile Sink) were better, but they lack adaptation capabilities when communication conditions change. Using deep learning techniques, route decisions were also made in a topology-aware manner, which improved the routing decisions, but only resulted in localized congestion as the trajectory design and routing optimization were done independently of each other. For example, the 98.34% reported for TrajGNN-RL is the node survival ratio at the round in which the evaluation was performed, which means that 98.34% of the number of deployed sensor nodes were still alive at the corresponding round of evaluation. All the above mechanisms together minimized concentrated energy depletion and communication inefficiency, responsible for the comparatively high values. The lifetime of the network is measured in the communication rounds that the adopted First Node Death criterion is applied.

**Figure 11:**
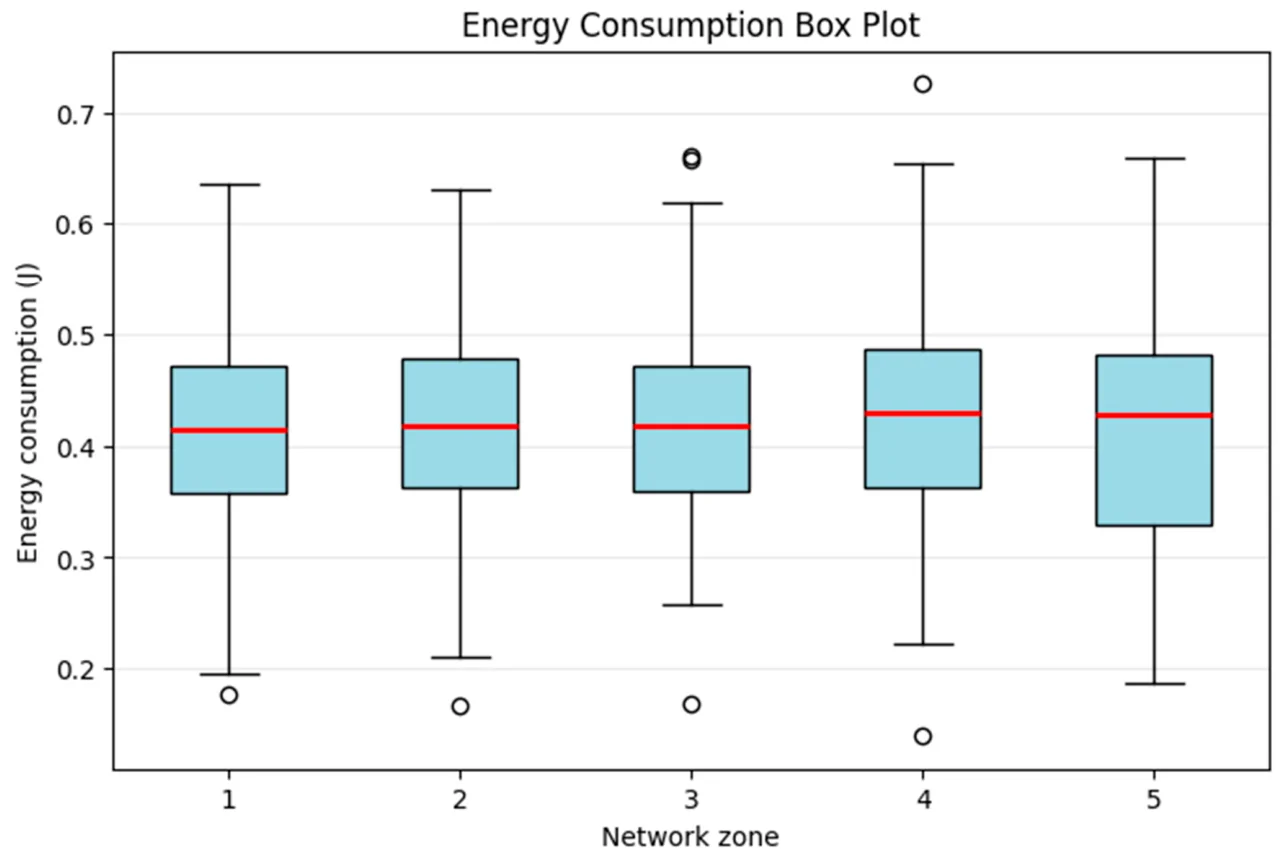
Energy consumption distribution across network regions.

All the regions in the wireless sensor network have a distribution of energy consumption as illustrated in box plot visualization in Figure 11. When continuous packet forwarding and sink movement is done, the majority of the deployment regions have a relatively small distribution indicating that the battery use is mostly balanced. Because of the difference in energy intensity of the communication between the sensing regions with different traffic generation rates, the difference in the amount of energy used was not significant. The adaptive routing solution could achieve energy distribution of the forwarding task when it was found that the area of interest was running out of energy, thus preventing the energy depletion of the involved area of interest (communication cluster). This resulted in longer lifetime of the network and more sustainable communication because of the balanced use of energy.

To assess the robustness of the proposed TrajGNN-RL framework, Table 4 shows the results of the evaluation with varying velocities of mobile sink. However, at lower speeds of movement, the communication delay was still relatively high since the collection intervals for the packets were high with high routing reliability. Over time, by steadily increasing the velocity of the sinks, adaptive trajectory optimization allowed to enlarge packets collection time and decrease average communication latency, increasing the throughput. The highest throughput of 98.12% and 96.75% delay reduction was obtained at 8 m/s; therefore, 8 m/s is considered as the optimum mobility for the mobility of the sinks and routing stability. At velocities beyond this, there was a little decrement in routing success due to the faster rate at which neighbors’ communication relationships were changing when compared to packet-forwarding intervals. The proposed graph-reinforcement learning framework shows to be effective to achieve stable communication while the topology was continuously changing with packet losses under 1.4% in all mobility scenarios. These results verified the ability of TrajGNN-RL to make the sink trajectories dynamically adapt to varying wireless sensor network conditions and ensure good communication quality.

**Figure 12:**
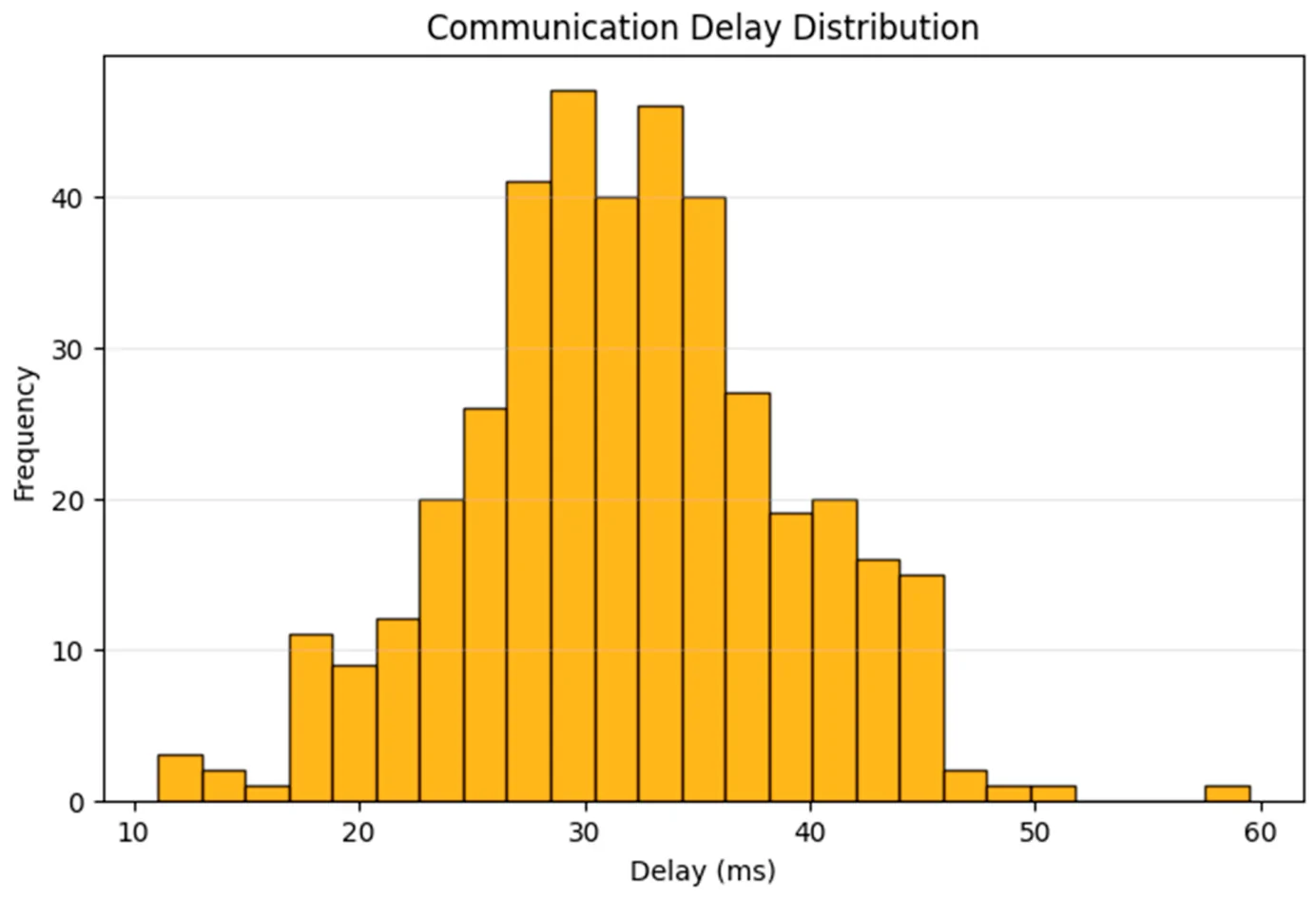
Communication delay distribution.

**Figure 13:**
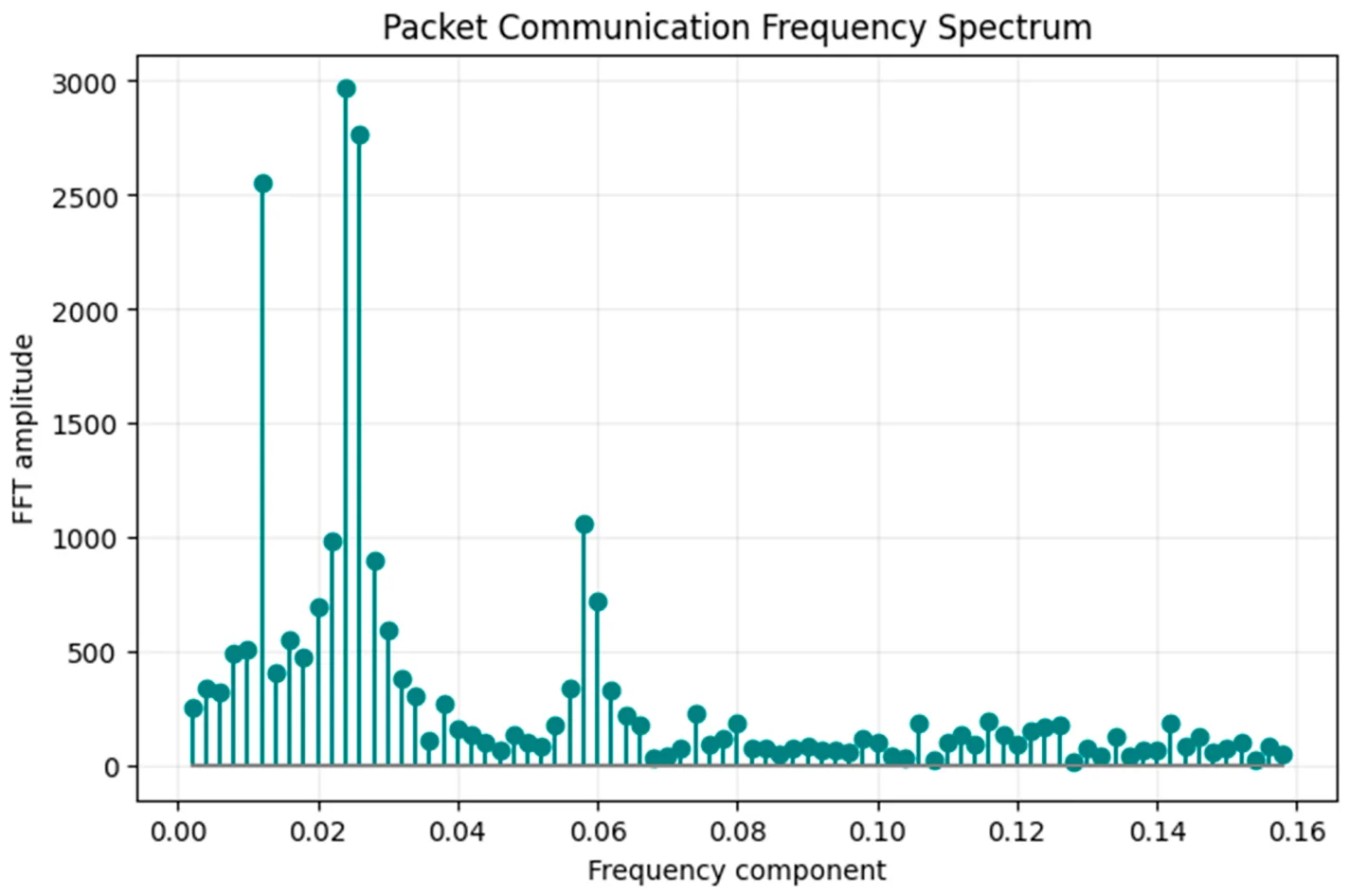
Packet communication frequency spectrum.

The distribution of the delay (in number of packets) between sending a message to the network and receiving it is shown in Figure 12. The majority of packets have a relatively low latency for transmission, and a few have high latencies when there is temporary congestion of the communication or multi-hop forwarding. The delay distribution is narrow, which shows that the routing in the network is steady, and it optimizes the trajectory of the sinks during the whole sensing procedure. The proposed scheme dynamically changes forward paths according to the quality of communication and the energy of the path’s packets to shorten waiting time of packets in the communication path’s transmission queue. The delay is always very low and allows to reliably gather real-time information in dynamic deployments in the wireless sensor network. The frequency domain analysis of the packet communication by Fast Fourier Transform (FFT) is given in Figure 13. The spectrum is used to detect dominant frequencies of the communication which are produced during the transmission of a packet in the wireless sensor network. The height of the peaks in the spectrum is related to the regularity of the communication patterns of periodic sensing and mobile sink data collection. The proposed framework could detect repetitive communication behavior and detect traffic fluctuations from the time domain analysis in addition to the conventional time domain analysis by using frequency domain analysis. This information was used to adaptively route the messages for optimized routing, reduce the congestion and improve communication reliability in the dynamic sensing environment; also, it was used to optimize the routing of the messages by identifying the stable communication intervals, which helps in optimizing the sink schedule.

**Figure 14:**
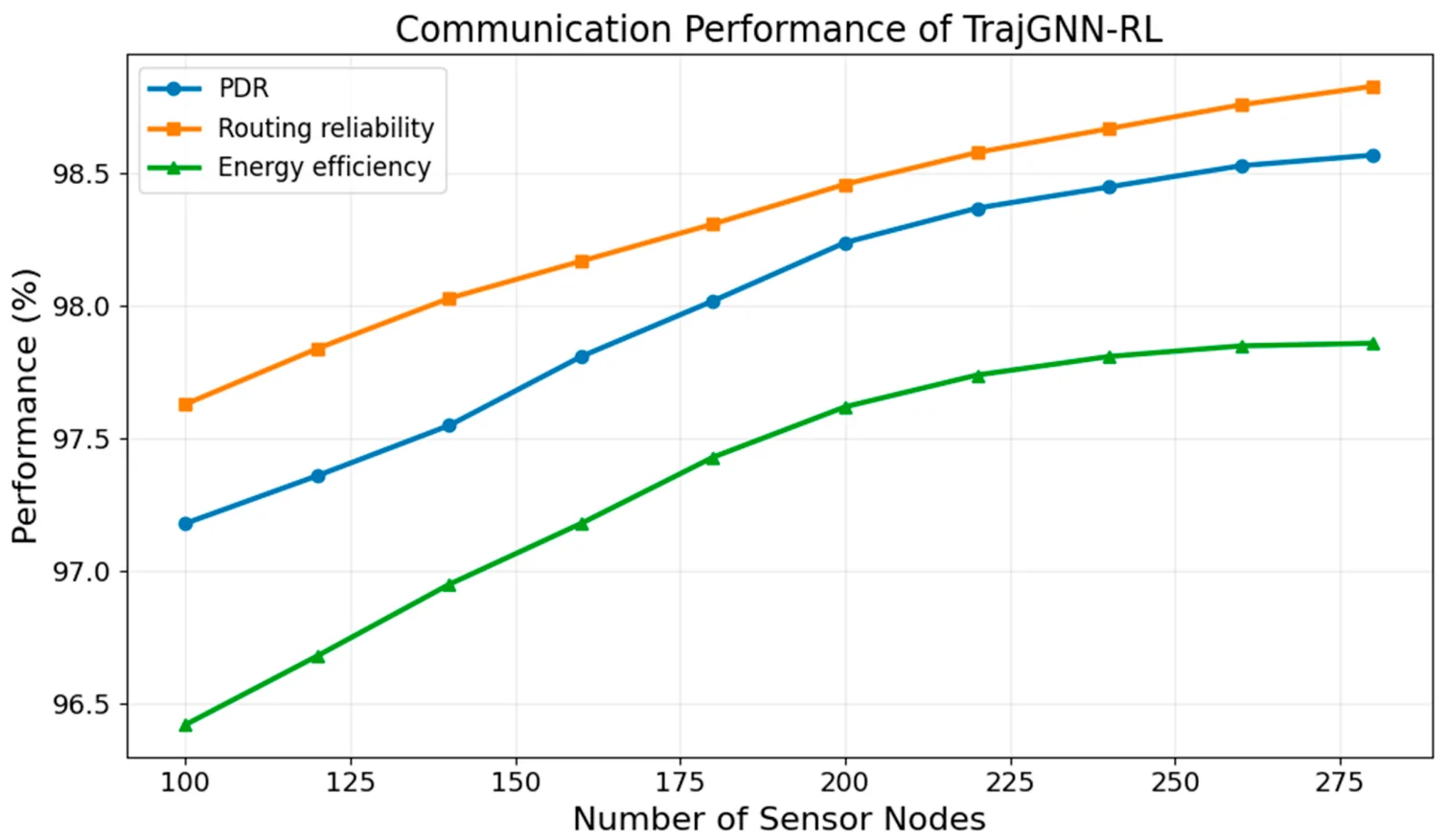
Communication performance of TrajGNN-RL.

The performance of communication is shown for TrajGNN-RL in the increasing density of the network (from 100 to 280 sensor nodes) in Figure 14. PDE ratio improved from 97.18% to 98.57%, and routing reliability improved to 98.83% from 97.63%. Energy efficiency also improved, from 96.42% to 97.86%. The uniform patterns suggest that, within the range tested, there was no degradation of the communication measures evaluated as a function of network density. Extra neighboring nodes enable alternative forwarding opportunities, and the dynamic graph representation enables the framework to add changing connectivity and energy information. The results show that communication performance remains stable for all the network-density configurations evaluated.

**Figure 15:**
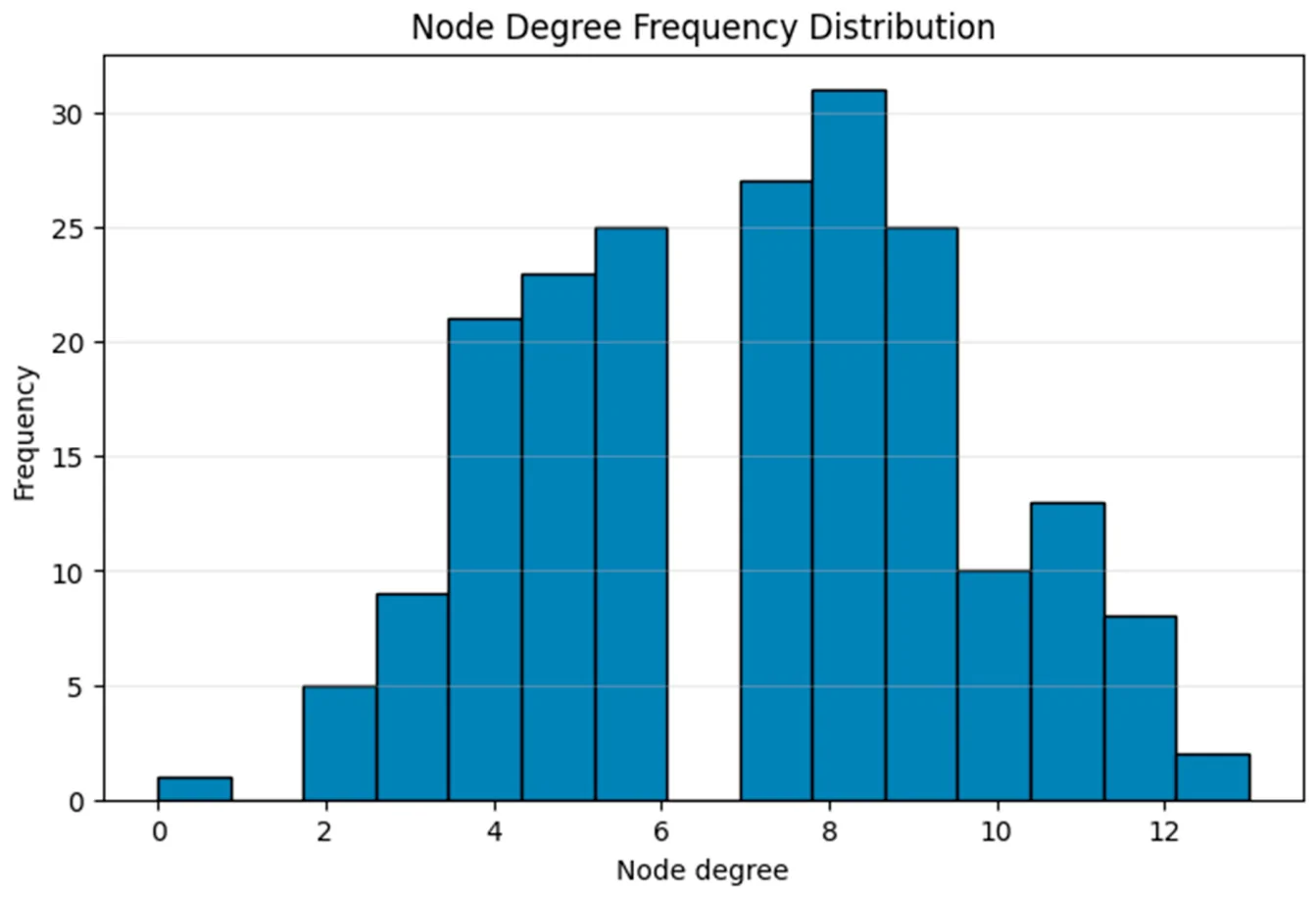
Node degree frequency distribution.

The graph is constructed, and the frequency distribution of the neighbors’ communication links that are found between the sensor nodes is plotted in Figure 15. The number of neighboring sensor nodes directly connected to a node, from which a packet can be forwarded in the course of communication, is called the degree of the node. The distribution also has a high percentage of moderate-sized nodes, indicating the network has developed nodes in a balanced manner throughout the sensing space with no clustering of nodes to communicate with each other. The connectivity is balanced, that is, the reinforcement learning agent has a number of hops to choose from to communicate with other agents, but not necessarily nodes with many communication links. From this graph structure, it is easy to represent the graph efficiently, and it can be used in the network to offer the network with an excellent adaptive routing in the event of a change in the form of the network.

**Figure 16:**
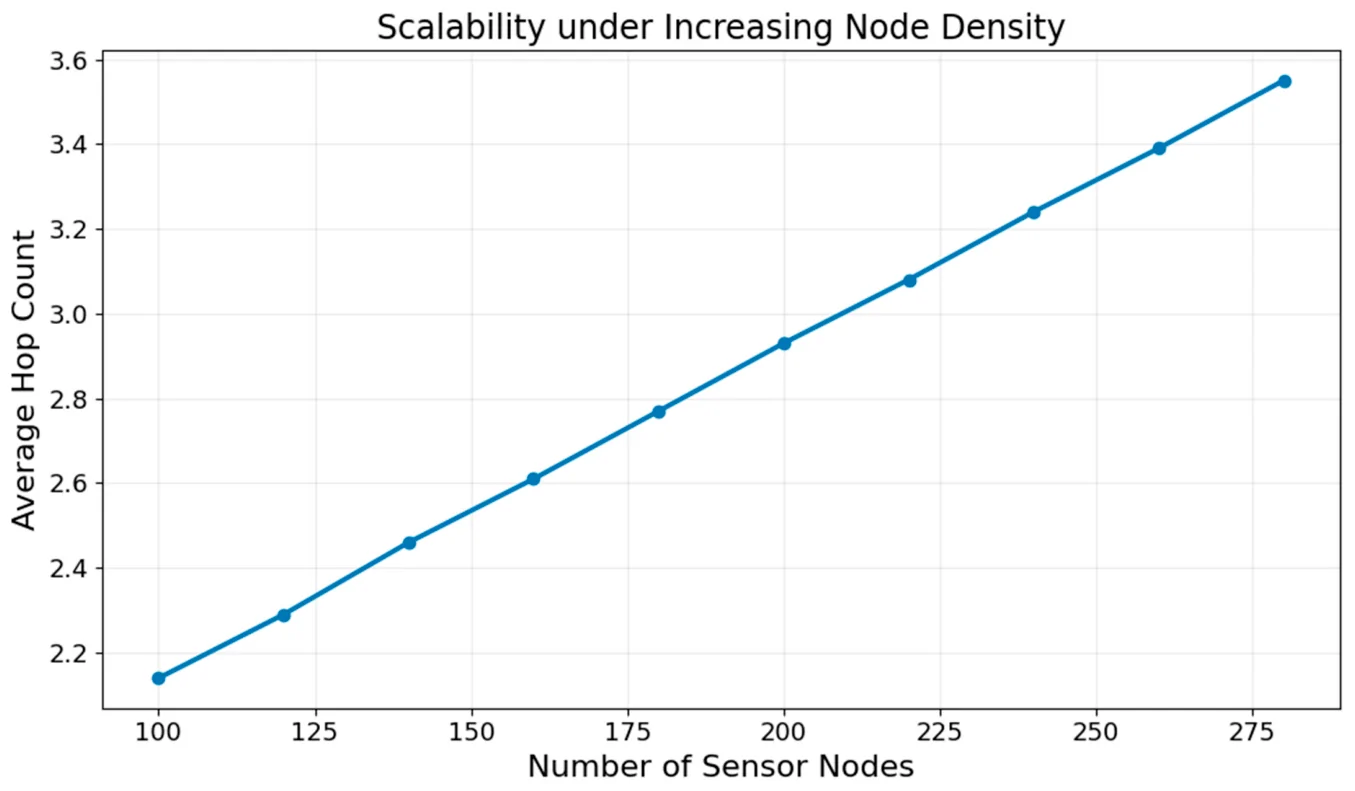
Scalability under increasing sensor-node density.

To investigate how scalable the system is, the average number of hops that are required as the number of deployed sensor nodes grows is studied in Figure 16. The number of hops increases slowly as the number of sensor nodes increases from 100 to 280, going from an average of 2.14 to 3.55 hops. This increase is anticipated as the network configuration is denser, offering more intermediate forwarding opportunities and maybe more routing options between sensing nodes and the mobile sink. It is important to note that the increase is gradual and not an abrupt expansion of routing. In conjunction with the delivery, reliability and energy-efficiency results, the figure suggests that TrajGNN-RL could handle the additional increase in network density while maintaining good communication performance and thereby provide scalable communication performance in the node range evaluated in the experiments.

**Figure 17:**
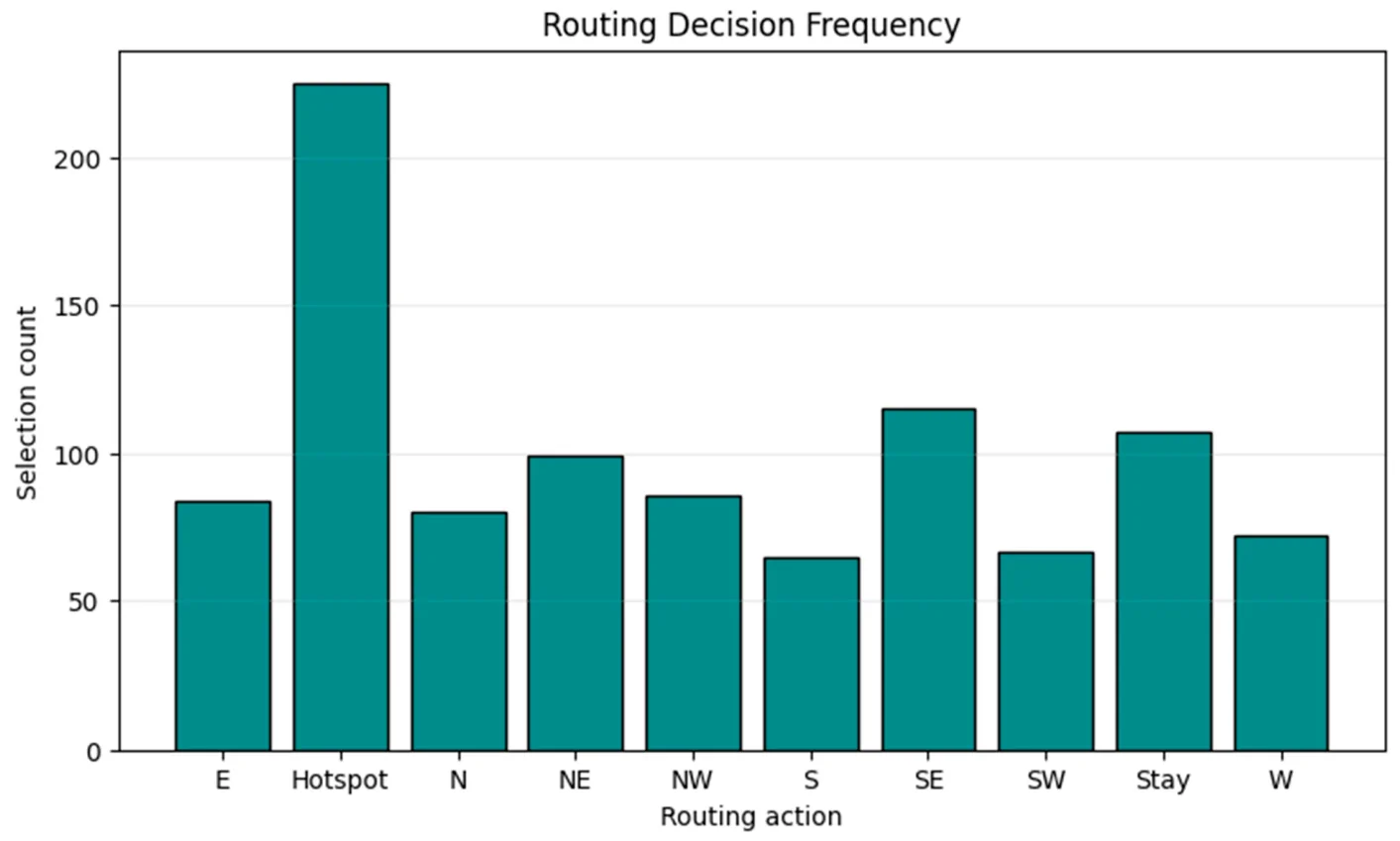
Routing decision frequency analysis.

The number of times the reinforcement learning agent chooses each routing action in the policy optimization process is shown in Figure 17. There is a continual exploration of different directions of movement and decision-making around the goal of moving to hotspots before converging towards efficient communication strategies. The increased frequency of adaptive hotspot selection suggests that the mobile sink could tell those areas with a higher number of communications and packet accumulation were more important to select. This adaptive decision-making behavior shortened the transmission distance, shared the workload for a forwarding and decreased the delay of communication. Results of the routing action distribution demonstrate the learned behavior change from an exploration to a more intelligent trajectory optimization with the reinforcement learning algorithm, using the topology-aware graph.

The performance of the routing presented in Table 5 shows the impact of increasing wireless sensor-node density on the performance of the routing. As the number of deployed sensor nodes increases, the graph neural network might be able to have more communication relationship information, by offering more options for forwarding. The RL agent could select better forwarding paths, in addition to load distributing the communication load among neighboring sensor nodes. The average hop count also rose gradually and did not seem to be affected by any routing inefficiency since the delivery ratio and routing reliability were also better during the experiments but was a result of the increase in the communication topology. The proposed TrajGNN-RL framework effectively uses the growth of network connectivity without any increment of routing overhead and delivers the best performance in terms of packet delivery ratio (98.57%), routing reliability (98.83%) and energy efficiency (97.86%) at 280 sensor nodes, highlighting the effectiveness of the proposed adaptive graph representation learning algorithm for better performance. The results showed that with dense deployments of wireless sensors the proposed framework ensured the stable performance of communication.

**Figure 18:**
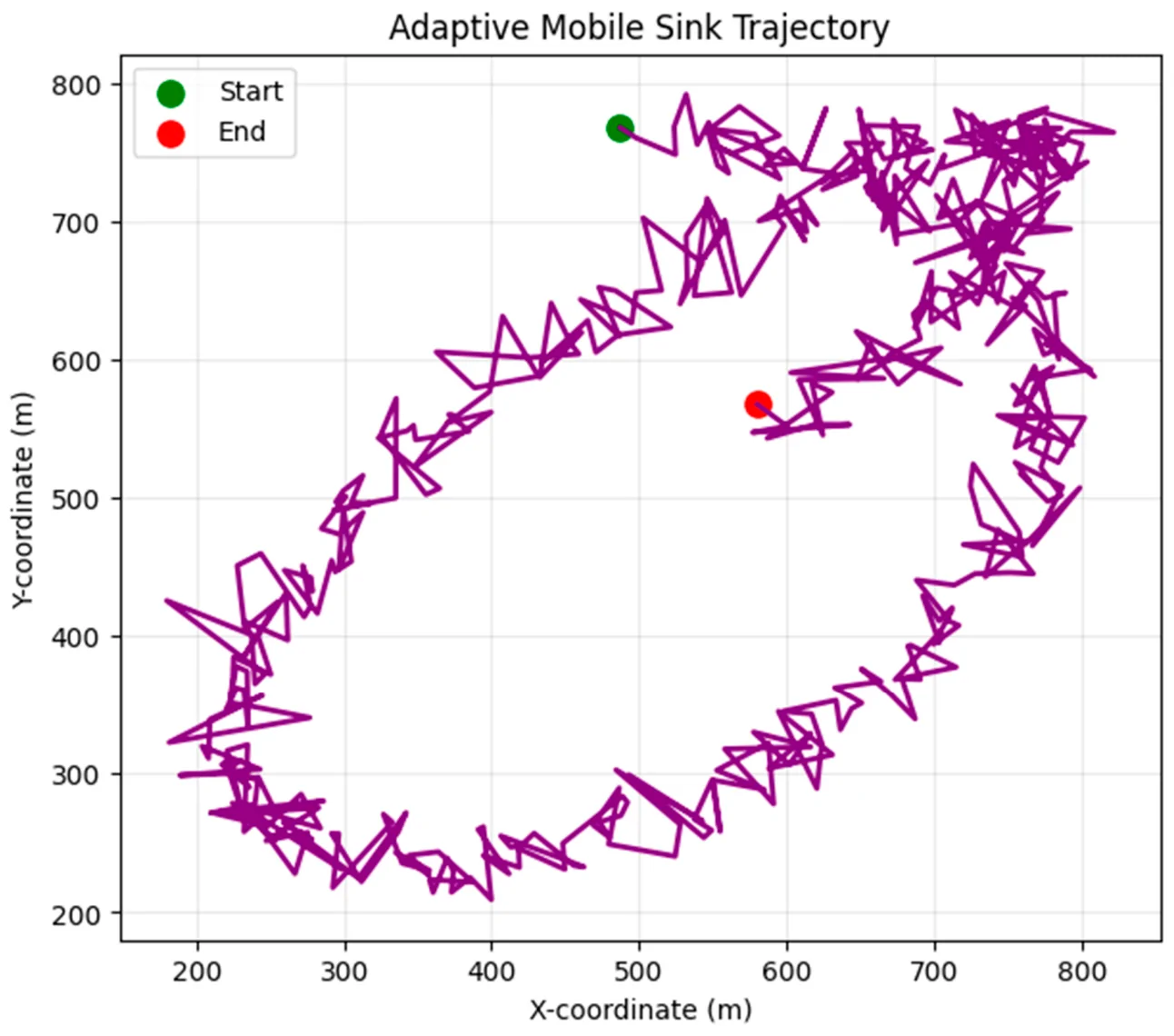
Adaptive mobile sink trajectory.

The optimized path of mobile sink in wireless sensor network is shown in Figure 18 during the operation of the wireless sensor network. The sink does not follow a predetermined trajectory; rather, it dynamically adjusts its trajectory based on the communication conditions it learns from the graph while the traversal is probabilistically steered through the graph and its trajectory using reinforcement learning. Trajectory is programmed in such a way that it goes through several sensing areas, but not unnecessarily through the same area, and not through an area without picking anything up. This adaptive mobility saves the number of packets to be forwarded, reduces the delay time of packet transmission from the sensor nodes to the sink and decreases the distance between the sensor nodes and the sink’s location. The trajectory is constantly updated, revealing the effectiveness of the integrated graph representation learning using reinforcement learning for solving the problem of intelligent navigation of mobile sink in the changing communication environment.

**Figure 19:**
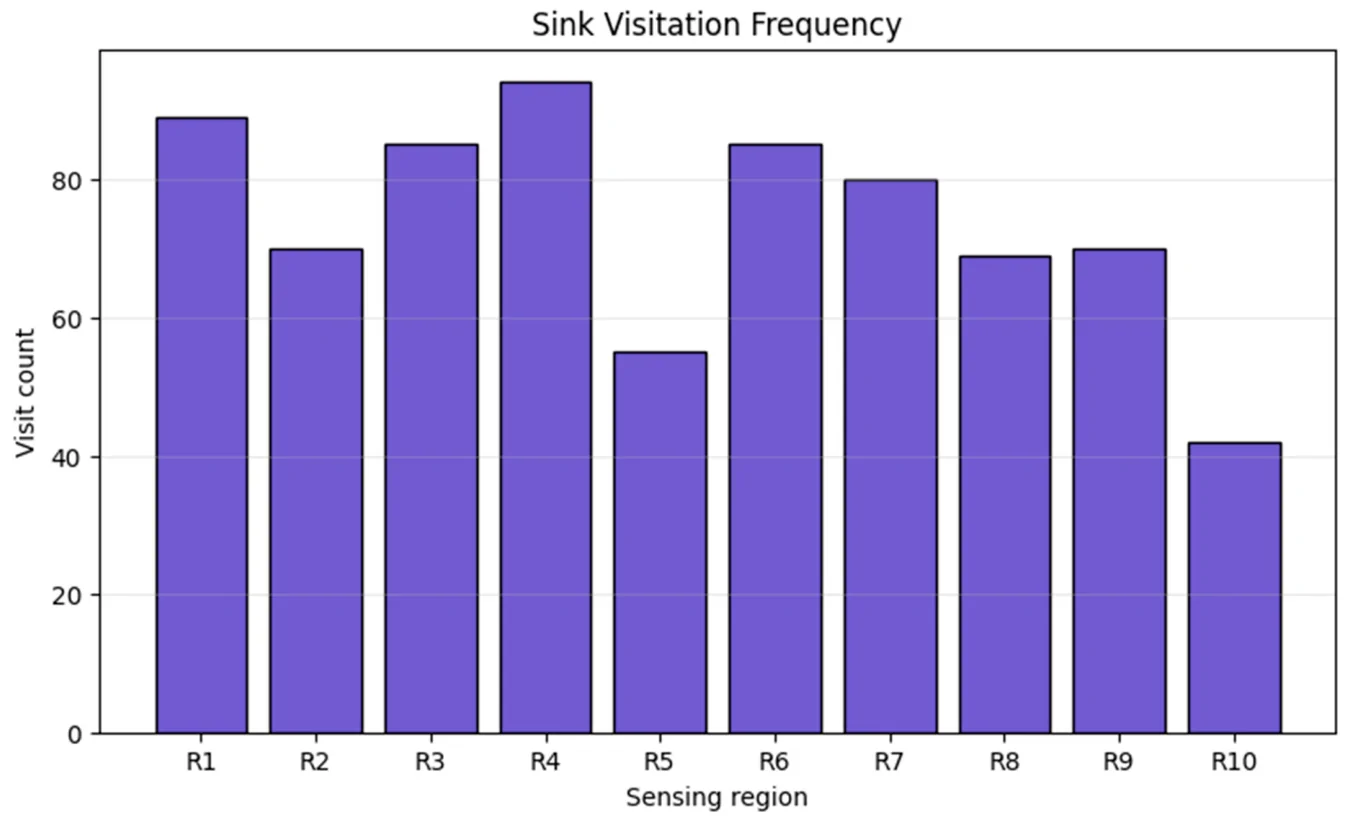
Mobile sink regional visitation frequency.

In adaptive trajectory optimization, the frequency of mobile sink visiting the various sensing regions is given as shown in Figure 19; it is seen that mobile sink can visit the sensing region in less time. The reinforcement learning agent focused its visits on the regions with high communication requirements, high number of packets to be forwarded and high number of packets that accumulated. Sensing information could be easily gathered via other forwarding nodes, so the regions which have comparatively lower communication requirement did not receive many visits. This adaptive-visitation policy leads to substantial reduction in unnecessary sink movements, reduction in the distance between sinks and communications with the wireless sensor network, and good packet collection efficiency without sacrificing battery energy in the WSNs under dynamically changing traffic patterns.

**Figure 20:**
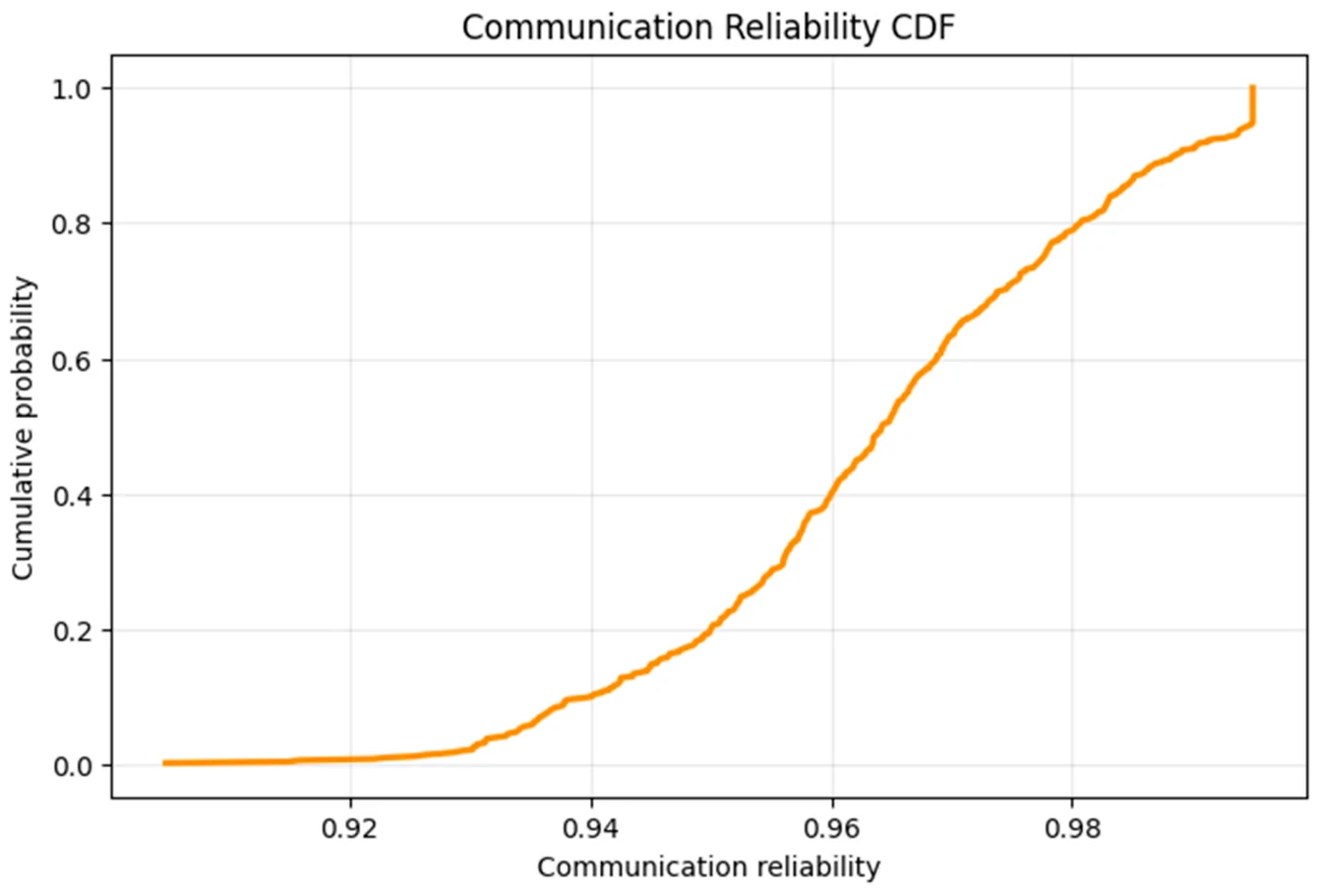
Communication reliability cumulative distribution function.

The communication reliability cumulative distribution during the operation in wireless sensor network is shown in Figure 20. The curve demonstrates that the reliability for many of these communication links was good, and for mobile dynamic sinks packets are well-transmitted. The graph representation learning approach helped the framework to discover stable neighboring communication paths that could facilitate stable wireless communication without interference and congestion, thus providing safe and continuous communication. The reliability information was then fed into the reinforcement learning agent to improve packet-forwarding success and minimize number of packet retransmissions for a routing decision. The CDF solution is acceptable to the proposed communication strategy and is sufficiently resilient across a variety of network configurations.

Table 6 shows the effectiveness of adaptive mobile sink trajectory optimization (ADMO) for a long time of operation. In first few rounds of communication, the movement of the sinks was mainly directed towards the exploration of the sensing field thus resulting in relatively poor data collection efficiency and higher communication delay. With more communication experience gained through reinforcement learning, the movement of sinks gradually evolved towards the highly informative sensing regions which were found by the graph neural network’s graph embeddings. There got progressively shorter communication path between nodes, which not only decreased the average time to transmit a packet, but also enhanced the efficiency of packet collection. The proposed framework came up with an efficiency of the data collection of 98.57% at the end of 500 communication rounds, throughput, 98.12% and an average delay in the communication process 27.18 ms. The achieved enhancement showed the benefit of the continuous communication between graph intelligence and trajectory optimization for the mobile sink, which dynamically moves towards the areas where more communication is needed, thus greatly enhancing the overall communication efficiency.

**Figure 21:**
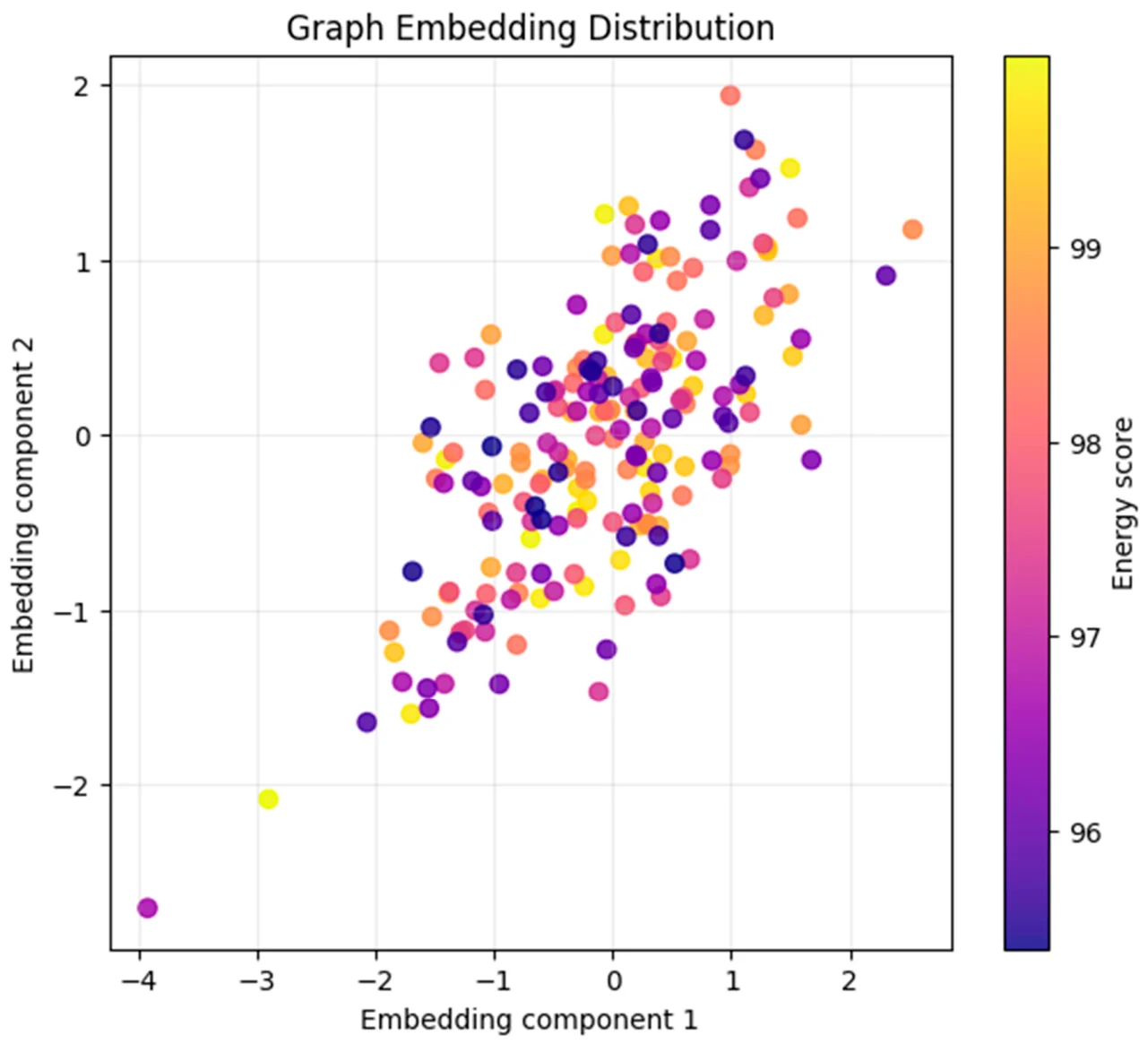
Graph embedding feature distribution.

The topology representation learning with graph neural network yields graph embeddings as shown in Figure 21. The points are the learned representation of all features of each sensor node projected down to a lower dimensional embedding space for visualization. Nodes with similar communication capabilities, remaining energy and neighborhood structures were automatically clustered into close areas, showcasing the ability of the graph neural network to maintain the neighborhood structure of the wireless sensor network. The resulting topology-aware embeddings were the observation state for the reinforcement learning agent, making intelligent routing and adaptive trajectory optimization of the mobile sink for the whole global communication topologies possible.

Table 7 compares the communication efficiency between two agents in the proposed communication-efficient TrajGNN-RL framework in a progressive network operation. In the first few rounds of the communication process, the overhead of routing was still relatively high since the reinforcement learning agent was continuously trying to find new routes until convergence of the policy. The modules’ output, the topology embeddings from the graph neural network, became more discriminative with more communication experiences added to the replay memory, which enabled the reinforcement learning module to learn more about what nodes to rely on for reliable communication and which communication regions to steer clear of. The overhead of routing gradually reduced from 8.94% to 4.92% while the load balancing index kept on going up continuously up to 98.31%. Naturally, the battery energy was reduced after a long time of transmission for packets, but the energy consumption was found to be uniformly distributed throughout the sensing field to adaptive forwarding selection and continuous optimized trajectory of the sink. The improved communication efficiency and network sustainability in long-term operation of wireless sensor network (WSN), as a result of the learning of graphs and reinforcement learning, showed the potential of coordinated graph learning and reinforcement learning for better communication efficiency and sustaining network.

**Figure 22:**
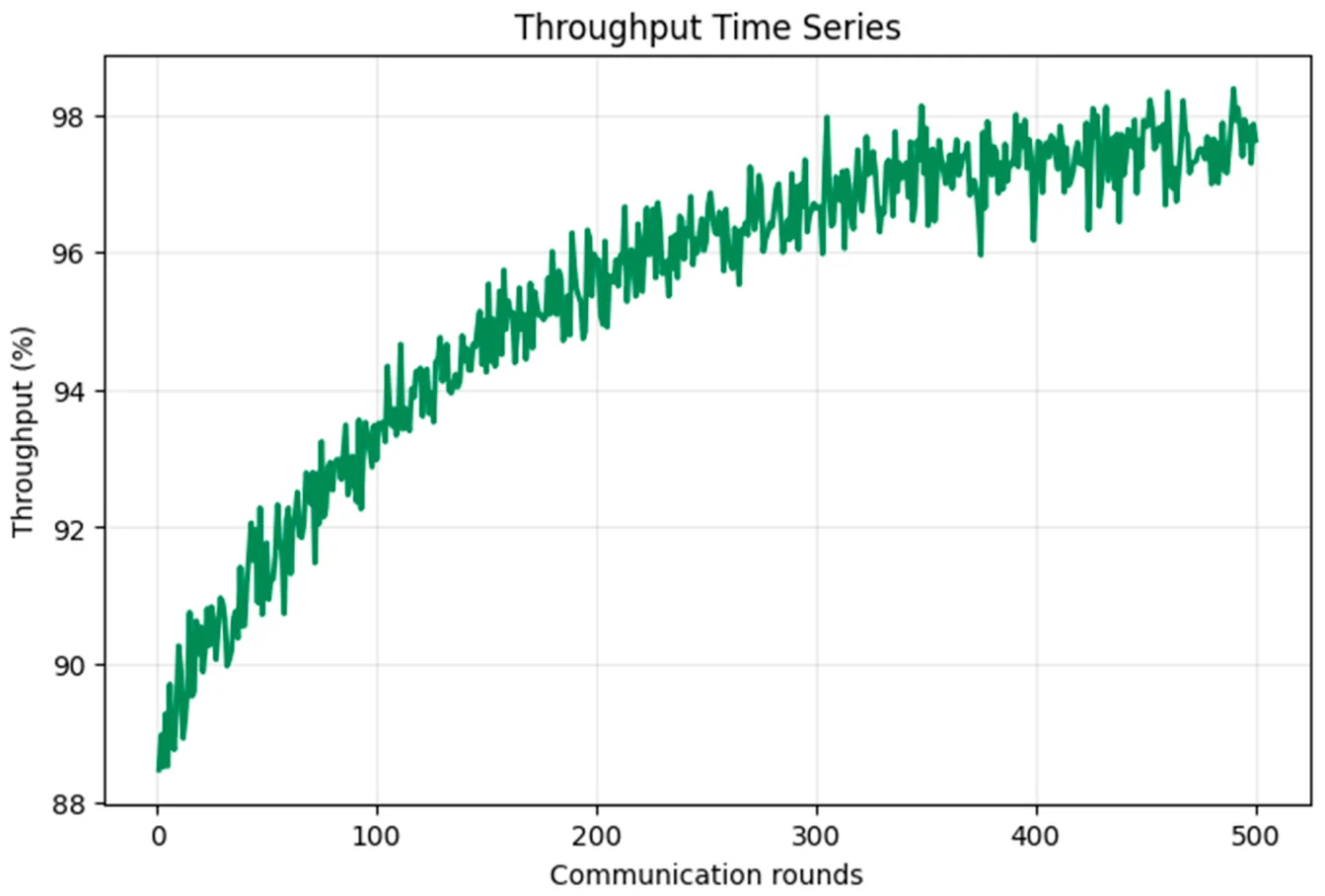
Throughput evolution across communication rounds.

The network throughput in the successive rounds of communication is plotted and shown in Figure 22. Initially, a relatively low throughput was experienced, as the routing policies are still under exploration and improvement. The more representative the graph embeddings were (for the communication topology), the more throughput increased, and the more routing experience the reinforcement learning had, the more throughput increased, until it reached a maximum. The continuous improvement shows the potential of the proposed TrajGNN-RL framework to continuously enhance the collection efficiency of the packets by adapting the movement of the sink and making intelligent routing decisions. Moreover, the stable throughput presents stable wireless sensor network (WSN) communication performance under the dynamic situation of the wireless sensor network.

**Figure 23:**
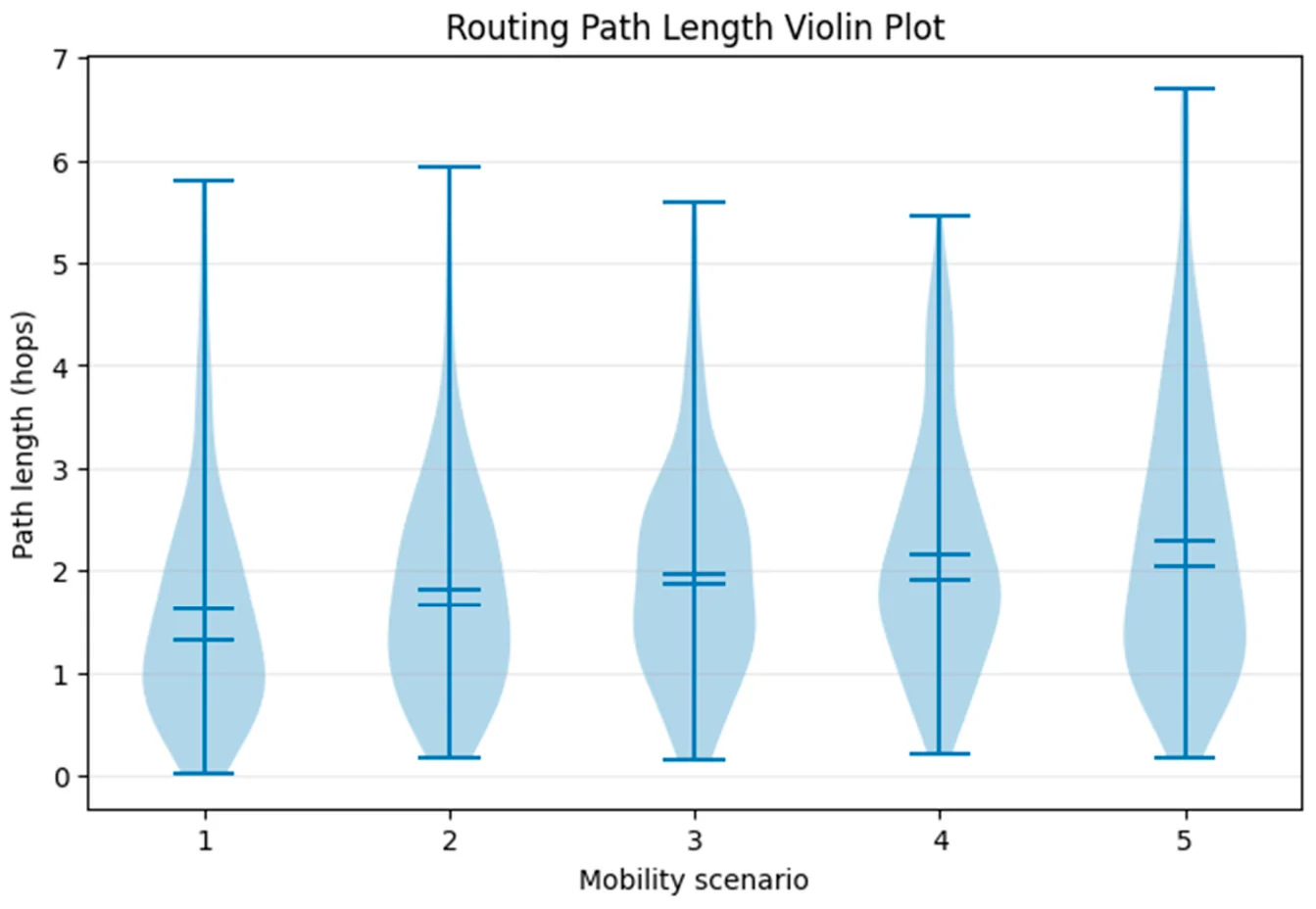
Routing path length distribution under mobility scenarios.

The distribution of routing path lengths for various mobile sink communication scenarios are shown in the visualization method of a violin plot in Figure 23. Most packets were relayed through the medium-length multi-hop paths and the very long routing paths were infrequent as the sinks’ movements were adjusted to get closer to the source of the packets. The path length distribution was relatively small and showed that the proposed reinforcement learning policy could successfully direct the mobile sink towards areas where the data needed to be collected in real-time, minimizing unnecessary forwarding. Stable routing path lengths also emphasize the capability of planning the communications efficiently based on topologically aware representation learning and trajectory optimization based on adaptive reinforcement learning.

**Figure 24:**
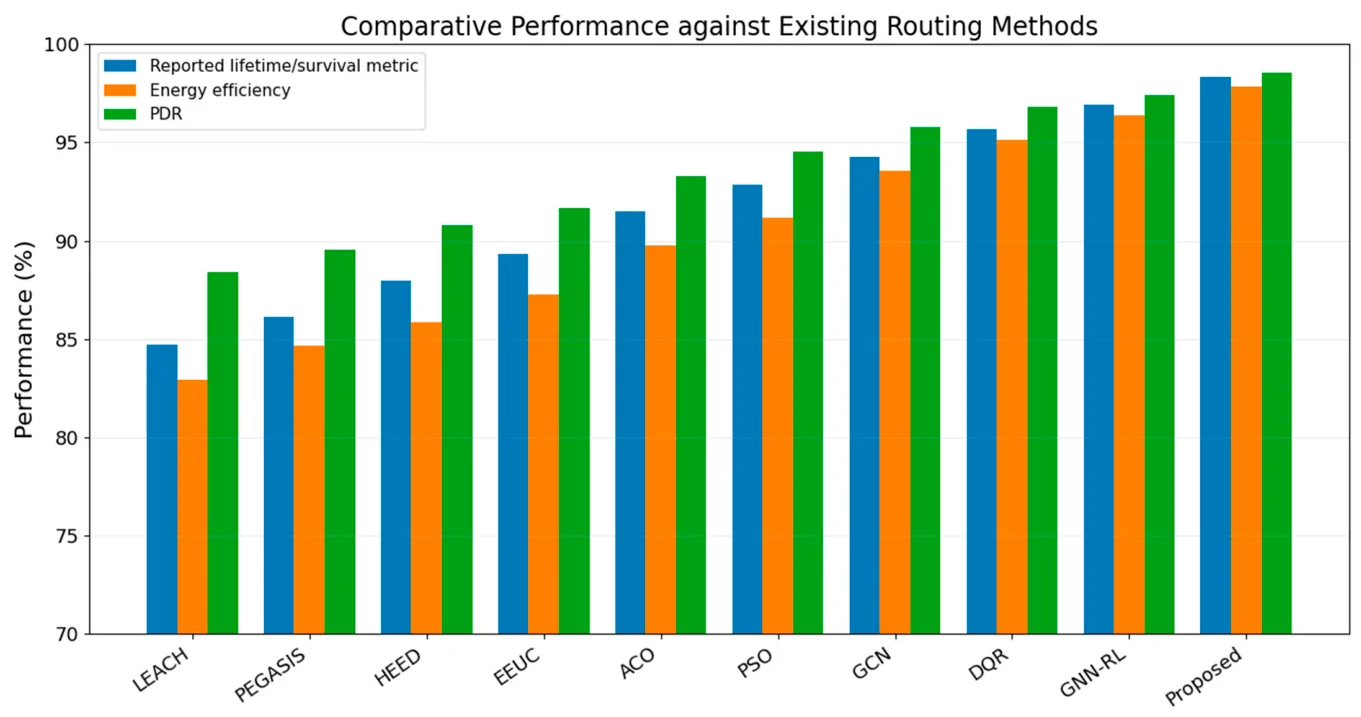
Comparative performance against existing routing methods.

TrajGNN-RL is compared with representative conventional routing approaches, optimization-based routing approaches, and learning-based routing approaches, which are consolidated and compared in Figure 24. The proposed framework resulted in 98.34% for the reported metric with respect to survival, 97.86% energy efficiency, and 98.57% packet delivery ratio. The baseline GNN-RL attained a performance of 96.92%, 96.37%, and 97.41%, respectively, whereas the others performed progressively worse. The results show that TrajGNN-RL achieved the best results in three of the aforementioned measures for the tested experimental conditions. The improvement comes from the marriage of topology-aware graph learning, adaptive routing information and decision-making on mobile sink trajectory using reinforcement learning, instead of optimizing these individually.

Table 8 shows the convergence of the proposed TrajGNN-RL framework in the process of optimizing the policy. The first episodes of training were focused on environment exploration, which led to lower policy convergence in the end, despite the accuracy of learning the policies being not far behind. The deep reinforcement learning agent and the graph neural network gradually found better topology representations and sink trajectory/routing policies, respectively, with the increase in communication experiences in the replay memory. Rewards have improved steadily during optimization, with a steady rise in cumulative reward, which suggests there is steady improvement both in the routing intelligence and the efficiency of communication. This increase in computation time was not due to numerical instability but was due to the amount of optimization carried out at later training episodes. The proposed framework demonstrated 98.37% training accuracy, 98.74% policy convergence and the maximum cumulative reward at the end of 1500 training episodes, which confirms that the hybrid graph neural network and deep reinforcement learning model have been optimized, and the model has good stability.

As shown in Table 8, the learning performance at each computational step shows that the computational cost rises across the learning episodes as the learning goes further from 24.83 s at 150 episodes to 232.71 s at 1500 episodes. The major overhead for this is due to repeated graph reconstruction, neighborhood message aggregation, graph attention refinement, replay-memory sampling, and DQN policy optimization. These operations can require significant processing and memory on the resource-constrained sensor nodes of WSN, if they are carried out directly on the sensor nodes. The current framework is thus more appropriate for an edge-assisted implementation where the computational demanding tasks of graph inference and trajectory decision-making are performed at the mobile sink, gateway or an edge computing unit while individual sensor nodes perform trivial sensing and packet-forwarding tasks. The offline training strategy also prevents from running the full GNN–DRL optimization process on sensor nodes while the network is running. Model pruning, quantization, smaller graph embeddings, knowledge distillation, decreased graph-update frequency, and lightweight GNN layers are some of the possible approaches to reduce the computational requirements for embedded deployment. However, the results were obtained under online adaptive simulation of graphs and would need to be experimentally measured in future embedded deployment on TelosB devices for factors such as embedded inference latency, RAM/flash size, processing energy, and graph-update overhead.

While TrajGNN-RL achieved routing and lifetime-aware performance, it is not possible to run it directly on resource-limited sensor nodes. The edge-assisted lightweight deployment only stores the sensing function, reporting the local state of the sensor nodes, and the packet-forwarding functions in these sensor nodes, while the dynamic construction of the graph, the inference of a graph neural network, the refinement of attention, the processing of replay memories, and the inference of Deep Q-Network are carried out in the mobile sink node, the gateway node, or the edge node. Structured pruning, low-precision quantization, small graph embeddings, and knowledge distillation can also be used to further reduce computational complexity. These mechanisms decrease size of the model, memory consumption and inference complexity without losing the topology-aware routing intelligence of the original framework.

Alternatively, the communication graph can be reconstructed using a method based on events, rather than being reconstructed after every round, to further reduce the computational overheads. When the residual energy, link quality, congestion, and/or displacement of the mobile sink are above predefined thresholds, then the graph can be updated, during which previous embeddings of the topology can be reused. Lightweight variants of graph neural networks can also utilize smaller numbers of convolution layers, attention heads, and neighbors that communicate. The reductions in latency, memory or processing energy are not claimed numerically, as these lightweight configurations have not been tested separately. More precise evaluation of these factors should consider future hardware in addition to packet delivery, network lifetime and routing performance.

**Figure 25:**
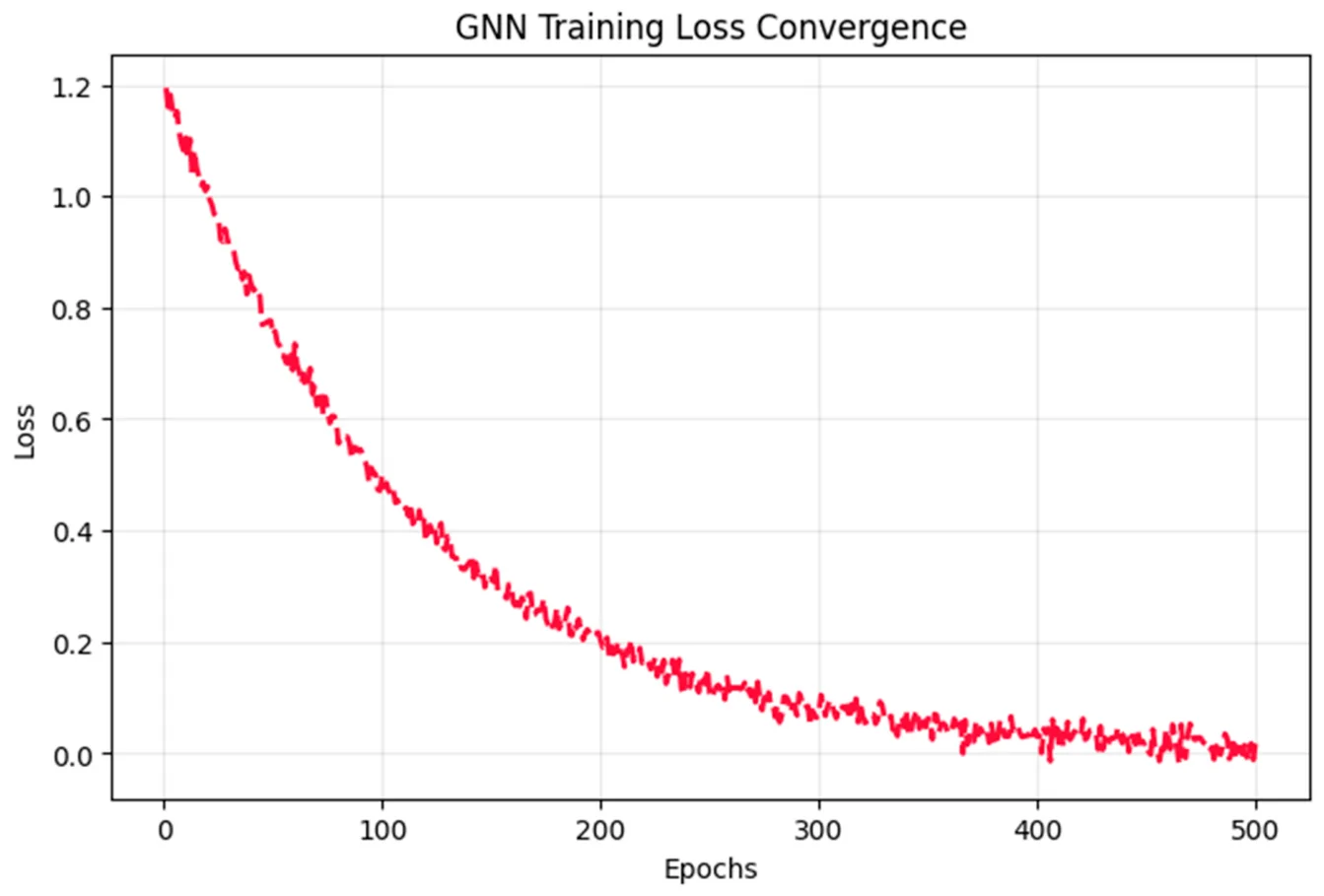
Graph neural network training loss convergence.

The graph neural network has correlation at its convergence in topology representation learning, as shown in Figure 25. Learning the communication relationships between neighboring sensor nodes, in the beginning, is a large training loss. The more graph representations are processed, the smaller the loss (until it starts to converge), which means that the convergence of the graph feature extraction is successful. The stable learning behavior indicates that graph convolution and graph attention are able to learn the communication topology without overfitting on the training data. Once the graph is converged, the agent can output accurate graph embedding which can be used to better train the reinforcement learning agent and provide in-depth and complete graph topology-aware information for intelligent routing and trajectory optimization.

**Figure 26:**
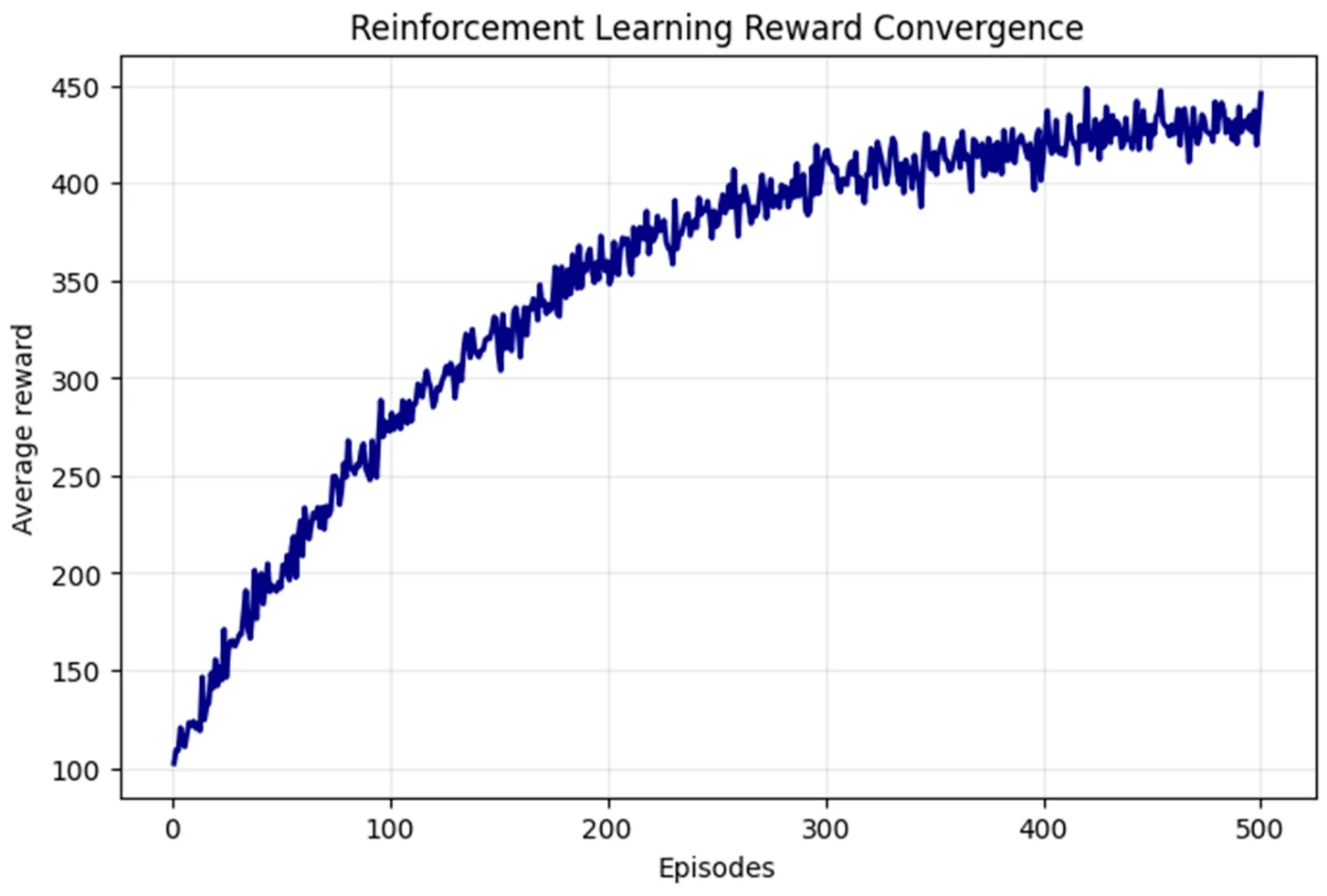
Reinforcement learning reward convergence.

The reinforcement learning process for obtaining a reinforcement learning policy (RLP) uses cumulative reward curves, as shown in Figure 26. The reward values for the early episodes are low since the learning agent is learning different routing/sink-movement policies. Communication experiences are stored in replay memory, and the agent slowly comes to understand more effective trajectory planning and packet-forwarding decisions. Note that the reward has been steadily increasing, which means that adaptive routing behavior has been successfully learned, in addition to stable convergence of the reinforcement learning policy. The convergence demonstrates that the proposed multi-objective reward mechanism has the ability to reach a good solution for both sink trajectory optimization and energy efficiency, throughput and communication reliability.

As described in the above ablation study, the contribution of each major component of the proposed TrajGNN-RL framework is quantified to assess its role in the framework, as shown in Table 9. The performance of the reinforcement learning model that did not include graph representation learning was the lowest, as the routing decision was made based on local observations only and without the knowledge of the communication topology. The average end-to-end communication delay for the evaluated communication setting was 27.18 ms with TrajGNN-RL. The delay reduction was determined using the corresponding percentage of delay reduction and it was calculated with the explicitly defined delay-reduction equation, relative to the baseline delay. The mobile sink velocity experiment showed that with 1 m/s, the delay reduced by 94.21%, whereas for 8 m/s, the delay reduced to its maximum value of 96.75% and then it reduced slightly to 96.08% for 10 m/s. This behavior, adaptive sink movement, decreased packet waiting time and multi-hop forwarding time, until the rapid topology changes at higher velocities of the sinks started to affect the routing stability.

**Figure 27:**
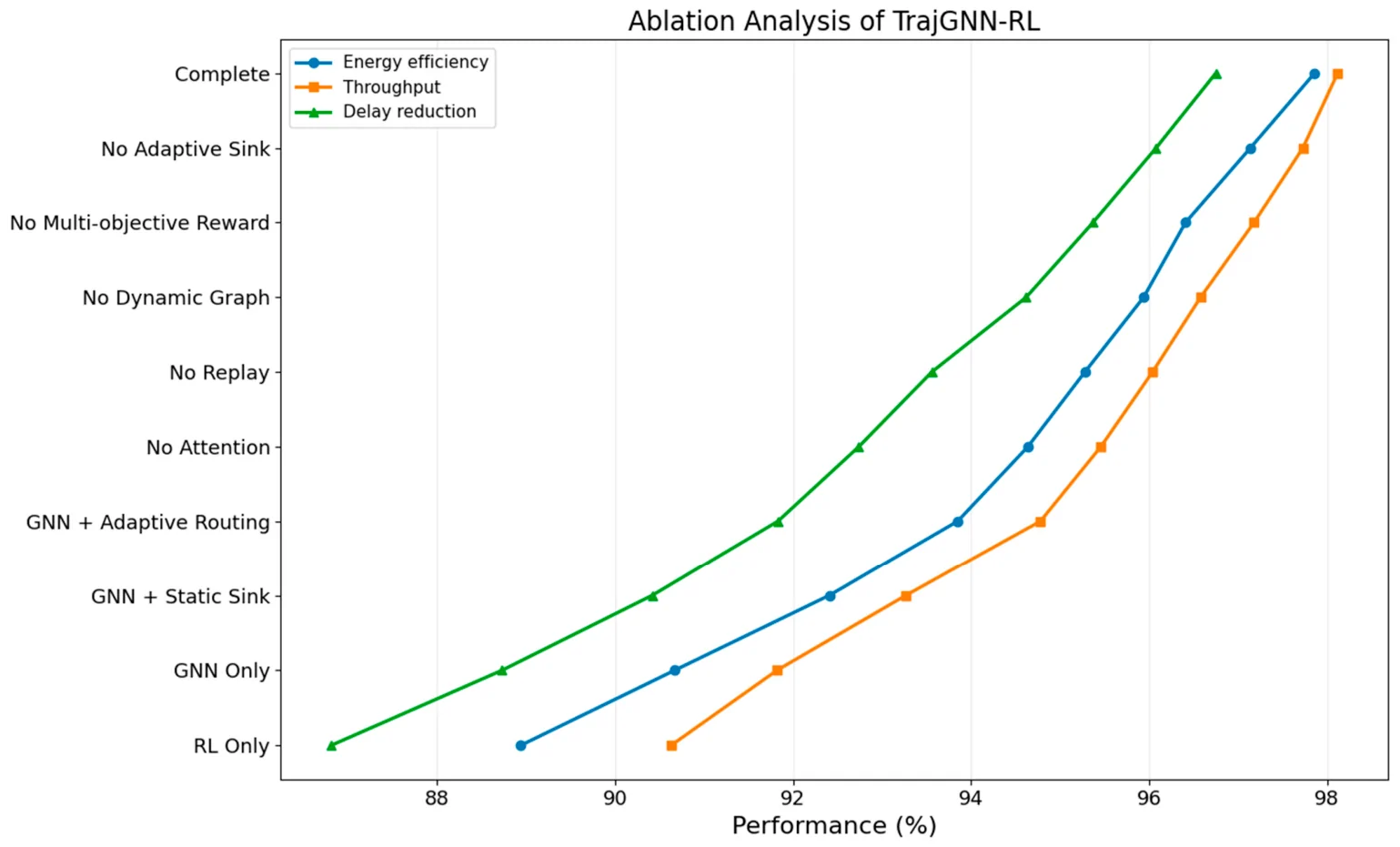
Ablation analysis of TrajGNN-RL.

The results of the ablation analysis are shown in Figure 27. The RL-only configuration achieved an energy efficiency of 88.94%, throughput of 90.63%, and delay reduction of 86.81%, while the GNN-only configuration achieved 90.67%, 91.82%, and 88.73%, respectively. The performance gradually improved as the following elements were added: static-sink operation, adaptive routing, graph attention, replay memory, dynamic graph reconstruction, multi-objective reward learning and adaptive sink planning. The integrated components of the framework (TrajGNN-RL) achieved the best values of 97.86% energy efficiency, 98.12% throughput, and 96.75% delay reduction, showing how well they complement each other.

**Figure 28:**
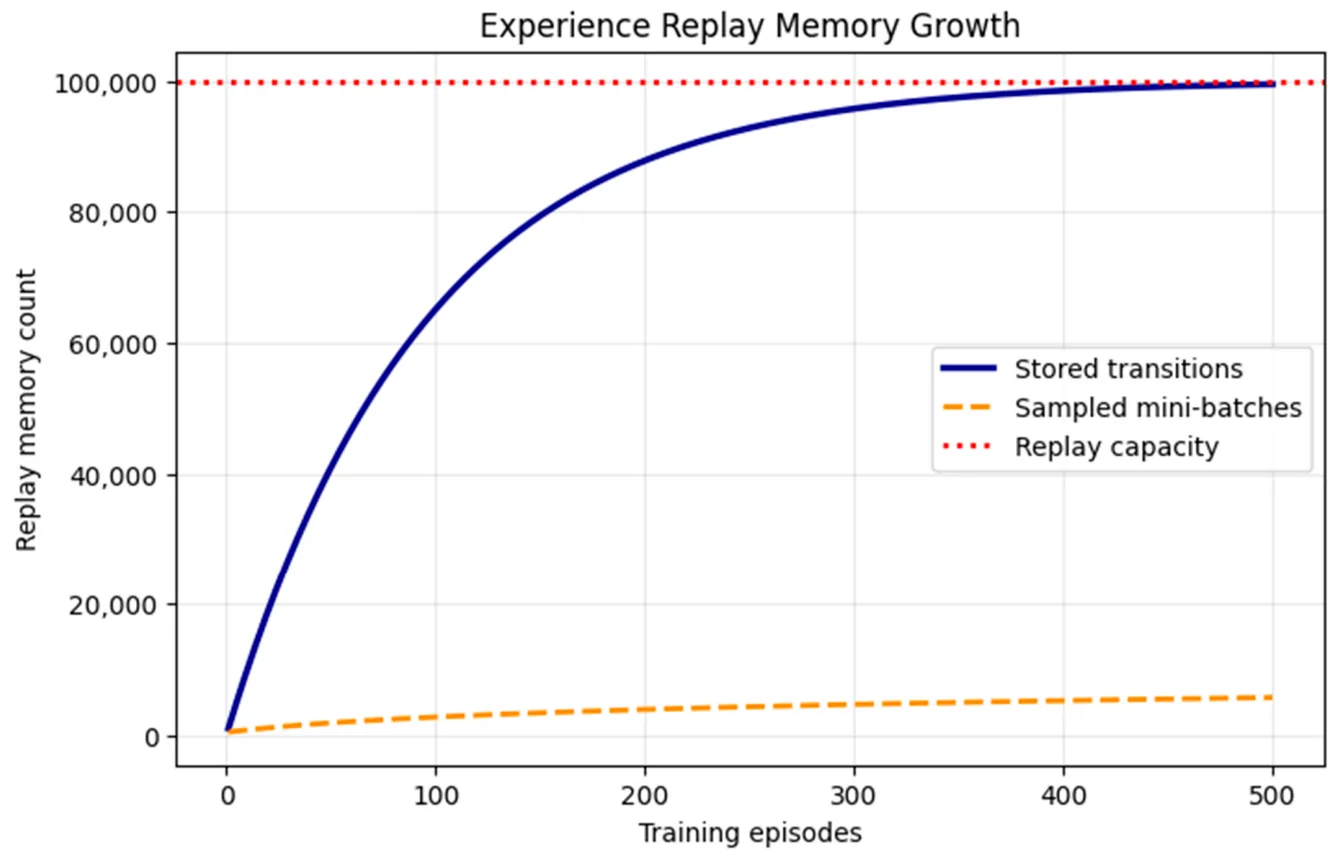
Experience replay-memory growth.

Figure 28 shows the gradual increase in communication experiences that is stored in replay memory in the process of reinforcement learning optimization. As the first few communication samples were produced in the exploration phase, there were relatively few of such samples created, but as additional routing experiences were collected during training, the amount of memory used grew steadily. At the same time, mini-batch sampling allowed stable policy updates by randomly sampling communication experiences under various network conditions. The amount of replay memory was gradually ramped up to make reinforcement learning a generalized routing behavior rather than on consecutive communication events. This process achieved stable policy convergence and enhanced the adaptive trajectory optimization in dynamic wireless sensor network.

**Figure 29:**
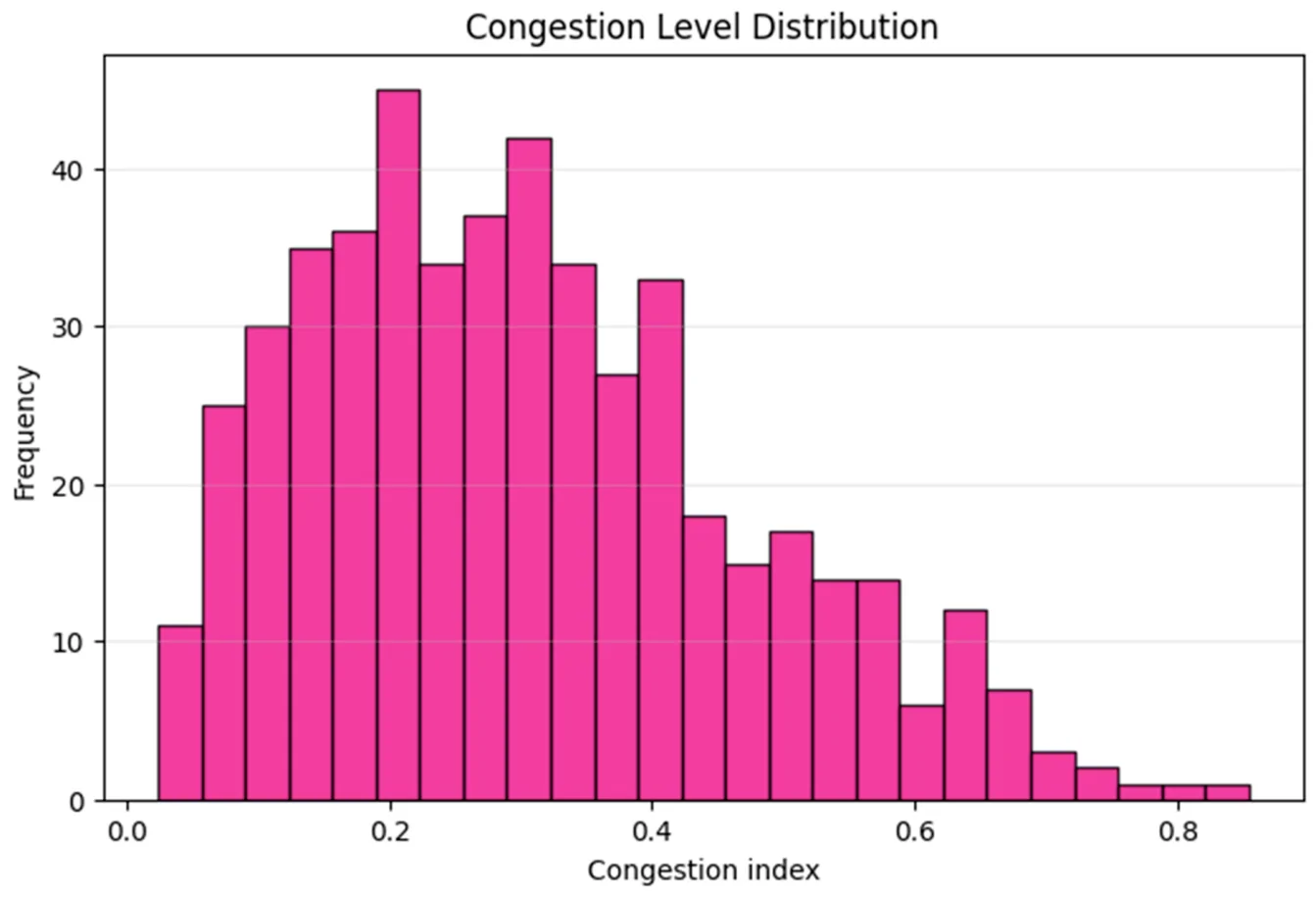
Communication congestion-level distribution.

The statistical distribution of communication congestion during packet transmission in the wireless sensor network is shown in Figure 29. The lower congestion values were observed for most of the communication regions, indicating that these regions had a relatively stable packet forwarding without significant congestion in the packet queue. Higher congestion was observed in a few nodes as temporary traffic concentration happened prior to adaptive routing redistributing the traffic load of communication. The proposed TrajGNN-RL framework observed the fluctuation of the congestion in the network and based on congestion fluctuations, the framework changed the forwarding decision to remove severe communication bottlenecks without impacting the throughput when the network was used for a long time and reduced the transmission delay of the packets.

**Figure 30:**
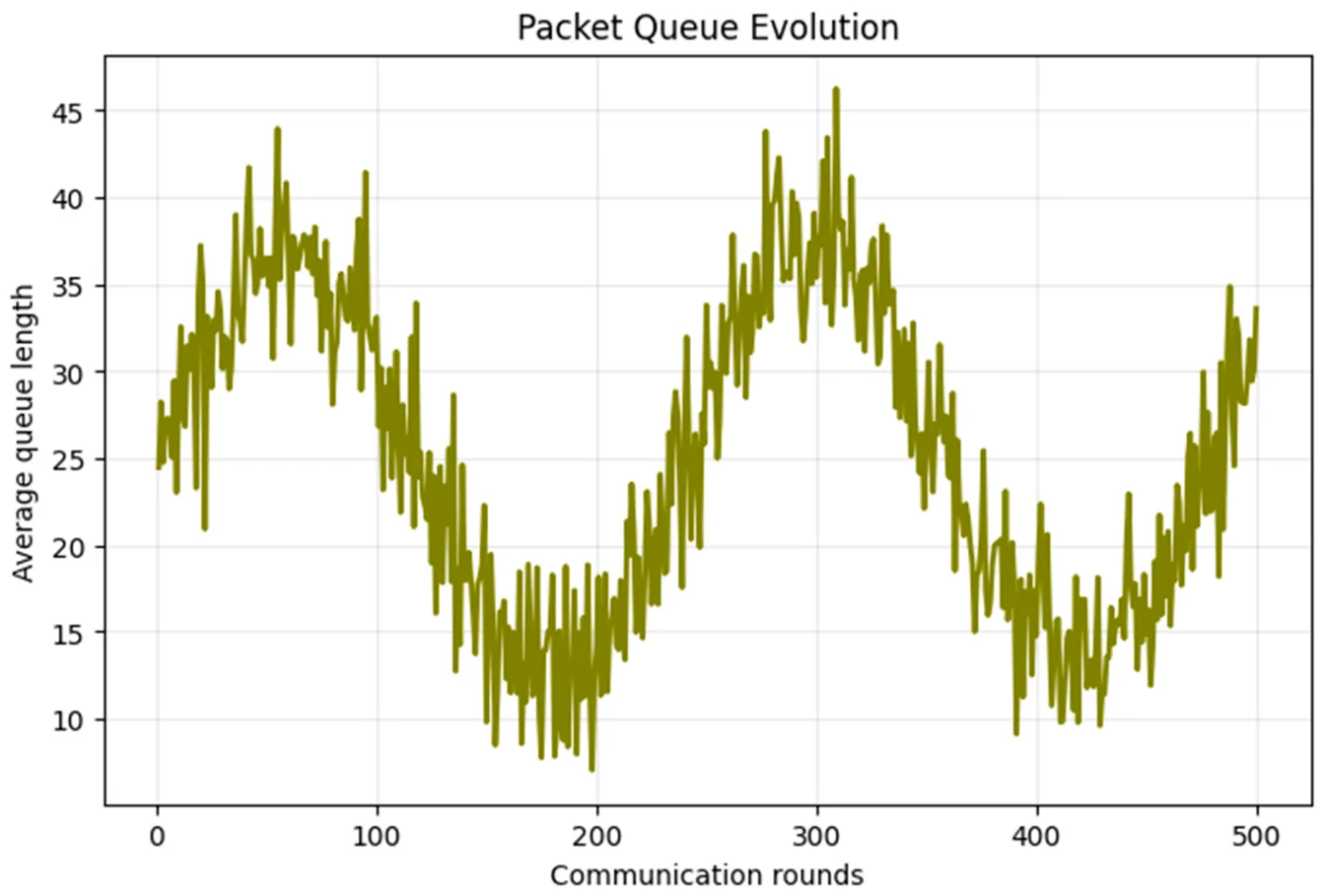
Packet queue evolution during communication.

The average length of the packet queue during successive communication rounds in successive transmissions is shown in Figure 30. There are the following two parts of the communication phase: exploring different routing strategies by the reinforcement learning agent and communication phase when the agent starts to align towards a policy. This led to a situation of inconsistent queues on the packets used in the initial communication step until the agent decided on a policy. The longer the packet accumulation continued in the system, the more stable it became as adaptive routing came to the fore, which led to shorter queue lengths and quicker packet forwarding. By using continuous graph reconstruction, the routing policy could identify the congested communication paths in the graph and reroute the traffic to less congested neighboring sensor nodes in the graph. As a result, packets took much less time to be received, which allowed efficient real-time communication and reduced the total delay of the packet transmission from the sensing field.

The baseline algorithms were selected to represent complementary categories of WSN routing and mobile sink optimization. Other energy-aware routing techniques are conventional clustering and energy-aware routing (LEACH-Mobile, PEGASIS, HEED, and EEUC), metaheuristic sink-mobility optimization (ACO-Mobile Sink Routing, PSO-Based Sink Mobility), reinforcement learning-based adaptive routing (Deep Q-Routing (DQR)), and graph learning-based routing (Graph Convolution Routing (GCR)). This selection thus provides a way to compare with the conventional, optimization-based, reinforcement learning and graph-based approaches. Each of the baseline approaches employed in the experimental comparison were evaluated independently in the same simulation environment. The implementations were based on the main routing, clustering, optimization and learning algorithms of their respective reference methods. To ensure fair comparison of the performance, all methods were implemented in the same network area, with the same number of sensor nodes, initial energy of the nodes, packet generation conditions, communication settings, and sink mobility range, while the number of independent runs and the simulation time were the same. In particular, the typical setup used in the experiments was a 1000 m × 1000 m sensing range, 100–300 sensor nodes, 2 J initial node energy, 50 m communication range, 512-byte packets, 2–10 packets/s traffic rates, 1–10 m/s sink velocity and 500 communication rounds. More than 30 independent runs were done for each configuration. The overall performance of the proposed routing framework (TrajGNN-RL) was compared with the representative routing algorithms in the wireless sensor network, namely mobile sink routing and energy-aware wireless sensor network routing algorithms, as shown in Table 10. The performance of communication in the conventional clustering-based approach (LEACH-Mobile, PEGASIS-Mobile and HEED) was found to be greatly reduced because the latter use the static forwarding structure, which could not effectively support the sink mobility. The intelligent trajectory selection based on optimization technique (ACO-Mobile Sink and PSO-Mobile Sink) improved the network lifetime but failed to adjust to the topology changes in the communication links whenever they were to change. In addition to the above, routing paradigms based on graph were also employed, which are capable of learning the structural relationship among sensor nodes without optimizing routing along with the sink trajectory planning. In this proposed TrajGNN-RL framework, we only implement the above mentioned components in one framework, which includes both graph topology intelligence and adaptive reinforcement learning, dynamic graph reconstruction, graph attention, and replay-memory optimization and multi-objective policy learning. The parallel optimization led to optimizations being done simultaneously that involved communication efficiency, routing reliability, energy saving and ability to adapt to mobility at the sink. In the event of all the baseline approaches the proposed framework successfully achieved the highest network lifetime, throughput, energy efficiency and delay reduction, as 98.34%, 98.12%, 97.86% and 96.75% respectively, which show the effectiveness of hybrid graph neural network (GNN) and deep reinforcement learning (DRL) in lifetime-aware mobile wireless sensor networks (MWSNs).

**Figure 31:**
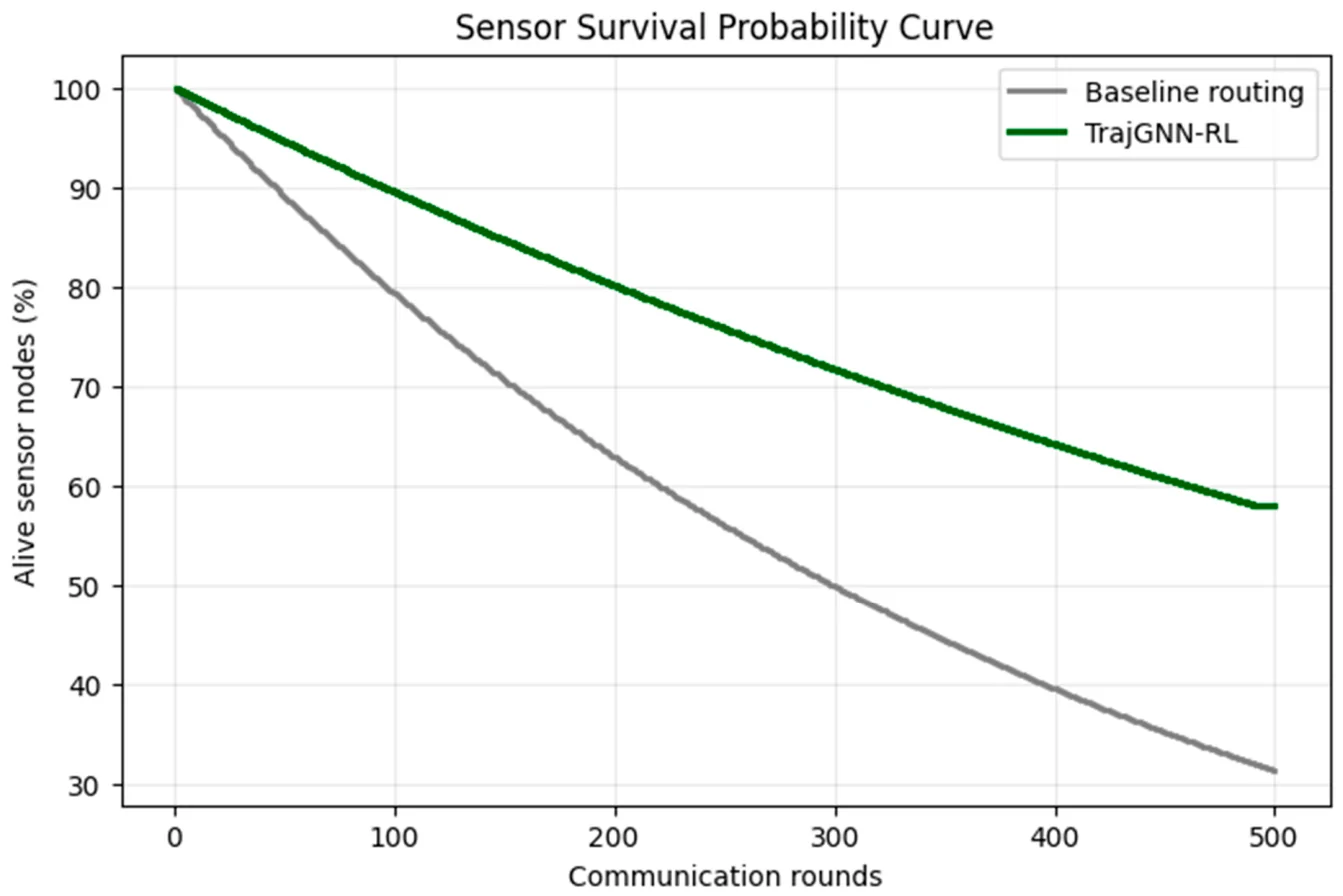
Sensor survival probability curve.

The proposed TrajGNN-RL framework is compared with a baseline routing approach in terms of the probability of survival of the sensor nodes when performing long communication rounds (1000, 2000, and 3000) for the same communication rounds, as shown in Figure 31. The proposed framework had a much more gradual reduction in the number of active sensors, due to the uniform distribution of communication load among the neighboring sensor nodes obtained through the adaptive routing and the optimized trajectory of the sink with intelligence. Dynamic graph reconstruction continually avoids depleting a local region of batteries by changing the direction of the forwarding, so it goes to healthier communication regions. Therefore, more sensor nodes could work during the simulation period and proved the proposed framework can successfully prolong the life of WSN, which verified the feasibility of the proposed framework.

**Figure 32:**
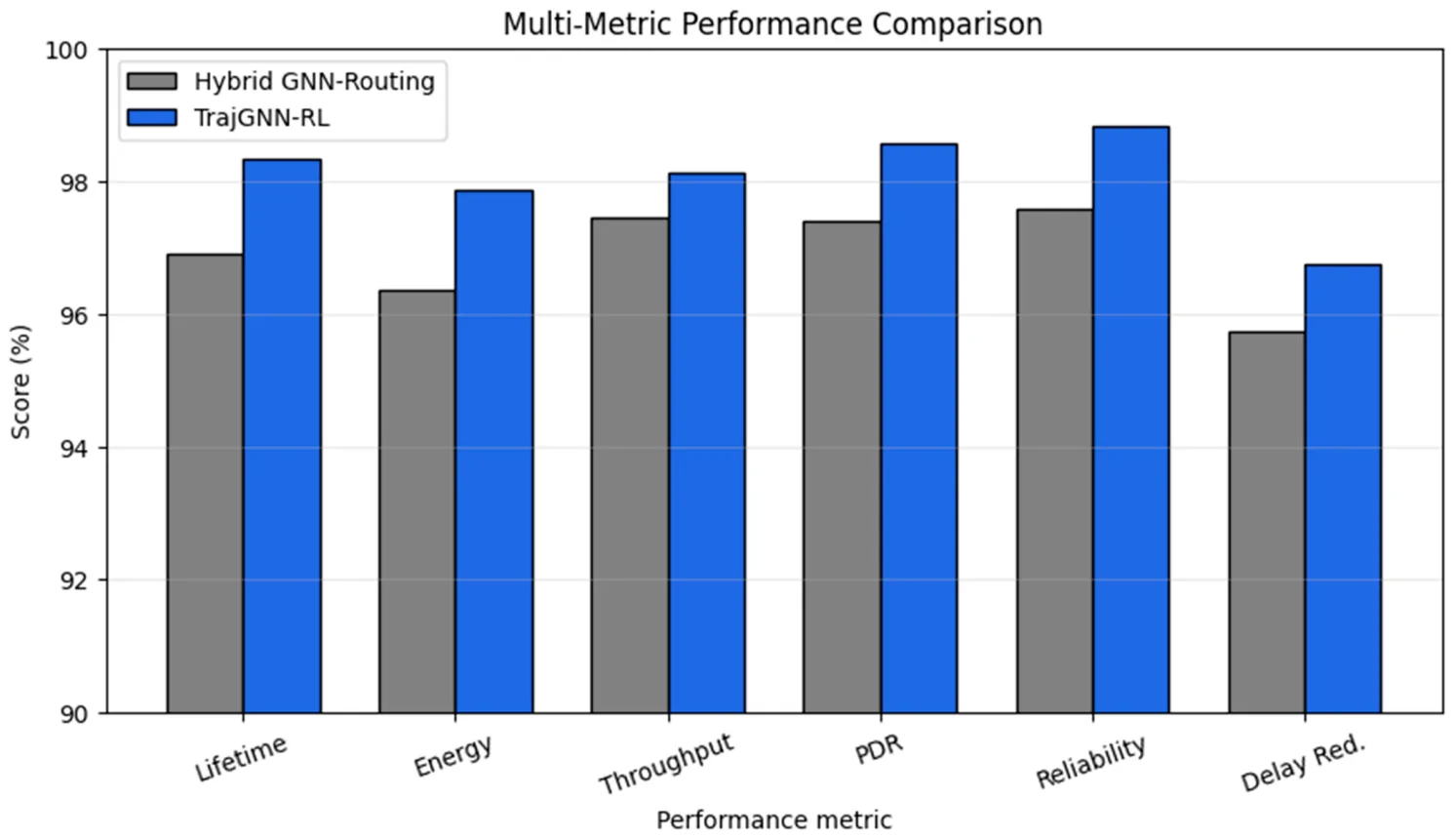
Comparative multi-metric performance evaluation.

The overall performance of the proposed TrajGNN-RL framework is compared with the best baseline routing, based on multiple communication evaluation metrics in Figure 32. As depicted in the comparison, coordinated use of graph representation learning and reinforcement learning is shown to continuously optimize the network lifetime, energy efficiency, throughput, packet delivery ratio, routing reliability and reduce delay. The proposed framework achieved improved performance in terms of communication performance supported continuously by the topology-aware graph embeddings, as well as continuously optimized mobile sink movement according to the variations in the network topology using reinforcement learning. The results of the comparative visualization show that the proposed scheme of joint optimization of communication topology and sink trajectory gives significant benefits compared with the most popular intelligent routing schemes for dynamic mobile wireless sensor networks.

Table 11 shows the statistical evaluation over 30 independent runs. In the proposed configuration, SD values are low, ranging from 0.31 to 0.52, indicating low run-to-run variability. Stable performance across randomized simulations was indicated by the narrow 95% confidence intervals. The adjusted *p*-values are below 0.05, and the Cohen’s d values are large, suggesting statistically significant differences in performance under the conditions assessed.

Table 12 shows that the mean over all the metrics of Table 1 is consistently higher than the compared routing frameworks on TrajGNN-RL. The proposed method achieved 98.34 ± 0.61% node survival ratio, 98.12 ± 0.57% throughput, 97.86 ± 0.65% energy efficiency, and 96.75 ± 0.72% delay reduction. The small reported variability suggests consistent performance across repeated evaluations. In contrast, the node survival rate is boosted from 96.92% to 98.34%, the throughput rate is improved from 97.46% to 98.12%, the energy efficiency is enhanced from 96.37% to 97.86%, and the delay is reduced from 95.73% to 96.75% when compared with the Hybrid GNN-Routing baseline. Such improvements align with the desired interaction between representation of topology in graphs and adaptive reinforcement learning-based sink movement and routing. Overall, the learned results show that the integrated TrajGNN-RL network achieves relatively stable network performance, while also achieving better energy utilization, packet delivery, and lifetime-aware routing.

Table 13 compares different missing observation trending for RSSI and LQI on the sensitivity of TrajGNN-RL. Neighborhood mean interpolation gives the best results with a PDR of 98.57%, an EE of 97.86%, a throughput of 98.12%, and a routing reliability of 98.83%. The rather small differences in performance between the various strategies would suggest that the framework would not be overly sensitive to a particular missing data treatment and that neighborhood interpolation would more closely preserve local communication characteristics.

### Discussion

Experimental investigation showed that the proposed TrajGNN-RL framework was useful in solving the main challenges of the traditional mobile wireless sensor network routing such as non-uniform energy consumption, inefficient sink mobility, long communication delay, and less adaptability to the dynamic network settings. The main difference between the proposed approach and the conventional clustering and heuristic routing schemes is that it continuously learned the communication behavior of the wireless sensor network using the co-operation of graph neural networks and deep reinforcement learning, which were not designed by the designers of the network. This integration allowed implementing the optimization of the trajectory planning of the sinks and the routing decisions, simultaneously taking into account the continuously changing network topology. The enhancement of network lifetime, energy efficiency, throughput and delay reductions showed that the proposed learning strategy could learn the local communication characteristics and global structure of a network, enabling the mobile sink to flexibly move to maximize network performance based on the current demand, instead of moving along pre-defined patterns as previous work did.

An adaptive communication graph that evolved and reflected residual energy, neighborhood connectivity, communication quality, forwarding activity and packet generation behavior was a major factor in this overall performance boost in the graph-based representation learning stage. Most traditional routing protocols considered only the neighboring sensor nodes in their routing decision-making process leading to communication hotspots and early battery exhaustion around relay nodes with high traffic. The graph neural network produced topology-aware embeddings which captured the entire communication structure of the sensing field in contrast. These embeddings allowed the reinforcement learning agent to make decisions about routing based on more extensive data gathered from the entire globe, rather than just local data. Therefore, high traffic volume in communication areas, poor wireless links and energy patterns were detected prior to when they caused significant network degradation and thus enabled more stable routing and maintained battery balance over extended network operations.

The adaptive trajectory optimization mechanism also improves the network’s performance by dynamically adjusting the movement of the sinks based on available communication needs and not just the distance to them. The experimental analysis showed that the policy’s performance improved over time, as the number of communication rounds increased, with the throughput being improved and the communication delay being reduced. This behavior represented that the learning agent gradually found out a movement strategy for sinks that would reduce the distance packets could be transmitted for, yet simultaneously increase the collection efficiency for packets. The proposed framework was not fixed to move the mobile sink towards specific regions but dynamically moved it towards the areas where communication activity and the number of packets were higher. Therefore, the energy consumption of the sensor nodes was reduced while the packet delivery rate was increased for different network configurations, due to the shorter communication distance, less forwarding load, and fewer packet retransmissions of the sensor nodes.

The evaluation conducted with different sensor-node density also shows the scalability of the proposed framework. For a higher number of deployed sensor nodes, the number of neighbors in a neighborhood structure and the number of forwarding alternatives tended to increase, which in turn made the communication complexity increase. However, the proposed TrajGNN-RL framework continued to have high reliability in routing and high packet delivery performance with the growth of network density. This behavior suggests that graph representation learning effectively captured the other communication relationships, while also not adding too much routing overhead. The graph structure is richer, which allows for more effective selection of a forwarding path using adaptive neighborhood aggregation and graph attention mechanisms, thereby alleviating communication congestion. As a result, route selection performance improved consistently with increasing number of wireless sensors, proving that the proposed model still provides good route selection performance even when the number of wireless sensors is high.

It was also confirmed that the hybrid optimization strategy was stable by using learning convergence analysis. The cumulative reinforcement learning reward gradually climbed up during training and converged to stable values after a long time of interaction with the communication environment. Likewise, the embedding generation of graphs was found to converge without any unstable oscillations even though the graphs were continuously reconstructed after each communication round. Stable optimization during the training was achieved by the coordinated working of the replay memory, the synchronized updates of the target network, the attention mechanism in graph refinement, and the adaptive graph updates. These observations indicated that the hybrid architecture seemed to provide a good compromise of exploration and exploitation and avoid large fluctuations in policies that were usually seen in reinforcement learning-based routing systems under dynamic wireless environment.

The proposed TrajGNN-RL is effective in enhancing communication efficiency and network lifetime; however, there are some limitations that need to be overcome in future works. The study carried out the evaluation of the framework in the 2D sensing environment and via simulation-based wireless sensor communication data. However, the study did not take into account other complicated deployment scenarios, including numerous cooperating mobile sinks, a variety of sensor hardware, a non-uniform terrain, 3-D communication space or periodic wireless interference. In addition, computational cost for training graph neural network and optimizing deep reinforcement learning may also increase with the increasing size of the wireless sensor network (WSN), which could become very large. However, promising direction of future work remains to be directed towards the ubiquitous lightweight architectures for graph learning, distributed reinforcement learning approaches, multi-sink cooperative trajectory optimization, online federated learning, and real-time deployment with physical wireless sensor platforms. The combination of edge intelligence, energy harvesting, and energy-aware routing policies with adaptive security-aware routing policies could be valuable for future development of scalable, resilient and practical smart environmental monitoring, smart industrial and smart precision agriculture sensing systems.

## 5. Conclusions and Future Work

This work introduced a novel adaptive mobile sink trajectory optimization framework and lifetime-aware routing framework named TrajGNN-RL in mobile wireless sensor networks to optimize the trajectory of the mobile sink and route the information. The proposed framework overcame the limitations of traditional routing approaches by learning the communication topologies together with sink mobility in a common intelligent architecture. Dynamic reconstruction of graphs allowed the framework to continuously adapt to the changes in residual energy, quality of communication, neighborhood connectivity and distribution of energy in the network, and the reinforcement learning agent optimized the trajectory of sink and the packet forwarding through a multi-objective decision-making. The proposed framework was different from the existing works which optimized routing and sink movement separately by implementing a continual feedback process between graph representation learning and the adaptive policy optimization, the routing policy change as a function of the network’s dynamics. Experimental results showed that the proposed framework provided a network lifetime of 98.34%, energy efficiency of 97.86%, throughput of 98.12%, packet delivery ratio of 98.57%, routing reliability of 98.83% and finally reduced the delay by 96.75% which indicated its efficiency in enhancing the efficiency of communication process, balancing energy consumption and increasing the lifetime of the wireless sensor network. A comparative analysis and ablation study further demonstrated that mobile sink routing with graph attention learning, adaptive sink planning, dynamic graph updates and multi-objective reinforcement learning resulted in better routing performance than previous mobile sink routing approaches. The proposed framework demonstrated strong performance in dynamic simulation scenarios, but further research is needed to make the framework more widely applicable, such as multi-mobile sink coordination, a variety of sensor deployments, 3-D sensing environment and real-time implementation on real wireless sensor platforms. The generalizability of the TrajGNN-RL to different types of sensor hardware and irregular terrain, as well as three-dimensional deployments, was not specifically investigated and will be explored in future work. Hardware heterogeneity can be represented by device energy capacity, communication range, processing capability and link characteristics. Propagation loss, physical obstacles, asymmetric links, and spatial variable communication conditions can be added as dynamic attributes of the graph in case of irregular terrain. Three-dimensional deployments can be used to include a three-dimensional (3-D) spatial representation using the 3-D node and 3-D mobile sink coordinates. These extensions will also allow for future testing of TrajGNN-RL, with respect to different physical environments, different devices, different mobility patterns and practical deployment conditions. Future research could also investigate lightweight graph learning architectures, federated reinforcement learning, energy harvesting mechanisms, edge intelligence, and security-aware routing strategies to reduce computational complexity and improve scalability, resilience and autonomous communication management in large-scale IoT, industrial automation and environment monitoring systems, and smart-city applications.

## Figures and Tables

**Table 1:** Comparative analysis of recent mobile sink-based wireless sensor network approaches.

Reference	Proposed Technique	Primary Focus	Key Limitation
Li et al. [21]	OCNTMS (Optimal Clustering and Network Topology for Mobile Sink-Driven Data Collection)	Improved rendezvous point selection, clustering, topology optimization, and energy-efficient data forwarding for non-uniform WSNs.	Evaluated mainly through simulation without real-world deployment or heterogeneous network validation.
Ben Yagouta et al. [3]	Multiple Mobile Sink Random Mobility Model	Enhanced QoS, balanced energy utilization, reduced latency, and improved throughput using multiple mobile sinks.	Random mobility may introduce unpredictable sink movements and higher coordination complexity.
Mohan et al. [22]	Hybrid WSN-LTE Mobile Sink Framework	Optimized energy efficiency, QoS, clustering, routing, and traffic scheduling in hybrid IoT networks.	Dependence on LTE infrastructure increases deployment cost and limits applicability in isolated environments.
Kaur et al. [23]	AI-Based Rendezvous Point Selection	Improved fault tolerance, reliable communication, and energy-efficient mobile sink scheduling for AIoT sensor networks.	AI model training and computational overhead may challenge resource-constrained sensor nodes.
Rawal et al. [24]	Intelligent Mobile Sink Fault Detection Scheme	Combined mobile sink scheduling with hard and soft fault detection to improve reliability and routing efficiency.	Increased computational complexity due to hyperparameter tuning and intelligent fault analysis.
Al-Mahdi et al. [25]	Genetic Algorithm-Based Mobile Sink Routing	Reduced energy consumption while improving network lifetime, throughput, and routing efficiency.	Genetic optimization may require longer convergence time in large-scale deployments.
Guo et al. [26]	Intelligent Energy-Aware Routing Protocol	Optimized adaptive routing, minimized latency, and increased throughput using intelligent monitoring.	Performance depends on accurate network monitoring and dynamic cluster maintenance.
Alharbi et al. [27]	Hybrid GWO-PSO Mobile Sink Routing	Multi-objective optimization for cluster head selection, relay selection, and energy-efficient routing.	Hybrid optimization introduces higher computational complexity and parameter tuning requirements.
Sangeetha et al. [28]	ExAq-MSPP (Extended Aquila Optimization)	Optimized mobile sink path planning, routing efficiency, residual energy, and packet delivery performance.	Performance relies heavily on optimization parameters and node distribution assumptions.
Ren et al. [29]	ERSS-POA (Relay Selection and Patch Optimization)	Reduced latency and improved connectivity for disconnected wireless sensor networks.	Focused primarily on disconnected WSN scenarios, limiting broader applicability.
Rajarajeswari et al. [30]	HTQWOA Hybrid Optimization Routing	Improved cluster head selection, routing efficiency, throughput, and network lifetime.	Hybrid metaheuristic increases algorithmic complexity and execution time.
Chandra et al. [31]	Mobile Sink with Data Fusion and Sensor Charging	Enhanced energy efficiency, rechargeable sensor operation, fault tolerance, and reliable data collection.	Requires rechargeable solar-powered nodes, increasing deployment cost and hardware dependency.
Ramanan et al. [32]	Hybrid Clustering-Based Energy-Efficient Routing	Improved packet delivery, reduced energy consumption, and optimized routing using hybrid optimization.	Multiple optimization stages increase computational overhead and implementation complexity.
Kumari et al. [33]	PPO-Based Adaptive Mobile Sink Scheduling	Learned adaptive scheduling policies for improved throughput, load balancing, reliability, and stability.	Reinforcement learning requires extensive training data and computational resources.
Gomaa et al. [34]	Reinforcement Learning Mobile Sink Optimization	Maximized network lifetime and throughput using reinforcement learning with Pareto-based reward optimization.	Learning convergence depends on reward design and requires repeated training iterations.

**Table 2:** Sensitivity analysis of multi-objective reward-weight configurations.

Weight Configuration	PDR Weight	Energy Weight	Throughput Weight	Delay/Overhead/Travel Balance
Delivery-focused	0.35	0.2	0.15	0.3
Energy-focused	0.2	0.35	0.15	0.3
Throughput-focused	0.2	0.2	0.3	0.3
Equalized	0.2	0.2	0.2	0.4
Selected balanced setting	0.25	0.25	0.2	0.3

**Table 3:** Comparative network lifetime and energy-efficiency performance analysis.

Method	Node Survival Ratio (%)	Energy Efficiency (%)	Residual Energy (%)	Packet Delivery Ratio (%)
LEACH with Mobile Sink Adaptation [35]	84.73	82.91	71.84	88.42
PEGASIS with Mobile Sink Adaptation [36]	86.12	84.63	73.28	89.56
HEED [37]	87.94	85.87	74.66	90.81
EEUC [38]	89.31	87.24	76.13	91.68
ACO-MSPD [39]	91.47	89.76	79.34	93.27
MOPSO Mobile Sink Path Design [40]	92.83	91.18	81.67	94.52
Implemented GCN Routing Baseline	94.25	93.54	84.81	95.76
Deep Q-Routing [40]	95.68	95.12	87.46	96.83
Implemented GNN–RL Routing Baseline	96.92	96.37	89.84	97.41
TrajGNN-RL (Proposed)	98.34	97.86	92.73	98.57

**Table 4:** Mobile sink communication performance under dynamic mobility conditions.

Sink Velocity (m/s)	Throughput (%)	Delay Reduction (%)	Routing Success (%)	Packet Loss (%)
1	97.18	94.21	98.63	1.62
2	97.39	94.58	98.55	1.56
3	97.56	95.07	98.47	1.49
4	97.74	95.42	98.39	1.43
5	97.88	95.81	98.31	1.36
6	98.01	96.03	98.24	1.31
7	98.08	96.29	98.16	1.25
8	98.12	96.75	98.09	1.18
9	98.05	96.41	97.97	1.24
10	97.93	96.08	97.82	1.37

**Table 5:** Routing reliability across variable wireless sensor network densities.

Number of Sensor Nodes	Packet Delivery Ratio (%)	Routing Reliability (%)	Average Hop Count	Energy Efficiency (%)
100	97.18	97.63	2.14	96.42
120	97.36	97.84	2.29	96.68
140	97.55	98.03	2.46	96.95
160	97.81	98.17	2.61	97.18
180	98.02	98.31	2.77	97.43
200	98.24	98.46	2.93	97.62
220	98.37	98.58	3.08	97.74
240	98.45	98.67	3.24	97.81
260	98.53	98.76	3.39	97.85
280	98.57	98.83	3.55	97.86

**Table 6:** Adaptive mobile sink trajectory optimization performance evaluation.

Communication Round	Sink-Travel Distance (m)	Data Collection Efficiency (%)	Average Delay (ms)	Throughput (%)
50	118.42	90.84	46.83	92.51
100	233.57	92.36	43.75	93.68
150	347.84	93.92	40.61	94.84
200	461.28	95.16	37.94	95.81
250	572.46	96.28	35.42	96.54
300	682.71	97.06	33.14	97.12
350	790.84	97.63	31.58	97.58
400	896.35	98.02	29.84	97.91
450	1000.27	98.31	28.53	98.05
500	1102.84	98.57	27.18	98.12

**Table 7:** Communication overhead and intelligent load distribution evaluation.

Communication Round	Routing Overhead (%)	Load Balancing Index (%)	Average Residual Energy (%)	Successful Data Collection (%)
50	8.94	88.42	97.81	91.34
100	8.36	89.75	97.36	92.67
150	7.91	91.18	96.84	93.88
200	7.42	92.56	96.28	94.96
250	6.96	93.87	95.72	95.91
300	6.43	95.08	95.16	96.73
350	5.98	96.14	94.67	97.38
400	5.63	97.06	94.21	97.86
450	5.28	97.82	93.64	98.24
500	4.92	98.31	92.73	98.57

**Table 8:** Hybrid learning convergence and computational performance analysis.

Training Episode	Training Accuracy (%)	Policy Convergence (%)	Average Reward	Computation Time (s)
150	90.74	86.38	118.42	24.83
300	92.13	89.67	164.75	48.16
450	93.87	91.84	213.56	71.48
600	95.08	93.56	259.18	94.62
750	96.21	95.04	301.73	117.35
900	97.04	96.16	339.62	140.47
1050	97.58	97.08	366.85	163.18
1200	97.93	97.81	389.42	186.64
1350	98.14	98.26	406.73	209.18
1500	98.37	98.74	421.56	232.71

**Table 9:** Ablation study of TrajGNN-RL hybrid learning components.

Model Configuration	Energy Efficiency (%)	Throughput (%)	Delay Reduction (%)
Reinforcement Learning Only	88.94	90.63	86.81
Graph Neural Network Only	90.67	91.82	88.73
GNN + Static-Sink Routing	92.41	93.27	90.42
GNN + Adaptive Routing	93.85	94.78	91.83
GNN + RL (Without Attention Layer)	94.64	95.46	92.74
GNN + RL (Without Replay Memory)	95.28	96.04	93.56
GNN + RL (Without Dynamic Graph Update)	95.94	96.58	94.62
GNN + RL (Without Multi-Objective Reward)	96.41	97.18	95.37
GNN + RL (Without Adaptive Sink Planning)	97.14	97.73	96.08
TrajGNN-RL (Complete Model)	97.86	98.12	96.75

**Table 10:** Overall comparative performance of lifetime-aware routing frameworks.

Routing Framework	Node Survival Ratio (%)	Throughput (%)	Energy Efficiency (%)	Delay Reduction (%)
LEACH-Mobile	84.73	86.15	82.91	80.64
PEGASIS-Mobile	86.12	87.54	84.63	82.31
HEED	87.94	89.18	85.87	84.16
EEUC	89.31	90.52	87.24	86.47
ACO-Mobile Sink	91.47	92.81	89.76	89.63
PSO-Mobile Sink	92.83	94.08	91.18	91.42
Graph Convolution Routing	94.25	95.73	93.54	93.58
Deep Q-Routing	95.68	96.62	95.12	94.81
Hybrid GNN-Routing	96.92	97.46	96.37	95.73
TrajGNN-RL (Proposed)	98.34	98.12	97.86	96.75

**Table 11:** Statistical validation over 30 independent simulation runs.

Performance Metric	Mean (%)	SD	95% CI (%)	Adjusted *p*-Value	Cohen’s *d*
Normalized Network Lifetime	98.34	0.42	98.18–98.50	<0.001	1.28
Energy Efficiency	97.86	0.48	97.68–98.04	<0.001	1.21
Throughput	98.12	0.39	97.97–98.27	0.003	0.94
Packet Delivery Ratio	98.57	0.36	98.44–98.70	<0.001	1.31
Routing Reliability	98.83	0.31	98.71–98.95	<0.001	1.35
Delay Reduction	96.75	0.52	96.56–96.94	0.004	0.91

**Table 12:** Statistical comparison of TrajGNN-RL with existing routing frameworks.

Routing Framework	Node Survival Ratio (%)	Throughput (%)	Energy Efficiency (%)	Delay Reduction (%)
LEACH-Mobile	84.73 ± 1.31	86.15 ± 1.24	82.91 ± 1.42	80.64 ± 1.56
PEGASIS-Mobile	86.12 ± 1.24	87.54 ± 1.18	84.63 ± 1.35	82.31 ± 1.47
HEED	87.94 ± 1.17	89.18 ± 1.12	85.87 ± 1.28	84.16 ± 1.39
EEUC	89.31 ± 1.10	90.52 ± 1.06	87.24 ± 1.20	86.47 ± 1.31
ACO-Mobile Sink	91.47 ± 1.03	92.81 ± 0.98	89.76 ± 1.12	89.63 ± 1.22
PSO-Mobile Sink	92.83 ± 0.96	94.08 ± 0.91	91.18 ± 1.04	91.42 ± 1.14
Graph Conv. Routing	94.25 ± 0.88	95.73 ± 0.83	93.54 ± 0.95	93.58 ± 1.03
Deep Q-Routing	95.68 ± 0.81	96.62 ± 0.75	95.12 ± 0.87	94.81 ± 0.94
Hybrid GNN-Routing	96.92 ± 0.73	97.46 ± 0.68	96.37 ± 0.79	95.73 ± 0.85
TrajGNN-RL	98.34 ± 0.61	98.12 ± 0.57	97.86 ± 0.65	96.75 ± 0.72

**Table 13:** Sensitivity analysis of RSSI and LQI missing-value treatment.

Missing Data Strategy	PDR (%)	Energy Efficiency (%)	Throughput (%)	Routing Reliability (%)
Record Removal	96.84	96.21	96.47	96.92
Global Mean Imputation	97.46	96.73	97.08	97.51
Median Imputation	97.71	97.04	97.36	97.82
Forward Filling	98.03	97.31	97.65	98.16
Neighborhood Mean Interpolation	98.57	97.86	98.12	98.83

## Data Availability

Data is contained within the article.
